# Double FIT hybridization probes – towards enhancing brightness, turn-on and specificity of RNA detection†

**DOI:** 10.1039/d3sc00363a

**Published:** 2023-03-23

**Authors:** Sophie Schöllkopf, Andrea Knoll, Amal Homer, Oliver Seitz

**Affiliations:** a Institut für Chemie, Humboldt-Universität zu Berlin 12489 Berlin Germany oliver.seitz@hu-berlin.de

## Abstract

Efficient fluorogenic hybridization probes combine high brightness and specificity of fluorescence signaling with large turn-on of fluorescence. Herein, we present an approach to enhance signaling by combining two identical fluorescence base surrogates in FIT^2^ probes. Provided there is a suitable positioning of dyes, target-bound FIT^2^ probes emit brighter than mono dye probes, while dye–dye contact in the single stranded state provides opportunities for decreasing background fluorescence. The probes were used to explore the single nucleotide-specific detection of a C → U edited RNA of the glycine receptor (GlyR). We observed strong self-quenching upon single base mismatched hybridization of FIT^2^ probes, which helped in distinguishing edited from unedited RNA target in cell lysates.

## Abstract 2

Efficient fluorogenic hybridization probes combine high brightness and specificity of fluorescence signaling with large turn-on of fluorescence.

## Graphical abstract

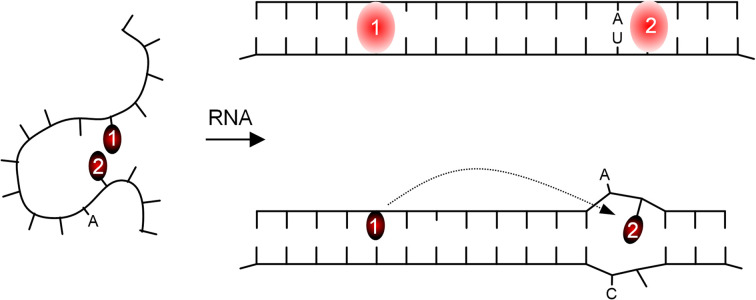

Fluorogenic oligonucleotide hybridization probes are extremely versatile and enable the direct detection and localization of specific DNA/RNA molecules within complex mixtures such as in cells, cell lysates or PCR-type applications.^1–3^ The performance of a fluorogenic probe is determined by three key characteristics; (i) turn-on, (ii) brightness and (iii) sequence specificity of fluorescence signalling. An ideal probe would excel in all three performance characteristics. However, in practice, usually only one or two of these criteria can be optimized with a given probe technology. For example, with molecular beacon-type probes^4^ relying on the distance-dependent interactions between two dyes it is relatively easy to optimize the turn on by spectral matching of fluorescence donors and acceptors, often at the cost of brightness, or by optimizing aggregation of dyes.^5–11^ Sequence specificity of fluorescence signalling, on the other hand, typically is limited by the fidelity of Watson–Crick base pairing. Considering that oligonucleotide probes must have a sufficient length to provide uniqueness and sufficient affinity, it can be difficult to discriminate targets that differ, for example, by a single nucleotide. This issue is most pressing when single nucleotide-specific measurements ought to be performed at the comparatively low temperatures required for measurements in cells or cell lysates.

Focussing on high sequence-specificity of fluorescence signalling, we have introduced the Forced Intercalation (FIT) probes (Fig. 1A).^12–17^ In these PNA-, DNA- or RNA-type oligomers, a fluorophore belonging to the thiazole orange family of cyanine dyes replaces a canonical nucleobase, distinguishing them from Light-Up probes^18^ and Echo probes,^6^ which tether thiazole orange *via* flexible tethers at the periphery. The fluorescent base surrogate senses viscosity changes in the immediate environment. In a low viscosity environment TO is virtually non-fluorescent because rotations around the central methine bridge deplete the TO excited state.^19^ Formation of the probe-target complex places the “TO base” in the double helical base stack. This high viscosity environment renders the TO fluorescent. Adjacent single base mismatches decrease the local viscosity, causing decreases of TO emission.^14,20^ As a result, FIT probes provide for single nucleotide specific fluorescent signalling regardless of hybridization fidelity. This property has facilitated the real-time detection of single nucleotide alterations in *in vitro* assays^21,22^ and fluorescence microscopic imaging.^23–30^ The FIT probe concept has also been applied for detection of double strands *via* triplex formation,^31–33^ and in signalling aptamers.^34^ To improve turn-on and brightness of fluorescence signalling by FIT-probes, we employed the artificial “thiazole orange base” as energy donor in combination with a second dye serving as energy acceptor.^35^ However, this affects the single nucleotide specificity of fluorescence signalling, because the “TO base” will transfer energy to the acceptor dye regardless of its sequence environment (matched or mismatched). To enable detection of C → U edited RNA, the approach was applied to a binary probe system, in which target specificity was determined by hybridization fidelity.^36^

**Fig. 1:**
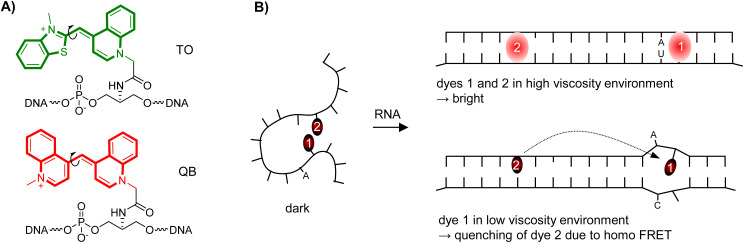
(A) DNA FIT-probe containing thiazole orange (TO) or quinoline blue (QB) as fluorescent base surrogate. (B) Principle of the FIT^2^-approach. The flexible single strand can adopt conformations which allow contact between dye 1 and dye 2. Hybridization with target enforces intercalation and separation of dye nucleotides leads to bright fluorescence. Homo FRET allows quenching when one of the dyes has mismatched base neighbours (B).

Looking for a way to improve hybridization-induced fluorescence enhancements and brightness of fluorescence signalling by FIT probes while maintaining or even increasing target specificity, we considered the combined use of two identical fluorescent base surrogates (Fig. 1B). The introduction of a second fluorescent base surrogate into a FIT probe can, on the one hand, increase the brightness of fluorescence in the target-bound state. On the other hand, dye–dye interactions provide options for decreasing the fluorescence intensity. For a FIT^2^ probe such decreases could occur when

(1) None of the TO-like dyes is embedded in the high viscosity environment provided by matches of adjacent base pairs.

(2) The two dyes form weakly fluorescing H-aggregates.

(3) One TO-like dye in a low viscosity environment quenches the other dye *via* homo FRET or

(4) Both dyes engage in excitonic interactions.

Options (2)–(4) cannot exist in single dye FIT probes explaining why single stranded FIT^2^ probes could potentially show lower fluorescence than expected for the sum of the fluorescent components. Hybridization will prevent options (1) and (2) making it likely that fluorescence should increase strongly upon binding of target. Highest fluorescence enhancements should be obtainable when options (3) and (4) remain unutilized in the probe-target duplex. This should be the case when the distance between the dyes is high and/or orientation of the dyes does not permit coupling of transition dipole moments. For example, according to molecular ruler work from Asanuma^37^ and our work,^35^ a 8 nt distance minimizes energy transfer between dyes embedded in the base stack of a DNA–DNA duplex. Given an optimal positioning, FIT^2^ probes could therefore provide higher fluorescence intensity and higher turn-on upon hybridization than single dye FIT probes. We furthermore hypothesized that a single base mismatch in the vicinity of one dye will weaken the stacking interactions and allow the dye to adopt conformations that permit energy transfer. As a result, fluorescence signalling by FIT^2^ probes may discriminate single base mismatches under non-stringent hybridization conditions.

Herein we describe FIT^2^ probes that enable the single nucleotide specific call for a nucleotide corresponding to the C → U editing site in RNA from a transcript encoding a glycine receptor (GlyR). Measurements in highly viscous cell lysates demonstrate that two identical TO-type base surrogates within a FIT^2^ probe can indeed discriminate better against single base mismatches than mono FIT probes.

## Results and discussion

**Fig. 2:**
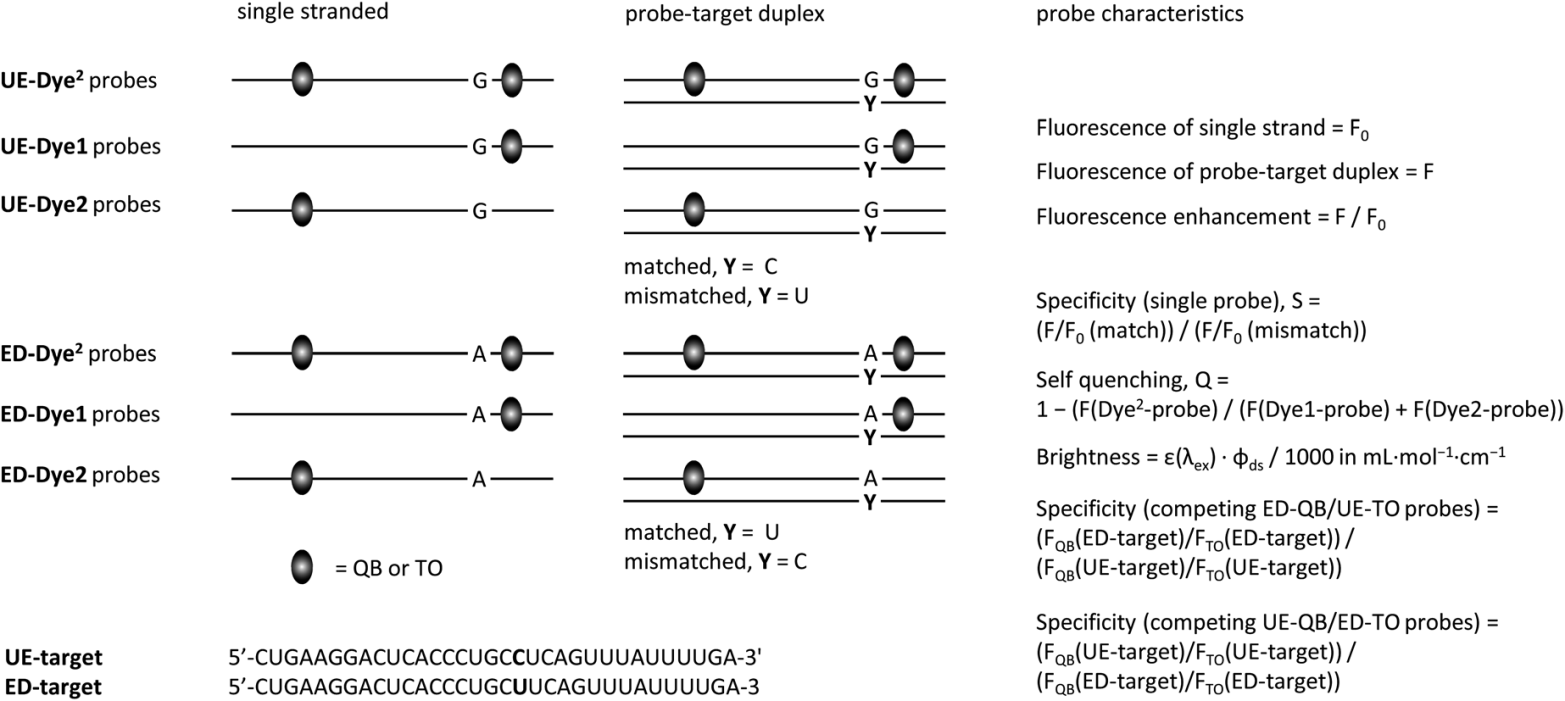
Graphical depiction of hybridization experiments and probe characteristics assessed in Table 1, Fig. 4 and 5.

We designed FIT probes and FIT^2^ probes to enable homogeneous detection of GlyR mRNA (Fig. 2).^38^ Probes were 16–18 nt long to guarantee sequence uniqueness but also to provide options for the introduction of a second base surrogate. The QB fluorescent base surrogate,^26,28^ a particularly responsive member of the TO-family, was placed next to the editing site. For DNA–DNA duplexes, in which fluorescent bases surrogates were linked *via* serinol or threoninol units, it was reported that FRET is low when two base-stacked dyes are separated by 8 spacer nucleotides.^35,37^ Given the compressed structure of RNA duplexes, we slightly extended the distance range and positioned the second QB base also 9 spacer nucleotides away. To increase brightness of fluorescence, we introduced a locked nucleotide (LNA) as QB next neighbour, as previously described.^25^

**Fig. 3:**
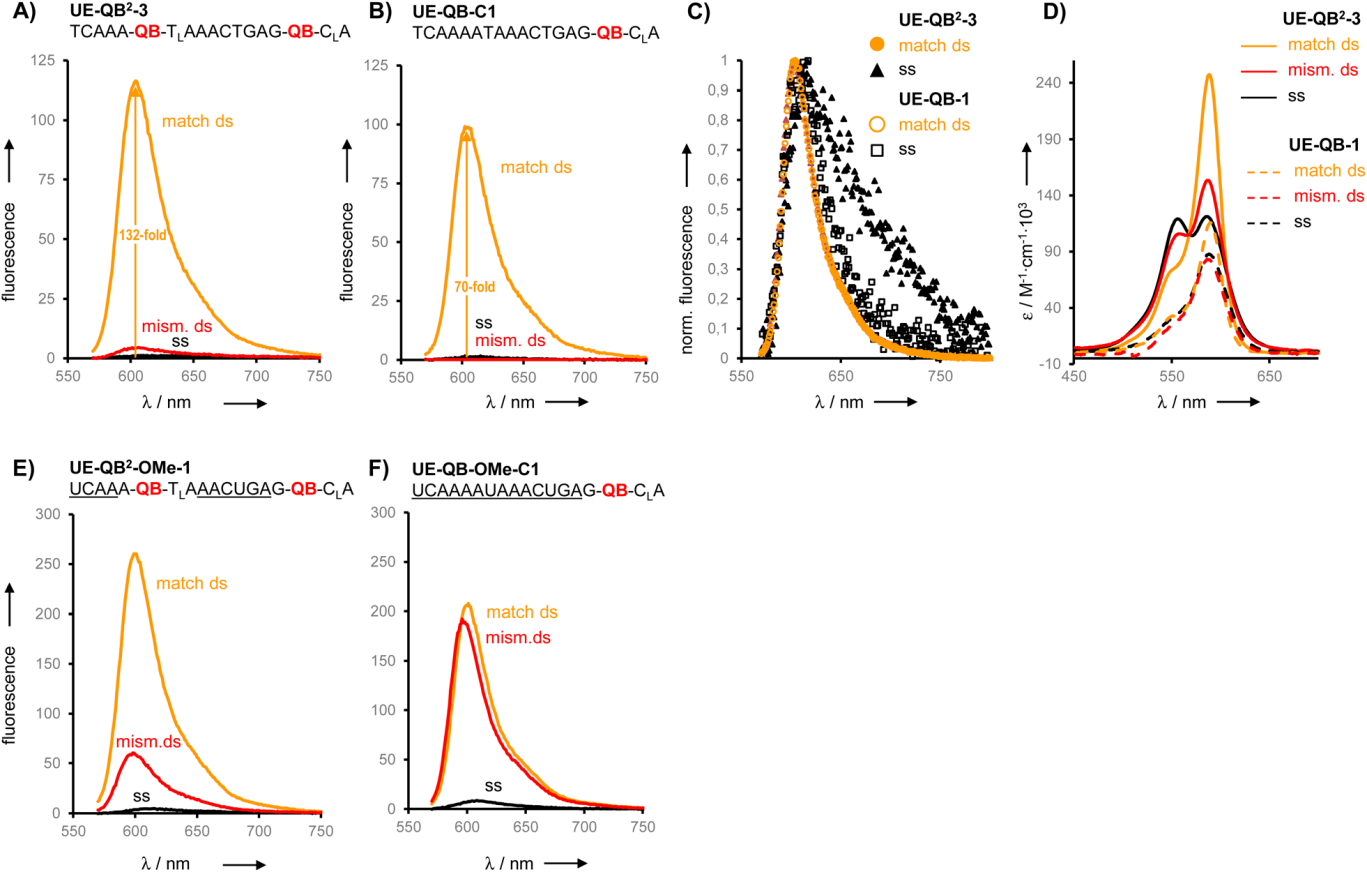
(A), (B), (C), (E), (F) Emission and (D) absorption spectra of (A), (C), (D) dual QB FIT^2^ DNA probe UE-QB^2^-3 and (B), (C), (D) mono QB FIT DNA probe UE-QB-C1 and (E) FIT RNA probe UE-QB^2^-OMe-1 and (F) FIT RNA probe UE-QB-OMe-C1 in the single stranded state (black) and after hybridization with matched (orange) RNA and single mismatched (red) RNA target (5′-CUGAAGGACUCACCCUGCYUCAGUUUAUUUUGA-3′; UE-RNA: Y = C, ED-RNA: Y = U). (C) Normalized emission spectra before and after matched hybridization. Underlined = 2′OMe-RNA; subscript L = LNA. Conditions: 0.5 μM FIT probe, 2.5 μM target RNA (if added) in buffer (100 mM NaCl, 10 mM Na_2_HPO_4_, pH 7), 37 °C (in (A)–(C)) or 25 °C (in (D)–(F)) *λ*_ex_ = 560 nm. Emission spectra were measured at identical slit widths for FIT^2^ probes and FIT probes.

We prepared six different QB^2^ FIT probes targeting the unedited state of GlyR mRNA (UE-QB^2^-1–UE-QB^2^-6, Table 1). In the absence of target, the probes were only weakly fluorescent. Notable 63-125-fold enhancements of fluorescence were obtained upon hybridization with matched RNA target. The intensity of fluorescence emission from a dual QB duplex such as UE-QB^2^-3 (Fig. 3A, see also Fig. S1†) was slightly higher than from the corresponding mono-QB probe UE-QB-C1 (Fig. 3B). The shapes of the emission spectra were superimposable (Fig. 3C) indicating that dye–dye contact did not occur in the duplex state. Noteworthy differences were observed for the single stranded state of the probes. Albeit weak, dual QB probes such as UE-QB^2^-3 showed a markedly broad emission band, which extended to the red spectral region (Fig. 3C, see also Fig. S6†). Such shifts are typical hallmarks of excimers and/or H-aggregates, the latter being mostly only weakly fluorescent.^39,40^ In accordance with this interpretation, absorption spectra of single stranded probes revealed a second band at 556 nm, blue-shifted from the original peak at 586 nm (Fig. 3D, see also Fig. S1–S5†). As expected, this band disappeared upon binding of the matched target. Concomitantly, the absorption band at 586 nm increased in intensity. Similar effects have been observed previously for the DNA stain TOTO and other TO dimers.^41–44^ Perhaps surprisingly, the blue-shifted band can remain when the probe was hybridized with the single mismatched target corresponding to the edited GlyR mRNA state. At first glance, it seems unlikely that H-aggregates or excimers can form when the melting temperature of the single mismatched UE-QB^2^-3-target duplex (*T*_M_ = 48.7 °C, Fig. S7†) is significantly higher than the temperature during fluorescence measurements. However, UV melt studies revealed rather shallow transitions for dual dye probes (Fig. S7†) which may indicate that dual dye probes undergo local dissociation already at temperatures below the *T*_M_. Most importantly, fluorescence of single base mismatched duplexes was low and the specificity *S* (for definition see Fig. 2) remained high (Table 1). This suggests that the QB dye in the mismatched environment can indeed quench the other QB dye.

**Table 1:** Fluorescence properties and *T*_M_ data of FIT^2^ and FIT probes

		*F* a		*F*/*F*_0_^b^		*S* c		*T*_M_ d/°C	Br^e^ match
		25 °C	37 °C	25 °C	37 °C	25 °C	37 °C		
UE-QB^2^-1	AAAQBT_L_AAACTGA*G*QBC_L_A	157	92	63	66	7	26	49.9	13.5
UE-QB^2^-2	CAAAQBT_L_AAACTGA*G*QBC_L_A	174	110	71	107	6	29	51.2	13.1
UE-QB^2^-3	TCAAAQBT_L_AAACTGA*G*QBC_L_A	176	116	92	132	7	30	52.0	12.3
UE-QB^2^-4	AAAATQBA_L_ACTGA*G*QBC_L_A	139	66	98	82	15	60	49.6	8.9
UE-QB^2^-5	CAAAATQBA_L_ACTGA*G*QBC_L_A	157	82	124	110	9	46	48.3	10.3
UE-QB^2^-6	TCAAAATQBA_L_ACTGA*G*QBC_L_A	157	84	125	121	11	48	49.2	11.0
UE-QB-C1	TCAAAATAAACTGA*G*QBC_L_A	152	99	46	70	16	58	48.4	12.7
UE-QB-C2	TCAAAQBT_L_AAACTGA*G*GCA	78.7	49	22	26	3	9	49.8	7.4
UE-QB-C3	TCAAAATQBA_L_ACTGA*G*GCA	110	61	48	47	4	19	49.2	10.3
UE-QB^2^-OMe-1	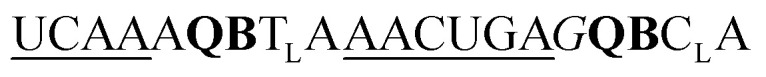	243	156	55	125	4	14	49.6	29.4
UE-QB-OMe-C1	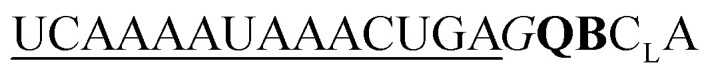	194	142	23	85	1.3	1.2	62.1	23.1
UE-TO^2^-OMe-1	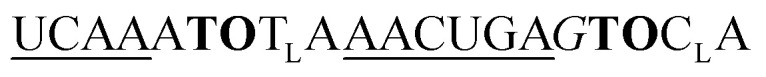	525	366	6.0	16	1.7	2.8	56.9	37.1
UE-TO-OMe-C1	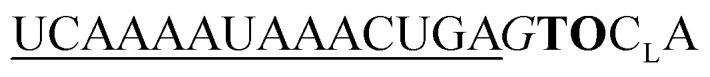	282	212	3.4	16	1.1	2.0	58.4	23.0
ED-QB^2^-OMe-1	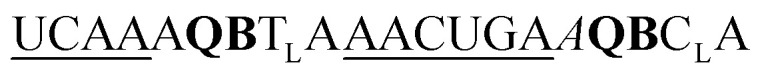	211	126	43	59	1.9	4.5	50.7	20.5
ED-QB-OMe-C1	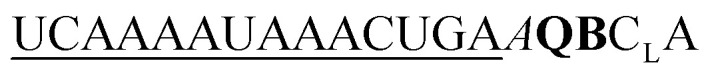	136	85	29	62	2.8	4.0	56.3	13.9
ED-TO^2^-OMe-1	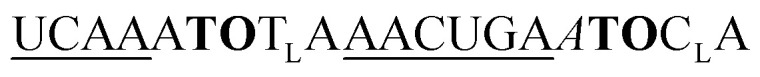	449	270	4	6.5	2.0	3.0	52.3	46.6
ED-TO-OMe-C1	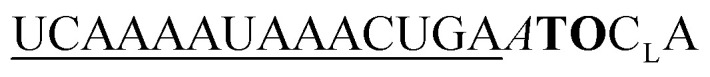	234	157	3	12	2.9	3.3	56.1	18.2

a Fluorescence intensity after hybridization with matched RNA target.

b Fluorescence enhancement at *λ*_em_ = 605 nm (QB probes) or *λ*_em_ = 535 nm (TO probes) upon matched hybridization where *F* and *F*_0_ is fluorescence intensity after and before hybridization, respectively.

c Specificity, see Fig. 2. Conditions: 0.5 μM FIT probe, 2.5 μM target RNA (if added) in buffer (100 mM NaCl, 10 mM Na_2_HPO_4_, pH 7), 37 °C, *λ*_e*x*_ = 560 nm (QB probes) or 485 nm (TO probes).

d Melting temperature *T*_M_ determined for duplexes formed upon mixing 1 μM probe with 1 μM matched RNA target in PBS buffer.

e Brightness (see Fig. 2) was determined at 25 °C. Underlined = 2′OMe-RNA; subscript L = LNA; bold letters = dye nucleotides; italicized letters = base opposite to editing site.

From this set of probes we concluded: dual QB labelling can (i) increase the fluorescence turn-on by introducing additional quench options for single or molten strands, (ii) enhance the intensity of fluorescence in the double strand while (iii) providing single mismatch discrimination.

Encouraged by the findings, we constructed FIT^2^ probes with 2′-OMe modifications, which improve resistance to nucleases and, therefore, facilitate measurements in cells and cell lysates. According to previously defined design criteria, the 2′-OMe RNA probes included a locked nucleic acid (LNA) building block as a 3′ neighbour of each Ser(QB) or Ser(TO) nucleotide and 2′-deoxynucleotides on each side of the serinol-LNA dinucleotides. This design confers high brightness to probe-target duplexes while keeping fluorescence of single strands low. Both QB and TO dyes were introduced at positions used for UE-QB^2^-3. In addition, we evaluated probes specific for the edited state of GlyR mRNA.

The dual labelled FIT^2^ probes (UE-Dye^2^-OMe-1 and ED-Dye^2^-OMe-1; dye = QB or TO) were compared with the corresponding mono labelled FIT probes (UE-Dye-OMe-C1 and ED-Dye-OMe-C1). Fluorescence measurements performed at 25 °C (see Fig. 3E and F) and 37 °C before and after addition of target revealed that the dual dye UE-QB^2^-OMe-1 probe provided a higher fluorescence turn-on (*F*/*F*_0_ = 55 and 125 at 25 °C and 37 °C, respectively) than the mono probe UE-QB-OMe-C1 (*F*/*F*_0_ = 23 and 85). In addition, the single nucleotide specificity was higher (37 °C: *S* = 13 for dual dye *vs. S* = 1.5 for mono dye probe) and the dual dye probe fluoresced with higher intensity.

The TO dye was placed in the same context and again single nucleotide specificity and brightness of fluorescence provided by dual TO probe (UE-TO^2^-OMe-1) was higher than for the mono dye probe (UE-TO-OMe-C1). A similar picture emerged for probes targeting the edited GlyR mRNA state. In this sequence context, introduction of the second QB can, again, improve the responsiveness, specificity and brightness of signalling, whereas the positive effects of the second TO were limited to brightness.

**Fig. 4:**
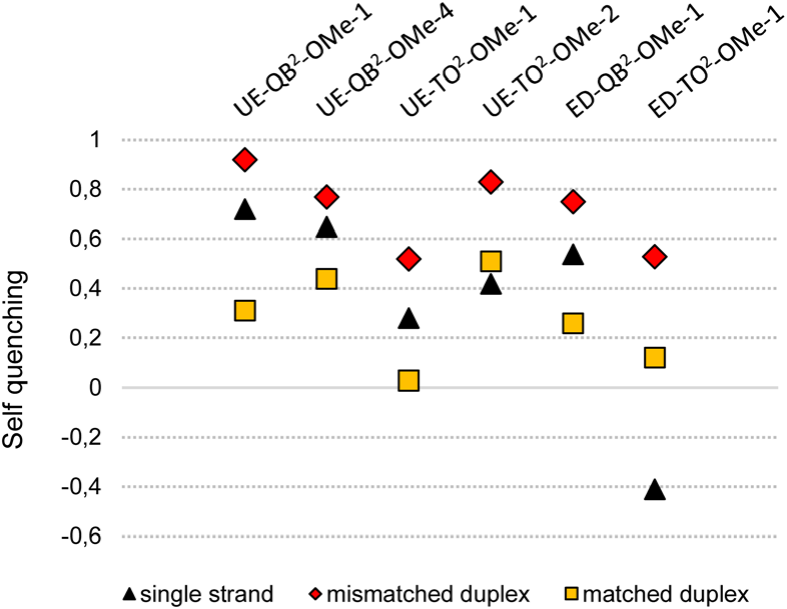
Self-quenching (*Q*) in FIT^2^ probes in single strands and after hybridization with RNA target. *Q* = 1 − (*F*(dye^2^-probe))/(*F*(dye^1^-probe) + *F*(dye^2^-probe)) with *F*(dye^2^) is the emission intensity of the FIT^2^ probe and *F*(dye^1^) or *F*(dye^2^) is the intensity of the corresponding mono dye FIT probes at 535 nm (*λ*_ex_ = 485 nm) for TO or 605 nm for QB (*λ*_ex_ = 560 nm). Conditions: see caption to Fig. 3. For details on sequences and spectral properties, see ESI.†

To expose the hybridization state dependency of dye–dye interactions in FIT^2^ probes, we determined the self-quenching efficiencies for single strands and target-bound probes in matched and single base mismatched form (Fig. 4). This involved the synthesis and evaluation of FIT probes containing a single TO or QB nucleotide at positions defined by the FIT^2^ probes (Table S1†). In this analysis, we included the four double FIT probes described above (UE-Dye^2^-OMe-1 and ED-Dye^2^-OMe-1; dye = QB or TO) and two additional probes (UE-QB^2^-OMe-4 and UE-TO^2^-OMe-2) used for detection of target in cell lysate (*vide infra*). Single nucleotide mismatched duplexes were characterized by efficient self-quenching occurring with 53–85% efficiency meaning that emission intensities of the FIT^2^ probes corresponded to only 15–47% of the intensity expected for the sum of intensities from the two mono dye probes. FIT^2^ probes engaged in matched hybridization experienced only 3–53% self-quenching.

Self-quenching is, amongst other effects, the result of excitation energy homotransfer. This transfer depends on the orientation of transition dipole moments. In single nucleotide mismatched duplexes one of the dyes gains flexibility which facilitates arrangements allowing for efficient transfer of excitation energy. According to an alternative interpretation, the high quenching efficiencies may be a consequence of delocalized excited states emerging from dye–dye interactions. As reported for multifluorophore-DNA such states may be particularly vulnerable to quenching.^45^ Regardless of the mechanism involved, self-quenching was, in each case, more efficient in mismatched duplexes than in the corresponding matched duplexes. The single-stranded probes showed a less uniform behaviour with self-quenching ranging between 67% for UE-QB^2^-OMe-4 to −40% for ED-TO^2^-OMe-1. Single strands can adopt conformations that enable dye–dye contact. In cases such as in ED-TO^2^-OMe-1 such a contact may decrease the degrees of freedom for rotations around the dye's methine bridge explaining why the single-strand can fluoresce with higher intensity (leading to “negative” self-quenching) than expected for the sum of the component intensities. While in most other cases turn-on with FIT^2^ probes is higher than with mono dye probes due to additional quench options in the single strand, the data indicates sequence dependencies.

**Fig. 5:**
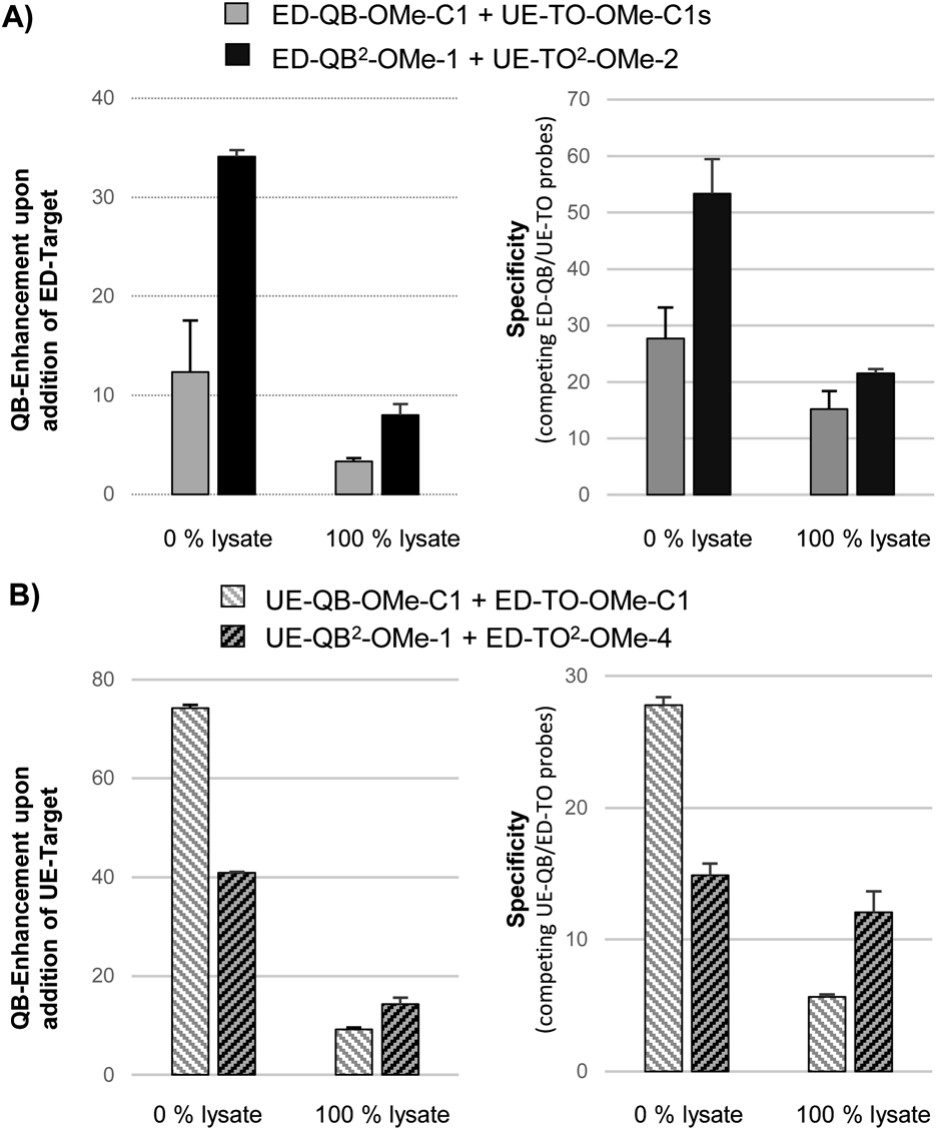
Fluorescence turn-on and single nucleotide specificity provided by competing probes with QB fluorescence reporting on (A) edited or (B) unedited state of GlyR mRNA in buffer (0% lysate) and 100% lysate of HEK293 cells. Specificity in (A) is (*F*_QB_(ED)/*F*_TO_(ED))/(*F*_QB_(UE)/*F*_TO_(UE)) and in (B) is (*F*_QB_(UE)/*F*_TO_(UE))/(*F*_QB_(ED)/*F*_TO_(ED)) with *F*_QB_(ED) and *F*_TO_(ED) as fluorescence emission from QB and TO, respectively, in the presence of edited RNA target, or *F*_QB_(UE) and *F*_TO_(UE) in the presence of unedited RNA target. Conditions: 500 nM of both QB and TO probes in PBS or lysate from HEK293 cells, 500 nM RNA (5′-CUGAAGGACUCACCCUGCYUCAGUUUAUUUUGA-3′; UE-RNA: Y = C, ED-RNA: Y = U), 37 °C, QB signal measured at 605 nm (*λ*_ex_ = 560 nm), TO signal at 535 nm (*λ*_ex_ = 485 nm).

Next, we compared mono dye FIT probes and dual dye FIT^2^ probes in a challenging matrix; 100% lysate of HEK293 cells which contains the multitude of a cell's molecules including RNA. Competing probes, one for detection of the edited state of GlyR mRNA *via* QB signalling and the other for detection of the unedited state *via* TO signalling, were equipped either with QB or with TO nucleotides. Out of four tested ED-QB^2^-OMe probes (Table S5†), we selected ED-QB^2^-OMe-1 as the brightest probe in this series. The selection of UE-TO^2^-OMe-2 (out of 4 tested probes (Table S4†)) as a competing probe was guided by *T*_M_ matching (*T*_M_ of mismatched TO probe duplexes should be lower than the *T*_M_ of matched QB probe duplexes) and single nucleotide specificity. The ED-QB^2^-OMe-1/UE-TO^2^-OMe-2 probe pair was compared with the corresponding mono dye probes, ED-QB-OMe-C1/UE-TO-OMe-C1s. Both probe sets were pipetted to lysates and RNA target was added. In addition, measurements were performed in PBS buffer without lysate. In buffer, the FIT^2^ probe system provided a 34-fold enhancement of QB signalling upon addition of edited target compared to a 13-fold by the mono dye pair (Fig. 5A, left). Lysate is a more complex matrix. Here the responsiveness of the mono dye FIT probe system decreased to a 4-fold enhancement of QB emission. With an 8-fold change the FIT^2^ probes provided a higher response also in this matrix. To assess the single nucleotide specificity, we determined QB/TO signal ratios in the presence of edited and unedited RNA (Tables S7 and S8†). For the FIT^2^ probe system in 100% PBS buffer, the presence of edited RNA afforded a 53-fold higher QB/TO ratio than unedited RNA (Fig. 5A, right). The specificity remained high (21-fold) also in 100% lysate. A much lower specificity (23-fold in buffer, 14-fold in 100% lysate) was obtained with mono dye probes.

In control experiments, we used QB probes for detection of the unedited state of GlyR mRNA. The probe UE-QB^2^-OMe-1 has the QB nucleotides in positions used also in ED-QB^2^-OMe-1. *T*_M_ matching and high sequence specificity recommended the use of ED-TO^2^-OMe-4 (out of 4 tested probes, Table S6†) as a competing partner specific for the edited state. For comparison, the sequence-analogous UE-QB-OMe-C1/ED-TO-OMe-C1 pair was included. In 100% buffer, the mono dye probe set furnished a higher QB response than the FIT^2^ probe set (Fig. 5B, left). However, the dual dye probe pair UE-QB^2^-OMe-1/ED-TO^2^-OMe-4 was superior in lysate. Also, the single nucleotide specificity assessed by QB/TO signal ratios was, again, higher with the FIT^2^ probe set than with the mono dye probes in both buffer and lysate (Fig. 5B, right).

Multiple labelling is a commonly used approach to increase brightness. Applied to oligonucleotides, it has been shown that the spacing between individual dyes should be sufficiently large to minimize self-quenching.^37,46–49^ Herein we have presented a dual labelling strategy that improves brightness and considers homo-FRET an advantage, providing options to increase the fluorogenicity of FIT probes without compromising specificity. Our data suggests that self-quenching can be a means to increase the specificity when FIT probes are applied in challenging matrices such as the lysate of HEK293 cells. In this regard, it is instructive to examine data from hybridization experiments, in which one of two base surrogates remains in a single stranded sequence overhang (Fig. S12†). Fluorescence remained low despite partial hybridization, probably because the dye acts a quencher within the ‘non-hybridized’ region. This property may contribute to the increased specificity observed for fluorescence signalling from FIT^2^ probes in cell lysate.

## Conclusion

In our pursuit of approaches providing RNA FIT hybridization probes with enhanced brightness, responsiveness and single nucleotide specificity of fluorescence signalling, we have explored FIT^2^ probes that contain two identical fluorescent base surrogates. In most cases, single strand fluorescence of FIT^2^ probes was lower than that of mono dye FIT probes, most likely because single strands provide options for self-quenching *via* dye–dye interactions. Typically, dual dye FIT^2^ probes are brighter than mono dye probes when dye nucleotides are positioned in 8–10 nt distance. Our experiments were aiming for the development of a probe system that enables the detection of the C → U editing state of a RNA target with a sequence found in mRNA encoding for the Glycine Receptor GlyR. Our data revealed a surprisingly high degree of self-quenching for FIT^2^ probe-RNA duplexes containing a single nucleotide mismatch next to one of the fluorescent base surrogates (thiazole orange, TO or quinoline blue, QB). This and self-quenching in the single stranded state probably contributes to the high specificity observed when differently coloured FIT^2^ probes were used as pairs for detection of RNA in a complex matrix such as cell lysate. Our experiments showed that responsiveness and single nucleotide specificity are affected when FIT probes are used in cell lysate. In this regard, the FIT^2^ approach is an easy-to-implement method to improve specificity as well as brightness and responsiveness of fluorescence.

## Data availability

The datasets supporting this article have been uploaded as part of the ESI.†

## Author contributions

S. S. and A. H. synthesized oligonucleotide probes. S. S. and A. K. evaluated properties of oligonucleotide probes. S. S. and O. S. analyzed the data. S. S. wrote the first draft of the manuscript. O. S. conceived the research and wrote the final version of the manuscript.

## Conflicts of interest

There are no conflicts to declare.

## Supplementary Material

SC-014-D3SC00363A-s001

## References

[cit1] Buxbaum A. R., Haimovich G., Singer R. H. (2015). In the right place at the right time: Visualizing and understanding mRNA localization. Nat. Rev. Mol. Cell Biol..

[cit2] Faltin B., Zengerle R., Vonstetten F. (2013). Current methods for fluorescence-based universal sequence-dependent detection of nucleic acids in homogenous assays and clinical applications. Clin. Chem..

[cit3] Tomoike F., Abe H. (2019). RNA imaging by chemical probes. Adv. Drug Delivery Rev..

[cit4] Tyagi S., Kramer F. R. (1996). Molecular beacons – probes that fluoresce upon hybridization. Nat. Biotechnol..

[cit5] Asanuma H., Akahane M., Niwa R., Kashida H., Kamiya Y. (2015). Highly Sensitive and Robust Linear Probe for Detection of mRNA in Cells. Angew. Chem., Int. Ed..

[cit6] Okamoto A. (2011). ECHO probes: a concept of fluorescence control for practical nucleic acid sensing. Chem. Soc. Rev..

[cit7] Holzhauser C., Wagenknecht H. A. (2011). Stem-Labeled Molecular Beacons for Distinct Fluorescent Color Readout. Angew. Chem., Int. Ed..

[cit8] Johansson M. K., Cook R. M. (2003). Intramolecular dimers: A new design strategy for fluorescence-quenched probes. Chem.–Eur. J..

[cit9] Tyagi S., Marras S. A. E., Kramer F. R. (2000). Wavelength-shifting molecular beacons. Nat. Biotechnol..

[cit10] Socher E., Bethge L., Knoll A., Jungnick N., Herrmann A., Seitz O. (2008). Low-noise stemless PNA beacons for sensitive DNA and RNA detection. Angew. Chem., Int. Ed..

[cit11] Socher E., Knoll A., Seitz O. (2012). Dual fluorophore PNA FIT-probes – Extremely responsive and bright hybridization probes for the sensitive detection of DNA and RNA. Org. Biomol. Chem..

[cit12] Seitz O., Bergmann F., Heindl D. (1999). A convergent strategy for the modification of peptide nucleic acids: novel mismatch-specific PNA-hybridization probes. Angew. Chem., Int. Ed..

[cit13] Köhler O., Seitz O. (2003). Thiazole orange as fluorescent universal base in peptide nucleic acids. Chem. Commun..

[cit14] Köhler O., Jarikote D. V., Seitz O. (2005). Forced intercalation probes (FIT probes): thiazole orange as a fluorescent base in peptide nucleic acids for homogeneous single-nucleotide-polymorphism detection. ChemBioChem.

[cit15] Bethge L., Singh I., Seitz O. (2010). Designed thiazole orange nucleotides for the synthesis of single labelled oligonucleotides that fluoresce upon matched hybridization. Org. Biomol. Chem..

[cit16] Hövelmann F., Bethge L., Seitz O. (2012). Single Labeled DNA FIT Probes for Avoiding False-Positive Signaling in the Detection of DNA/RNA in qPCR or Cell Media. ChemBioChem.

[cit17] Hövelmann F., Seitz O. (2016). DNA Stains as Surrogate Nucleobases in Fluorogenic Hybridization Probes. Acc. Chem. Res..

[cit18] Isacsson J., Cao H., Ohlsson L., Nordgren S., Svanvik N., Westman G., Kubista M., Sjoback R., Sehlstedt U. (2000). Rapid and specific detection of PCR products using light-up probes. Mol. Cell. Probes.

[cit19] Karunakaran V., Lustres J. L. F., Zhao L. J., Ernsting N. P., Seitz O. (2006). Large dynamic stokes shift of DNA intercalation dye thiazole orange has contribution from a high-frequency mode. J. Am. Chem. Soc..

[cit20] Jarikote D. V., Krebs N., Tannert S., Röder B., Seitz O. (2007). Exploring base-pair-specific optical properties of the DNA stain thiazole orange. Chem.–Eur. J..

[cit21] Socher E., Jarikote D. V., Knoll A., Röglin L., Burmeister J., Seitz O. (2008). FIT probes: Peptide nucleic acid probes with a fluorescent base surrogate enable real-time DNA quantification and single nucleotide polymorphism discovery. Anal. Biochem..

[cit22] Swenson C. S., Argueta-Gonzalez H. S., Sterling S. A., Robichaux R., Knutson S. D., Heemstra J. M. (2023). Forced Intercalation Peptide Nucleic Acid Probes for the Detection of an Adenosine-to-Inosine Modification. ACS Omega.

[cit23] Kummer S., Knoll A., Socher E., Bethge L., Herrmann A., Seitz O. (2011). Fluorescence Imaging of Influenza H1N1 mRNA in Living Infected Cells Using Single-Chromophore FIT-PNA. Angew. Chem., Int. Ed..

[cit24] Kummer S., Knoll A., Socher E., Bethge L., Herrmann A., Seitz O. (2012). PNA FIT-Probes for the Dual Color Imaging of Two Viral mRNA Targets in Influenza H1N1 Infected Live Cells. Bioconjugate Chem..

[cit25] Hövelmann F., Gaspar I., Loibl S., Ermilov E. A., Röder B., Wengel J., Ephrussi A., Seitz O. (2014). Brightness through Local Constraint-LNA-Enhanced FIT Hybridization Probes for *In Vivo* Ribonucleotide Particle Tracking. Angew. Chem., Int. Ed..

[cit26] Hövelmann F., Gaspar I., Chamiolo J., Kasper M., Steffen J., Ephrussi A., Seitz O. (2016). LNA-enhanced DNA FIT-probes for multicolour RNA imaging. Chem. Sci..

[cit27] Kam Y., Rubinstein A., Nissan A., Halle D., Yavin E. (2012). Detection of Endogenous K-ras mRNA in Living Cells at a Single Base Resolution by a PNA Molecular Beacon. Mol. Pharm..

[cit28] Kolevzon N., Hashoul D., Naik S., Rubinstein A., Yavin E. (2016). Single point mutation detection in living cancer cells by far-red emitting PNA-FIT probes. Chem. Commun..

[cit29] Sonar M. V., Wampole M. E., Jin Y. Y., Chen C. P., Thakur M. L., Wickstrom E. (2014). Fluorescence Detection of KRAS2 mRNA Hybridization in Lung Cancer Cells with PNA-Peptides Containing an Internal Thiazole Orange. Bioconjugate Chem..

[cit30] Torres A. G., Fabani M. M., Vigorito E., Williams D., Al-Obaidi N., Wojciechowski F., Hudson R. H. E., Seitz O., Gait M. J. (2012). Chemical structure requirements and cellular targeting of microRNA-122 by peptide nucleic acids anti-miRs. Nucleic Acids Res..

[cit31] Sato T., Sato Y., Nishizawa S. (2016). Triplex-Forming Peptide Nucleic Acid Probe Having Thiazole Orange as a Base Surrogate for Fluorescence Sensing of Double-stranded RNA. J. Am. Chem. Soc..

[cit32] Sato T., Sato Y., Nishizawa S. (2017). Optimization of the Alkyl Linker of TO Base Surrogate in Triplex-Forming PNA for Enhanced Binding to Double-Stranded RNA. Chem.–Eur. J..

[cit33] Chiba T., Sato T., Sato Y., Nishizawa S. (2017). Red-emissive triplex-forming PNA probes carrying cyanine base surrogates for fluorescence sensing of double-stranded RNA. Org. Biomol. Chem..

[cit34] Samanta D., Ebrahimi S. B., Kusmierz C. D., Cheng H. F., Mirkin C. A. (2020). Protein Spherical Nucleic Acids for Live-Cell Chemical Analysis. J. Am. Chem. Soc..

[cit35] Hövelmann F., Gaspar I., Ephrussi A., Seitz O. (2013). Brightness Enhanced DNA FIT-Probes for Wash-Free RNA Imaging in Tissue. J. Am. Chem. Soc..

[cit36] Fang G. M., Chamiolo J., Kankowski S., Hövelmann F., Friedrich D., Löwer A., Meier J. C., Seitz O. (2018). A bright FIT-PNA hybridization probe for the hybridization state specific analysis of a C → U RNA edit: *Via* FRET in a binary system. Chem. Sci..

[cit37] Kato T., Kashida H., Kishida H., Yada H., Okamoto H., Asanuma H. (2013). Development of a robust model system of FRET using base surrogates tethering fluorophores for strict control of their position and orientation within DNA duplex. J. Am. Chem. Soc..

[cit38] Meier J. C., Henneberger C., Melnick I., Racca C., Harvey R. J., Heinemann U., Schmieden V., Grantyn R. (2005). RNA editing produces glycine receptor α3P185L, resulting in high agonist potency. Nat. Neurosci..

[cit39] Eisfeld A., Briggs J. S. (2006). The J- and H-bands of organic dye aggregates. Chem. Phys..

[cit40] Hestand N. J., Spano F. C. (2018). Expanded Theory of H- and J-Molecular Aggregates: The Effects of Vibronic Coupling and Intermolecular Charge Transfer. Chem. Rev..

[cit41] Berndl S., Wagenknecht H. A. (2009). Fluorescent Color Readout of DNA Hybridization with Thiazole Orange as an Artificial DNA Base. Angew. Chem., Int. Ed..

[cit42] Ikeda S., Kubota T., Yuki M., Okamoto A. (2009). Exciton-Controlled Hybridization-Sensitive Fluorescent Probes: Multicolor Detection of Nucleic Acids. Angew. Chem., Int. Ed..

[cit43] Rye H. S., Yue S., Wemmer D. E., Quesada M. A., Haugland R. P., Mathies R. A., Glazer A. N. (1992). Stable Fluorescent Complexes of Double-Stranded DNA with Bis- Intercalating Asymmetric Cyanine Dyes - Properties and Applications. Nucleic Acids Res..

[cit44] Robertson K. L., Yu L., Armitage B. A., Lopez A. J., Peteanu L. A. (2006). Fluorescent PNA Probes as Hybridization Labels for Biological RNA. Biochemistry.

[cit45] Teo Y. N., Kool E. T. (2012). DNA-Multichromophore Systems. Chem. Rev..

[cit46] Randolph J. B., Waggoner A. S. (1997). Stability, specificity and fluorescence brightness of multiply-labeled fluorescent DNA probes. Nucleic Acids Res..

[cit47] Femino A. M., Fay F. S., Fogarty K., Singer R. H. (1998). Visualization of single RNA transcripts *in situ*. Science.

[cit48] Börjesson K., Preus S., El-Sagheer A. H., Brown T., Albinsson B., Wilhelmsson L. M. (2009). Nucleic Acid Base Analog FRET-Pair Facilitating Detailed Structural Measurements in Nucleic Acid Containing Systems. J. Am. Chem. Soc..

[cit49] Raj A., van den Bogaard P., Rifkin S. A., van Oudenaarden A., Tyagi S. (2008). Imaging individual mRNA molecules using multiple singly labeled probes. Nat. Methods.

