# Supplementary material for: Double FIT hybridization probes – towards enhancing brightness, turn-on and specificity of RNA detection
Source: Chem Sci. 2023 Mar 23;14(15):4166–73. doi: 10.1039/d3sc00363a (PMC10094420; doi:10.1039/d3sc00363a)
Supplement: SC-014-D3SC00363A-s001 [file SC-014-D3SC00363A-s001.pdf]

Electronic Supplementary Material (ESI) for Chemical Science.
This journal is © The Royal Society of Chemistry 2023

Inhalt

1. General Information 2
2. Characterization Data 3
3. Optical Properties and T_M data of FIT Probes 5
   Dual-dye FIT^2 Probes 5
4. Fluorescence and Absorption Spectra 10
   UE-QB^2 and UE-QB FIT probes 10
   UE-QB^2-OMe and UE-QB-OMe FIT probes 13
   UE-TO^2-OMe and UE-TO-OMe FIT probes 15
   ED-QB^2-OMe and ED-QB-OMe FIT probes 18
   ED-TO^2-OMe and ED-TO-OMe FIT probes 20
   Normalised Emission of Selected FIT Probes 22
5. Melting Curves 23
   UE-QB^2 and UE-QB FIT probes 23
   UE-QB^2-OMe and Controls 25
   UE-TO^2-OMe and UE-TO-OMe FIT probes 26
   ED-QB^2-OMe and ED-QB-OMe FIT probes 27
   ED-TO^2-OMe and ED-TO-OMe FIT probes 28
6. Competitive Hybridization in Cell Lysate 29
7. Partial Hybridization 31
8. MALDI-TOF MS Analysis 32
9. HPLC and UPLC Analysis 37
10. References 44

1. General Information

Materials: Chemicals were purchased from Sigma-Aldrich (St. Louis, USA), TCI Deutschland GmbH, Link Technologies (Bellshill, Scotland) or CarlRoth (Karlruhe, Germany). Synthetic oligo ribonucleotides were obtained from Biomers GmbH (Ulm, Germany). Aqueous solutions were made of water purified using a Milli-Pore purification device.

HPLC purification and analysis: Semi preparative and analytical HPLC were carried out on a Gilson 1105 HPLC System (Gilson, Limburg, Germany). A YMC-Triart C_{18} 150x100 column at a flow rate of 8 mL/min at 55 °C, linear gradient (gradient 1: 15-40% B; gradient 2: 5-40% B) was used for purification. The mobile phase consisted of a binary mixture of A (0.1 M triethylammonium acetate buffer, pH = 7.4, aq.) and B (acetonitrile). For analytical HPLC, a YMC C_{18} 250x4.6 column at a flow rate of 1.5 mL/min at 55 °C, 5-50% B in 10 min was used. Alternatively, probes were analysed on a ultra-high-performance chromatography (UPLC) system using Acquity UPLC Oligonucleotide BEH C_{18} Columns, (50/2.1 mm; 1.7 µM; 130Å) on a Aquity H-Class system (Waters, Milford, MA, USA) equipped with a QDa detector and a gradient 3-20%.

MALDI-TOF mass spectrometry: MALDI-TOF mass spectra were measured on a Shimadzu Axima Confidence spectrometer (Shimadzu, Kyoto, Japan) in positive mode. For measurements, HPA Matrix was used (1:1 mixture of 50 mg/mL 3-hydroxy picolinic acid in acetonitrile/H_2O, 1:1 and 50 mg/mL diamonium citrate in acetonitrile/H_2O, 1:1).

DNA FIT probe synthesis: DNA FIT probes were assembled by using a Bioautomation MerMade-4 (Irving, Texas). 3'-C3-Spacer CPG (1 µmol, 500 Å pore size), DNA phosphoramidites (dT, dG(DMF), dC(Bz), dA(Bz)) and 2'-O-Me-building blocks were purchased from Link (Biosearch Technologies, Hoddeston, UK). DNA synthesis reagents from Carl Roth and EMP-Biotech (Berlin, Germany). LNA phosphoramidites were obtained from Exiqon (Vedbak, Denmark). All phosphoramidites were used according to manufacturer's instructions. The synthesis of QB and TO serinol building blocks was described elsewhere.[1] The quality of each coupling step was monitored by measuring the absorbance of DMT cleavage solutions. The synthesis was programmed to yield oligomers carrying the terminal DMT protective group „DMT-on". After synthesis, the resulting CPGs were dried under reduced pressure and transferred to 2 mL tubes. 1 mL of aqueous ammonia (32%) was added and the tubes were agitated for 2 h at 55 °C. The volatiles were removed, and the remaining resin was filtered off. The crude product was purified by RP-HPLC (gradient 1). DMT cleavage was carried out using 300 µL of 80% aqueous AcOH for 30 min at room temperature. The oligonucleotides were precipitated with iPrOH and ammonium acetate and purified by RP-HPLC again (gradient 2). Purified DNA FIT probes were desalted by precipitation from 0.1 volume sodium acetate buffer (3 M, pH 5.4) and 1 volume isopropanol, washed with 70 % (v/v) ethanol, dried and dissolved in ultrapure water. Products were characterized by MALDI-TOF mass spectrometry and RP-HPLC analysis.

Fluorescence spectroscopy: Fluorescence emission and excitation spectra were measured by using a Varian Cary Eclipse fluorescence spectrometer (Agilent Technologies, Santa Clara, CA, USA). Clear quartz cuvettes (10 mm, 1.4 ml) were filled with phosphate buffer (100 mM NaCl, 10 mM Na_2HPO_4, pH 7) and buffer fluorescence corrected. FIT probes and target RNA were added as specified. and, after measuring a blank, FIT probes were added as specified. Probe concentration was verified by determining its absorption at 260 nm in the same cuvette on an UV/VIS spectrometer (*Jasco*, Tokio, Japan). The relative deviation from the targeted concentration was later used as a correction factor for the obtained fluorescence intensities. Fluorescence spectra were recorded at 25 or 37 °C. Prior to each measurement, samples were allowed to equilibrate for 5 min to the specified temperature. To determine the spectroscopic properties of the double strand, RNA target was added to the cuvette and the mixture resuspended thoroughly. For proper hybridization, the cuvette was briefly heated up

to 90 °C and cooled down again prior the measurement. The spectra are the average of three measurement cycles. Quantum yields were assessed by using ATTO 590 (ATTO-Tec GmbH, Siegen, Germany) as standard. The fluorescence measurements were reproducible within 5-10% error.

UV-Vis spectroscopy: UV-Vis absorption of the probes was measured on a V-750 spectrophotometer equipped with a PAC-743R Peltier cell changer (*Jasco*, Tokio, Japan) and connected to a F250 recirculating cooler (*Julabo*, Seelbach, Germany).

For concentration determination, an absorption spectrum (800–220 nm, 1 nm steps) of the respective solution was recorded. Probe concentration was calculated from the absorption at 260 nm according to the Beer-Lambert law. The molar extinction coefficients of the probes were calculated with the OligoAnalyzer (*Integrated DNA Technologies*, Coralville, IA, USA). QB and TO base surrogates were treated as adenosine.

For melting temperature experiments, 1 µM probe and 1 µM synthetic RNA target were diluted in 1 ml phosphate buffer (100 mM NaCl, 10 mM Na_2HPO_4, pH 7.0). Absorption (λ = 260 nm) was determined between 20 °C and 90 °C with a heating rate of 1.0 °C/min and a sampling rate of 5 data points/°C. For each experiment three measurements were averaged, and the T_m was calculated as the maximum of the first derivative of a sigmoidal fit (for curves see Figures S7-S11). Curve fitting was performed with Origin 2019 (*OriginLab*, Northhampton, MA, USA).

2. Characterization Data

Table S1 Characterization data for FIT and FIT^2 probes

FIT-Probe	Sequence 5' – 3'	MALDI-TOF-MS calc. $[M + H]^+$	MALDI-TOF-MS found $[M + H]^+$	r_t [min]
UE-QB^2-1	AAA**QB**T_LAAACTGAG**QB**C_LA-C3	5438.9	5440.4	8.97[a]
UE-QB^2-2	CAAA**QB**T_LAAACTGAG**QB**C_LA-C3	5742.2	5742.5	8.81[a]
UE-QB^2-3	TCAAA**QB**T_LAAACTGAG**QB**C_LA-C3	6046.2	6046.8	8.86[a]
UE-QB^2-4	AAAAT**QB**A_LACTGAG**QB**C_LA-C3	5451.0	5452.6	7.48[a]
UE-QB^2-5	CAAAAT**QB**A_LACTGAG**QB**C_LA-C3	5742.2	5747.0	7.16[a]
UE-QB^2-6	TCAAAAT**QB**A_LACTGAG**QB**C_LA-C3	6046.4	6050.4	7.39[a]
UE-QB-C1	TCAAAAT_LAAACTGAG**QB**C_LA-C3	5854.1	5851.8	2.72[b]
UE-QB-C2	TCAAA**QB**T_LAAACTGAGGC_LA-C3	5854.1	5856.4	2.81[b]
UE-QB-C3	TCAAAAT**QB**A_LACTGAGGC_LA-C3	5854.1	5852.9	2.81[b]
UE-QB^2-OMe-1	UCAAA**QB**T_LAAACUGAG**QB**C_LA-C3	6318.4	6321.6	8.97[a]
UE-QB^2-OMe-2	AAAAT**QB**A_LACUGAG**QB**C_LA-C3	5679.2	5680.3	7.94[a]
UE-QB^2-OMe-3	CAAAAT**QB**A_LACUGAG**QB**C_LA-C3	5998.2	5999.2	7.75[a]

UE-QB^2-OMe-4	UCAAAAU**QB**A$_L$ACUGAG**QB**C$_L$A-C3	6318.4	6317.3	8.97[a]
UE-QB-OMe-C1	UCAAAAUAAACUGAG**QB**C$_L$A-C3	6232.3	6230.8	3.15[b]
UE-QB-OMe-C2	UCAAA**QB**T$_L$AAACUGAGGCA-C3	6248.3	6244.4	3.16[b]
UE-QB-OMe-C3	UCAAAAT**QB**A$_L$ACUGAGGCA-C3	6248.3	6248.5	3.20[b]
ED-TO^2-OMe-1	UCAAAT**TO**T$_L$AAACUGAAT**O**C$_L$A-C3	6314.5	6312.5	8.96[a]
ED-TO^2-OMe-2	AAAAT**TO**A$_L$ACUGAAT**O**C$_L$A-C3	5675.1	5672.4	9.11[a]
ED-TO^2-OMe-3	CAAAAT**TO**A$_L$ACUGAAT**O**C$_L$A-C3	5994.3	5991.1	8.93[a]
ED-TO^2-OMe-4	UCAAAAT**TO**A$_L$ACUGAAT**O**C$_L$A-C3	6314.5	6312.2	9.00[a]
ED-TO-OMe-C1	UCAAAAUAAACUGAAT**O**C$_L$A-C3	6222.3	6221.4	3.15[b]
ED-TO-OMe-C2	UCAAAT**O**T$_L$AAACUGAAGCA-C3	6238.3	6236.7	3.11[b]
ED-TO-OMe-C3	UCAAAAT**TO**A$_L$ACUGAAGCA-C3	6238.42	6238.5	3.33[b]
UE-TO^2-OMe-1	UCAAAT**O**T$_L$AAACUGAGT**O**C$_L$A-C3	6330.5	6326.8	8.77[a]
UE-TO^2-OMe-2	AAAAT**TO**A$_L$ACUGAGT**O**C$_L$A-C3	5691.1	5694.7	8.95[a]
UE-TO^2-OMe-3	CAAAAT**TO**A$_L$ACUGAGT**O**C$_L$A-C3	6010.30	6013.1	8.83[a]
UE-TO^2-OMe-4	UCAAAAT**TO**A$_L$ACUGAGT**O**C$_L$A-C3	6300.5	6300.0	3.25[b]
UE-TO-OMe-C1	UCAAAAUAAACUGAGT**O**C$_L$A-C3	6238.40	6239.1	3.11[b]
UE-TO-OMe-C1s	AAAAUAAACUGAGT**O**C$_L$A-C3	5598.0	5599.5	2.2[a]
UE-TO-OMe-C2	UCAAAT**O**T$_L$AAACUGAGGCA-C3	6254.3	6254.7	3.09[b]
UE-TO-OMe-C3	UCAAAAT**TO**A$_L$ACUGAGGCA-C3	6254.4	6253.3	3.12[b]
ED-QB^2-OMe-1	UCAAA**QB**T$_L$AAACUGAA**QB**C$_L$A-C3	6302.4	6300.7	7.95[a]
ED-QB^2-OMe-2	AAAAT**QB**A$_L$ACUGAA**QB**C$_L$A-C3	5663.0	5665.6	8.02[a]
ED-QB^2-OMe-3	CAAAAT**QB**A$_L$ACUGAA**QB**C$_L$A-C3	5982.4	5981.3	7.95[a]
ED-QB^2-OMe-4	UCAAAAT**QB**A$_L$ACUGAA**QB**C$_L$A-C3	6302.6	6300.0	3.13[b]
ED-QB-OMe-C1	UCAAAAUAAACUGAA**QB**C$_L$A-C3	6216.4	6214.3	3.22[b]
ED-QB-OMe-C2	UCAAA**QB**T$_L$AAACUGAAGCA-C3	6232.3	6232.0	3.20[b]
ED-QB-OMe-C3	UCAAAAT**QB**A$_L$ACUGAAGCA-C3	6232.3	6232.0	3.21[b]

[a] retention time observed for analysis on a Triart C_{18} column (4.6 x 250 mm, 5 µm, 120 Å; *YMC Europe*) was used, operated at 55 °C with a flow rate of 1.5 mL/min and a linear gradient of 5-50 % B in 10 min. [b] retention time observed for analysis on a Aquity H-Class (Waters, Milford, MA, USA) ultra-high-performance chromatography (UPLC) system using an Acquity UPLC Oligonucleotide BEH C_{18} Column (2.1 x 50 mm; 1.7 µm; 130 Å; *Waters Corporation*), operated at 70 °C with a flow rate of 0.4 ml/min and a linear gradient of 3-20 % B in 7 min.

3. Optical Properties and T_M data of FIT Probes

Dual-dye FIT^2 Probes

Table S2 Optical properties and T_M data for UE-QB^2 and UE-QB FIT probes.

	Sequence 5' – 3'	RNA target	F_0 25 °C	F_0 37 °C	F 25 °C	F 37 °C	ϕ	ϕ/ϕ₀	Br	T_m
UE-QB^2										
1	AAA**QB**T$_L$AAACTGA**GQB**C$_L$A	m	2.5	1.4	157.0	92.3	0.18	45.3	13.5	49.9
		mm	2.8	1.2	24.7	3.1	0.03	5.6	3.2	50.9
2	CAAA**QB**T$_L$AAACTGA**GQB**C$_L$A	m	2.5	1.0	173.6	110.3	0.15	45.0	13.1	51.2
		mm	2.1	1.1	25.7	4.2	0.03	6.7	2.8	48.5
3	TCAAA**QB**T$_L$AAACTGA**GQB**C$_L$A	m	1.9	0.9	176.0	116.1	0.15	53.8	12.3	52.0
		mm	2.0	1.0	25.9	4.3	0.03	6.7	2.7	48.7
4	AAAAT**QB**A$_L$ACTGA**GQB**C$_L$A	m	1.4	0.8	138.8	66.1	0.11	45.8	8.9	49.6
		mm	1.2	0.8	7.7	1.1	0.01	2.4	0.7	50.9
5	CAAAAT**QB**A$_L$ACTGA**GQB**C$_L$A	m	1.3	0.7	157.0	81.5	0.12	54.6	10.3	48.3
		mm	1.0	0.4	13.7	1.0	0.01	4.2	1.4	50.6
6	TCAAAAT**QB**A$_L$ACTGA**GQB**C$_L$A	m	1.3	0.7	157.4	83.8	0.13	54.2	11.0	49.2
		mm	1.1	0.6	12.3	1.5	0.01	3.7	1.0	51.1
UE-QB										
C1	TCAAAATAAACTGA**GQB**C$_L$A	m	3.3	1.4	151.9	98.9	0.32	34.7	12.7	48.4
		mm	3.9	1.8	11.2	2.2	0.03	2.8	1.0	53.3
C2	TCAAA**QB**T$_L$AAACTGA**G**GCA	m	3.6	1.9	78.7	49.4	0.19	16.6	7.4	49.8
		mm	3.8	1.9	29.9	5.5	0.07	6.4	2.8	49.0
C3	TCAAAAT**QB**A$_L$ACTGA**G**GCA	m	2.3	1.3	110.2	61.2	0.30	37.3	10.3	49.2
		mm	2.7	1.4	30.4	3.4	0.08	9.4	3.2	51.1

Conditions: 0.5 µM probe was measured at 25 °C or 37 °C before (F_0) and after (F) addition of 5 eq. RNA target (match / mismatch: 5'-CUGAAGGACUCACCCUGC**Y**UCAGUUUAUUU UGA-3', UE: Y = C; ED: Y = U). Quantum yield ϕ and brightness Br = $\varepsilon_{\lambda ex} \cdot \phi_{ds}$ / 1000 in mL · mol^{-1} · cm^{-1} were determined at 25 °C. λ_{ex} = 560 nm, λ_{em} = 605 nm. Melting temperature T_m in °C was measured with 1 µM probe and 1 µM target RNA in PBS buffer (100 mM NaCl, 10 mM Na_2HPO_4, pH 7.0). Subscript L = LNA; bold letters = base surrogate. Match and mismatch RNA target are indicated with m and mm, respectively.

Table S3 Optical properties and T_M data for UE-QB^2-OMe and UE-QB-OMe FIT probes.

	Sequence 5' – 3'	RNA target	F_0		F		ϕ	ϕ/ϕ$_0$	Br	T_m
			25 °C	37 °C	25 °C	37 °C				
UE-QB^2-OMe										
1	UCAAA**QB**T$_L$AAACUGA**GQB**C$_L$A	m	4.4	1.3	242.9	156.3	0.38	41.6	29.4	49.6
		mm	4.1	1.6	54.4	13.7	0.09	1.5	7.0	49.6
2	AAAAT**QB**A$_L$ACUGA**GQB**C$_L$A	m	2.7	0.9	173.9	99.7	0.23	17.8	16.2	49.8
		mm	2.5	1.0	78.5	17.6	0.10	15.1	7.3	49.3
3	CAAAAT**QB**A$_L$ACUGA**GQB**C$_L$ A	m	3.3	1.0	194.3	119.0	0.24	39.9	16.8	48.3
		mm	3.7	1.0	121.1	41.0	0.15	9.4	11.2	48.6
4	UCAAAAT**QB**A$_L$ACUGA**GQB**C$_L$A	m	4.3	1.1	197.1	117.0	0.25	48.6	17.1	48.6
		mm	4.7	1.4	116.3	37.9	0.13	19.8	9.0	48.2
UE-QB-OMe										
C1	UCAAAAUAAACUGA**GQB**C$_L$A	m	9.7	1.8	215.9	164.4	0.58	69.9	23.1	62.1
		mm	10.3	1.8	201.3	133.4	0.53	16.9	21.2	53.2
C2	UCAAA**QB**T$_L$AAACUGAG**G**CA	m	6.0	3.0	128.3	85.2	0.30	13.3	9.9	66.5
		mm	6.3	2.6	120.1	81.7	0.26	11.0	9.2	59.6
C3	UCAAAAT**QB**A$_L$ACUGAG**G**CA	m	4.1	2.1	113.7	73.9	0.19	15.1	7.6	65.3
		mm	3.6	1.8	118.4	76.3	0.17	13.7	6.4	58.2

Conditions: 0.5 µM probe was measured at 25 °C or 37 °C before (F_0) and after (F) addition of 5 eq. RNA target (match / mismatch: 5'-CUGAAGGACUCACCCUGC**Y**UCAGUUUAUUU UGA-3', UE: Y = C; ED: Y = U). Quantum yield ϕ and brightness Br = $\varepsilon_{\lambda ex} \cdot \phi_{ds}$ / 1000 in mL · mol^{-1} · cm^{-1} were determined at 25 °C. λ_{ex} = 560 nm, λ_{em} = 605 nm. Melting temperature T_m in °C was measured with 1 µM probe and 1 µM target RNA in PBS buffer (100 mM NaCl, 10 mM Na_2HPO_4, pH 7.0). Underscored letters = 2'OMe-RNA; subscript L = LNA; bold letters = base surrogate. Match and mismatch RNA target are indicated with m and mm, respectively.

Table S4 Optical properties and T_M data for UE-TO^2-OMe and UE-TO-OMe FIT probes.

	Sequence 5' – 3'	RNA target	F_0		F		ϕ	ϕ/ ϕ$_0$	Br	T_m
			25 °C	37 °C	25 °C	37 °C				
UE-TO^2-OMe										
1	UCAAATOT$_L$AAACUGAGTOC$_L$A	m	88.2	22.6	525.2	363.5	0.37	5.6	37.1	56.9
		mm	86.9	22.2	310.2	128.7	0.25	4.1	25.2	50.2
2	AAAATTOA$_L$ACUGAGTOC$_L$A	m	32.4	15.5	242.6	159.0	0.22	5.5	22.2	52.5
		mm	33.3	15.7	115.8	38.1	0.11	2.5	11.8	51.3
3	CAAAATTOA$_L$ACUGAGTOC$_L$ A	m	30.2	14.4	233.5	145.8	0.24	5.7	23.8	54.4
		mm	31.4	14.8	150.2	55.8	0.16	3.6	15.9	50.2
4	UCAAAATTOA$_L$ACUGAGTOC$_L$A	m	29.5	13.6	223.2	134.4	0.23	5.5	22.3	47.6
		mm	28.9	13.2	134.1	13.2	0.14	3.5	13.8	42.1
UE-TO-OMe										
C1	UCAAAATAAACUGAGTOC$_L$A	m	66.9	16.2	339.1	245.9	0.59	4.5	23.0	58.4
		mm	69.1	17.0	219.4	131.3	0.32	2.8	14.8	48.2
C1s	AAAAUAAACUGAGTOC$_L$A	m	69.8	16.6	351.2	252.6	0.68	4.3	32.8	56.4
		mm	71.4	16.8	249.6	149.7	0.53	3.8	31.9	47.8
C2	UCAAATOT$_L$AAACUGAGGCA	m	33.3	17.9	280.6	196.7	0.43	7.5	21.2	63.5
		mm	32.4	18.8	281.9	193.2	0.44	7.5	21.4	56.5
C3	UCAAAATTOA$_L$ACUGAGGCA	m	29.3	12.6	100.3	63.1	0.16	2.7	6.9	61.7
		mm	30.0	12.7	99.3	61.4	0.15	2.6	6.8	55.9

Conditions: 0.5 µM probe was measured at 25 °C or 37 °C before (F_0) and after (F) addition of 5 eq. RNA target (match / mismatch: 5'-CUGAAGGACUCACCCUGC**Y**UCAGUUUAUUU UGA-3', UE: Y = C; ED: Y = U). Quantum yield ϕ and brightness Br = $\varepsilon_{\lambda ex} \cdot \phi_{ds}$ / 1000 in mL · mol^{-1} · cm^{-1} were determined at 25 °C. λ_{ex} = 485 nm, λ_{em} = 535 nm. Melting temperature T_m in °C was measured with 1 µM probe and 1 µM target RNA in PBS buffer (100 mM NaCl, 10 mM Na_2HPO_4, pH 7.0). Underscored letters = 2'OMe-RNA; subscript L = LNA; bold letters = base surrogate. Match and mismatch RNA target are indicated with m and mm, respectively.

Table S5 Optical properties and T_M data for ED-QB^2-OMe and ED-QB-OMe FIT probes.

	Sequence 5' – 3'	RNA target	F_0 25 °C	F_0 37 °C	F 25 °C	F 37 °C	ϕ	$ϕ/ϕ_0$	Br	T_m
ED-QB^2-OMe										
1	UCAAA**QB**T$_L$AAACUGA**AQB**C$_L$A	m	5.0	2.1	210.6	125.5	0.24	26.1	20.5	50.7
		mm	4.4	2.0	98.9	26.6	0.13	15.3	8.3	43.2
2	AAAAT**QB**A$_L$ACUGA**AQB**C$_L$A	m	4.9	2.0	142.5	53.4	0.17	20.9	14.1	49.3
		mm	4.4	1.6	36.9	5.2	0.04	6.3	2.6	45.0
3	CAAAAT**QB**A$_L$ACUGA**AQB**C$_L$A	m	4.5	1.6	182.5	104.6	0.22	26.6	18.2	48.8
		mm	4.8	1.9	77.7	21.6	0.10	13.6	6.1	41.9
4	UCAAAAT**QB**A$_L$ACUGA**AQB**C$_L$A	m	4.4	2.0	173.6	97.2	0.22	25.2	17.8	48.6
		mm	4.7	1.8	85.7	30.6	0.10	14.3	6.2	43.6
ED-QB-OMe										
C1	UCAAAAUAAACUGA**AQB**C$_L$A	m	4.8	1.4	136.3	84.6	0.36	20.0	13.9	56.3
		mm	4.5	1.5	46.4	22.8	0.13	7.6	4.7	49.1
C2	UCAAA**QB**T$_L$AAACUGA**A**GCA	m	6.7	3.1	133.5	86.1	0.33	13.5	14.9	60.1
		mm	6.8	3.1	132.4	82.6	0.31	13.5	14.2	53.2
C3	UCAAAAT**QB**A$_L$ACUGA**A**GCA	m	7.4	3.1	63.8	35.0	0.17	8.1	6.2	54.7
		mm	6.8	2.8	56.7	23.4	0.13	6.9	5.3	49.1

Conditions: 0.5 µM probe was measured at 25 °C or 37 °C before (F_0) and after (F) addition of 5 eq. RNA target (match / mismatch: 5'-CUGAAGGACUCACCCUGC**Y**UCAGUUUAUUU UGA-3', UE: Y = C; ED: Y = U). Quantum yield ϕ and brightness $Br = \varepsilon_{\lambda ex} \cdot ϕ_{ds} / 1000$ in $mL \cdot mol^{-1} \cdot cm^{-1}$ were determined at 25 °C. λ_{ex} = 560 nm, λ_{em} = 605 nm. Melting temperature T_m in °C was measured with 1 µM probe and 1 µM target RNA in PBS buffer (100 mM NaCl, 10 mM Na_2HPO_4, pH 7.0). Underscored letters = 2'OMe-RNA; subscript L = LNA; bold letters = base surrogate. Match and mismatch RNA target are indicated with m and mm, respectively.

Table S6 Optical properties and T_M data for ED-TO^2-OMe and ED-TO-OMe FIT probes.

	Sequence 5' – 3'	RNA target	F_0		F		ϕ	ϕ/ϕ$_0$	Br	T_m
			25 °C	37 °C	25 °C	37 °C				
ED-TO^2-OMe										
1	UCAAA**TO**T$_L$AAACUGA**ATO**C$_L$A	m	126.5	41.5	449.2	270.2	0.44	3.4	46.6	52.3
		mm	129.1	41.8	234.7	89.8	0.25	1.8	27.4	45.1
2	AAAAT**TO**A$_L$ACUGA**ATO**C$_L$A	m	31.4	15.4	185.5	126.2	0.19	4.5	18.7	50.5
		mm	33.0	16.1	75.3	22.9	0.09	2.0	9.3	45.6
3	CAAAAT**TO**A$_L$ACUGA**ATO**C$_L$A	m	30.7	15.1	197.6	105.8	0.19	4.7	19.5	50.3
		mm	24.9	11.8	86.3	29.1	0.10	3.0	7.5	42.4
4	UCAAAAT**TO**A$_L$ACUGA**ATO**C$_L$A	m	29.1	14.0	195.1	106.6	0.19	4.9	19.3	50.0
		mm	21.8	10.4	73.8	24.2	0.08	2.8	6.3	42.3
ED-TO-OMe										
C1	UCAAAAUAAACUGA**ATO**C$_L$A	m	72.2	12.8	245.2	169.1	0.38	3.1	18.2	56.1
		mm	70.9	13.0	88.2	52.2	0.17	7.8	7.8	49.5
C2	UCAAA**TO**T$_L$AAACUGA**A**GCA	m	34.8	19.0	285.0	195.4	0.54	27.5	27.5	60.4
		mm	34.8	19.3	279.9	191.2	0.46	23.0	23.0	53.4
C3	UCAAAAT**TO**A$_L$ACUGA**A**GCA	m	61.5	15.1	197.8	116.7	0.28	12.8	12.8	52.4
		mm	60.3	15.1	309.1	223.8	0.43	20.1	20.1	61.8

Conditions: 0.5 µM probe was measured at 25 °C or 37 °C before (F_0) and after (F) addition of 5 eq. RNA target (match / mismatch: 5'-CUGAAGGACUCACCCUGC**Y**UCAGUUUAUUU UGA-3', UE: Y = C; ED: Y = U). Quantum yield ϕ and brightness Br = $\varepsilon_{\lambda ex} \cdot \phi_{ds}$ / 1000 in mL · mol^{-1} · cm^{-1} were determined at 25 °C. λ_{ex} = 560 nm, λ_{em} = 605 nm. Melting temperature T_m in °C was measured with 1 µM probe and 1 µM target RNA in PBS buffer (100 mM NaCl, 10 mM Na_2HPO_4, pH 7.0). Underscored letters = 2'OMe-RNA; subscript L = LNA; bold letters = base surrogate. Match and mismatch RNA target are indicated with m and mm, respectively.

4. Fluorescence and Absorption Spectra

Fluorescent excitation (dashed lines) and emission (solid line) spectra (left row) and extinction coefficient as function of absorption (right column).

UE-QB^2 and UE-QB FIT probes

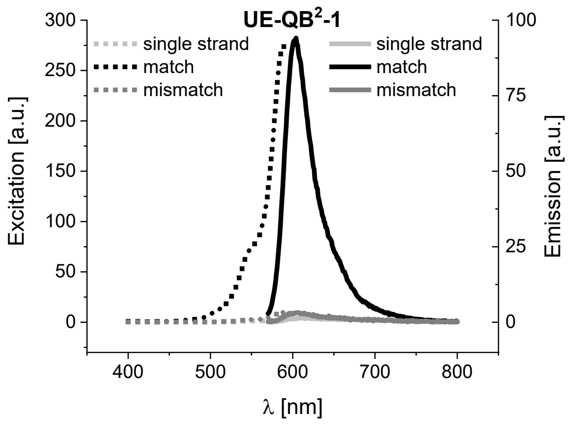

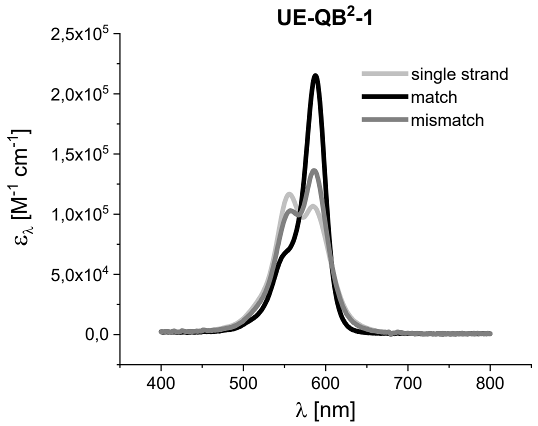

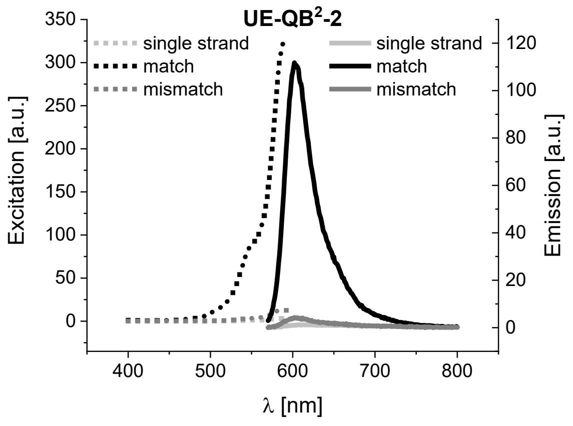

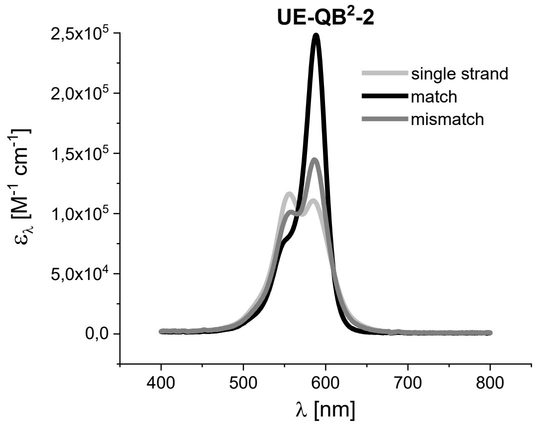

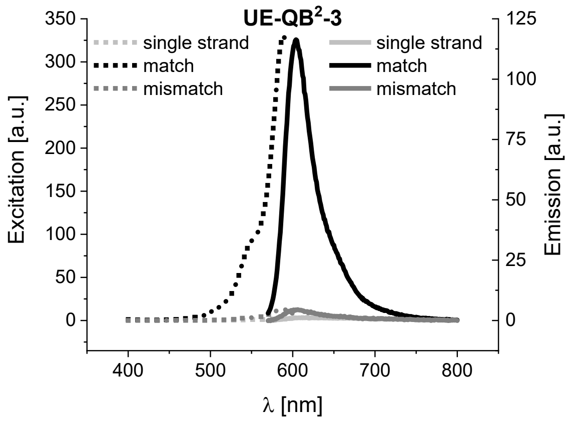

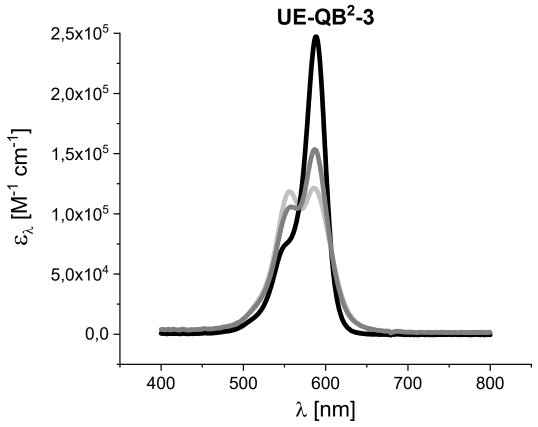

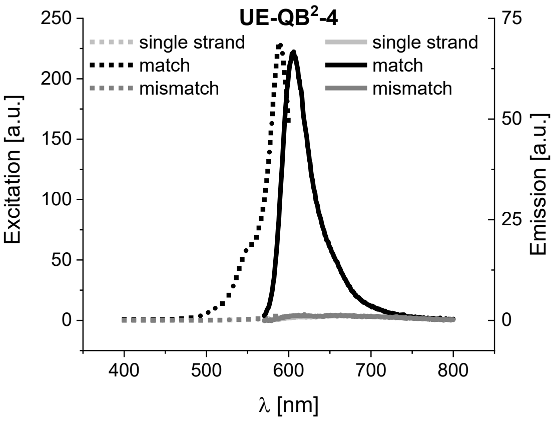
UE-QB²-4
single strand
match
mismatch
single strand
match
mismatch
Excitation [a.u.]
Emission [a.u.]
λ [nm]

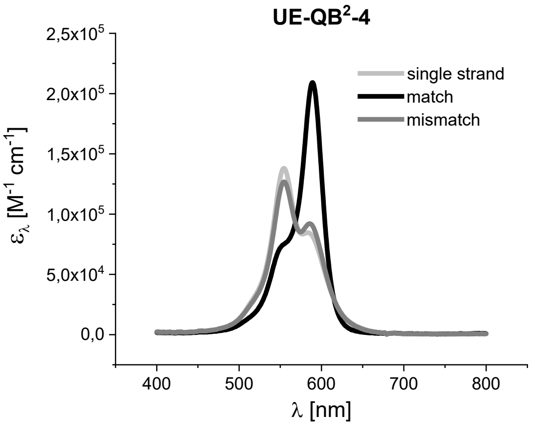
UE-QB²-4
single strand
match
mismatch
ελ [M⁻¹ cm⁻¹]
λ [nm]

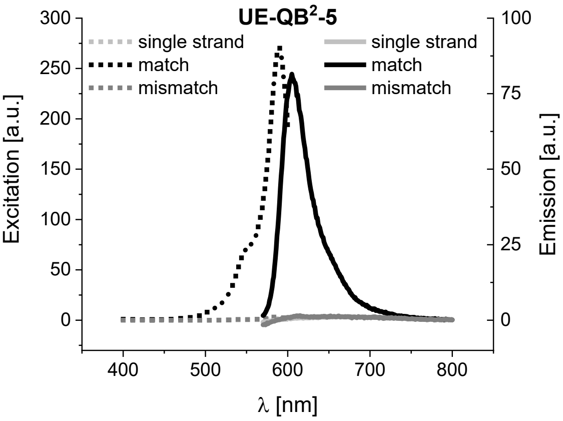
UE-QB²-5
single strand
match
mismatch
single strand
match
mismatch
Excitation [a.u.]
Emission [a.u.]
λ [nm]

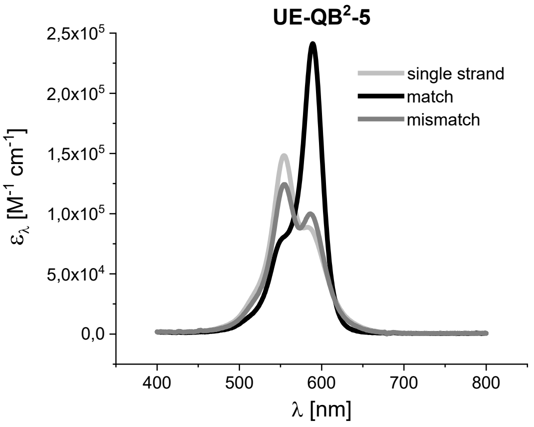
UE-QB²-5
single strand
match
mismatch
ελ [M⁻¹ cm⁻¹]
λ [nm]

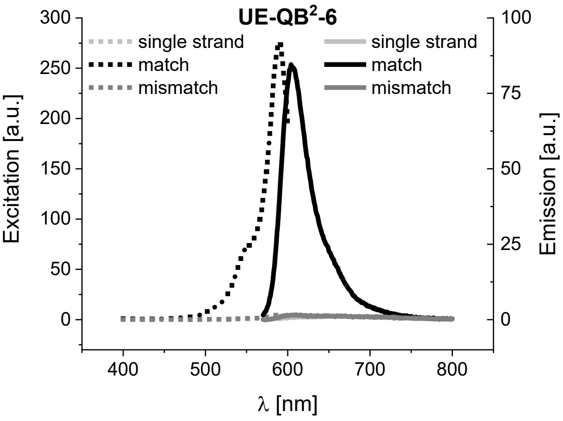
UE-QB²-6
single strand
match
mismatch
single strand
match
mismatch
Excitation [a.u.]
Emission [a.u.]
λ [nm]

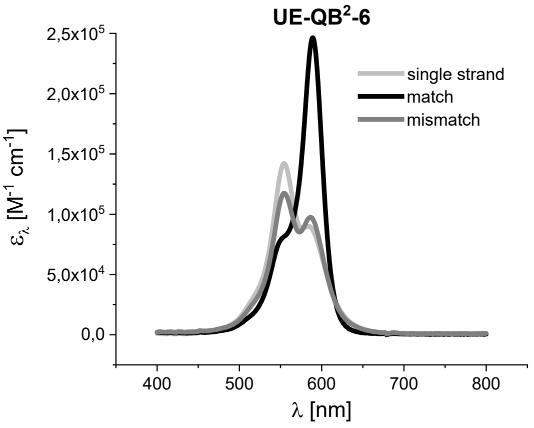
UE-QB²-6
single strand
match
mismatch
ελ [M⁻¹ cm⁻¹]
λ [nm]

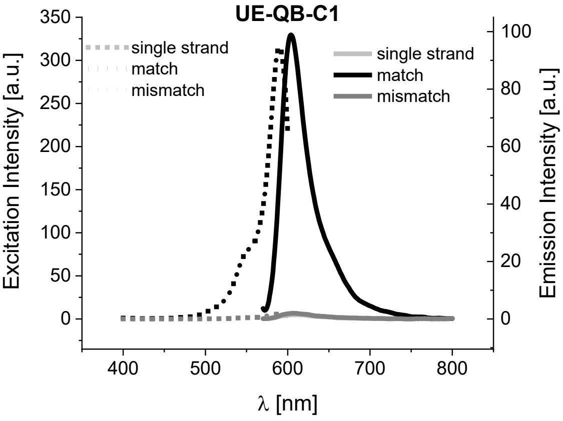
UE-QB-C1
single strand
match
mismatch
single strand
match
mismatch
Excitation Intensity [a.u.]
Emission Intensity [a.u.]
λ [nm]

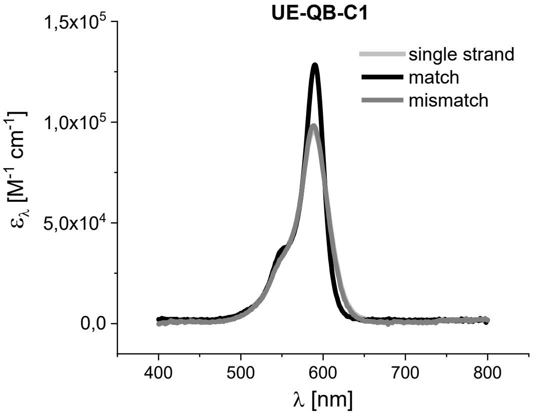
UE-QB-C1
single strand
match
mismatch
ελ [M⁻¹ cm⁻¹]
λ [nm]

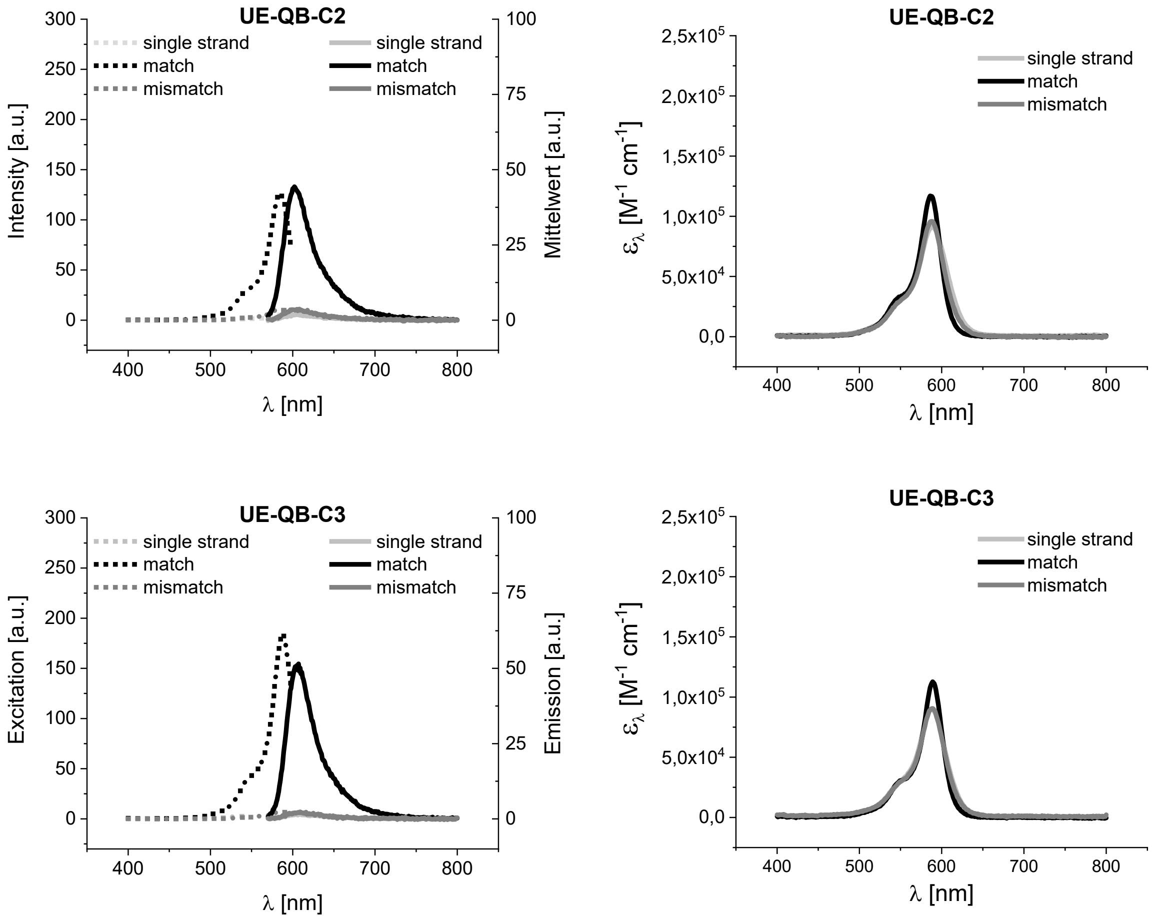

Figure S1 Fluorescence and absorption spectra UE-QB[2] and UE-QB FIT probes. Conditions: 0.5 µM probe was measured at 37 °C before and after addition of 5 eq. RNA target (match / mismatch: 5’-CUGAAGGACUCACCCUGC**Y**UCAGUUUAUUU UGA-3', UE: Y = C; ED: Y = U). λ_{ex} = 560 nm, λ_{em} = 605 nm.

UE-QB^2-OMe and UE-QB-OMe FIT probes

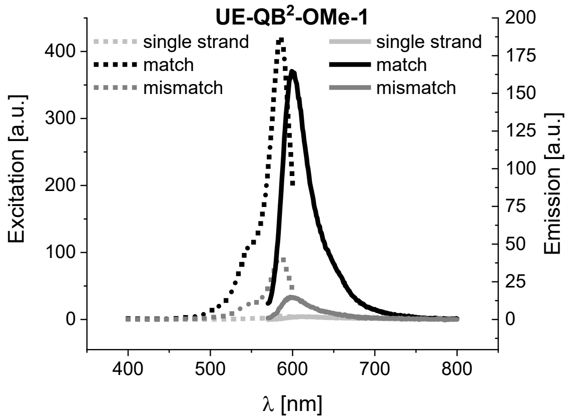

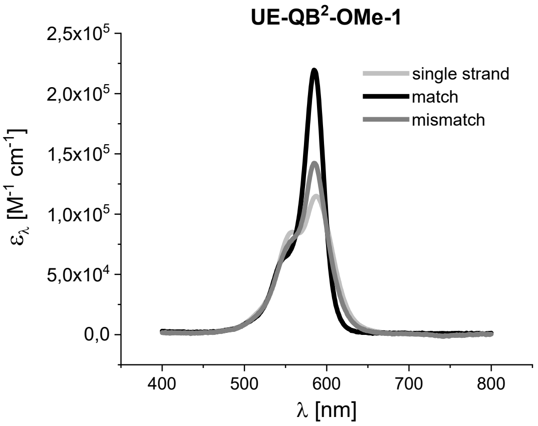

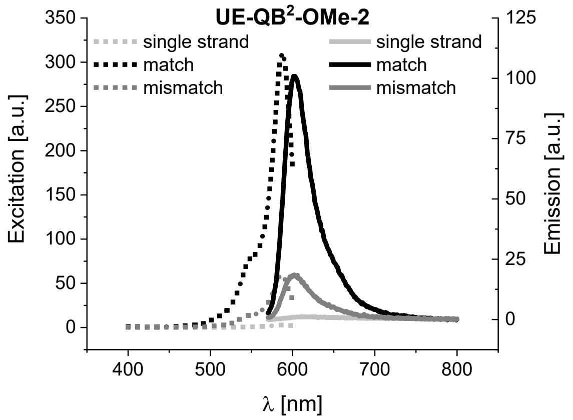

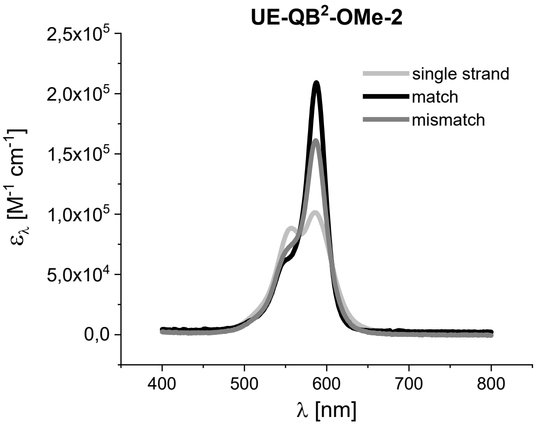

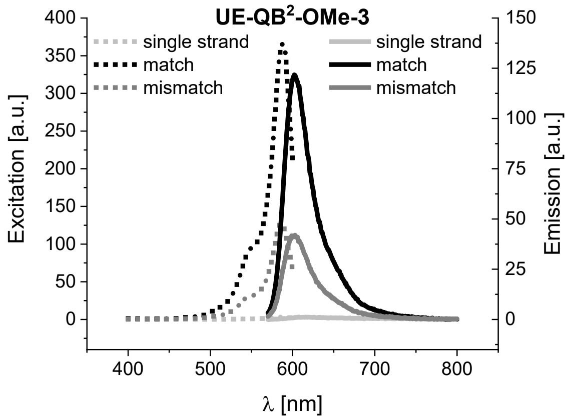

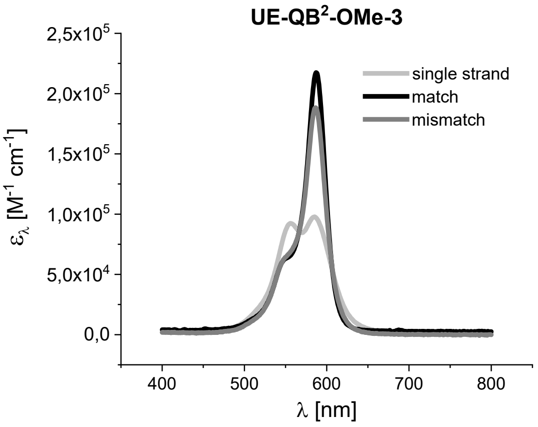

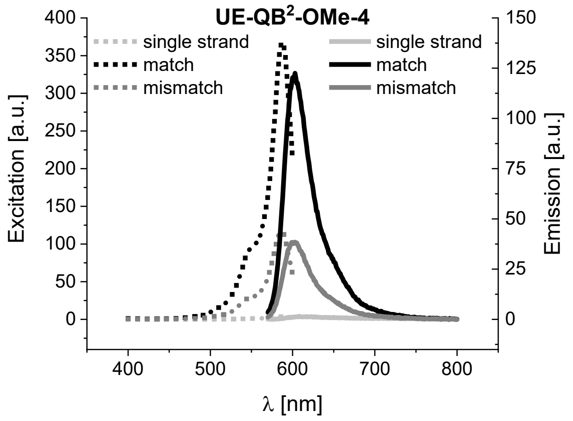

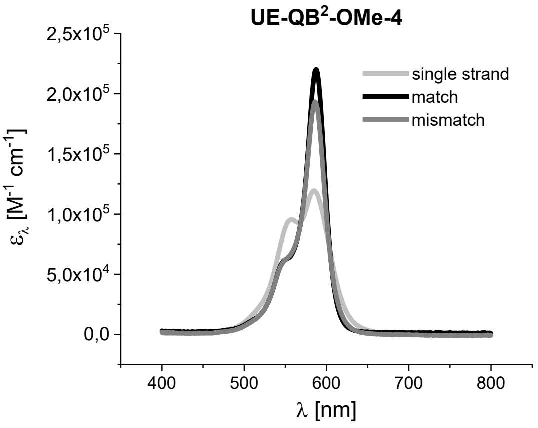

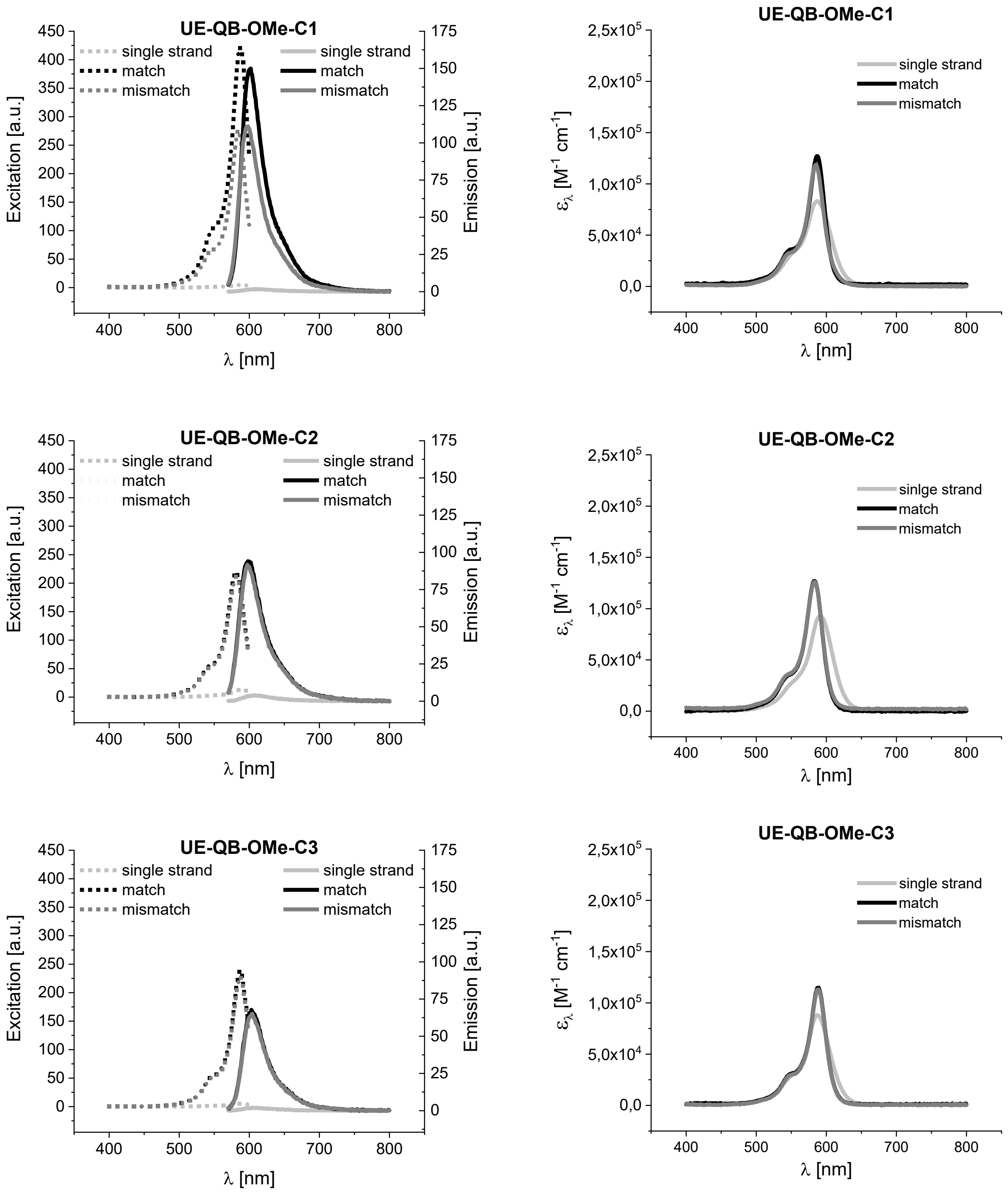

Figure S2 Fluorescence and absorption spectra UE-QB^2-OMe and UE-QB-OMe FIT probes. Conditions: 0.5 µM probe was measured at 37 °C before and after addition of 5 eq. RNA target (match / mismatch: 5'-CUGAAGGACUCACCCUGC**Y**UCAGUUUAUUU UGA-3', UE: Y = C; ED: Y = U). λ_{ex} = 560 nm, λ_{em} = 605 nm.

UE-TO^2-OMe and UE-TO-OMe FIT probes

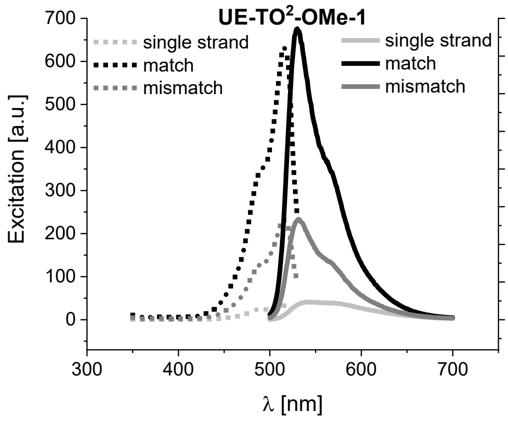

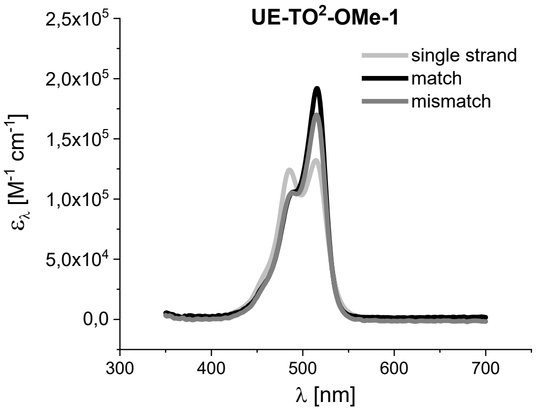

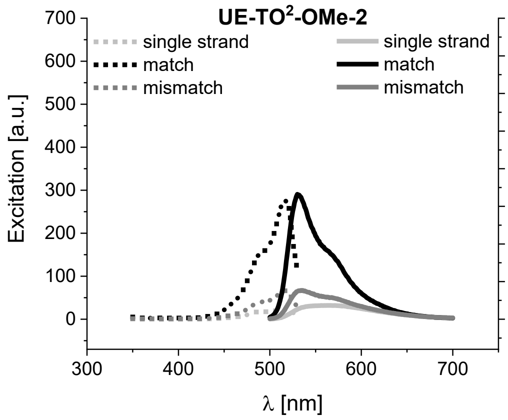

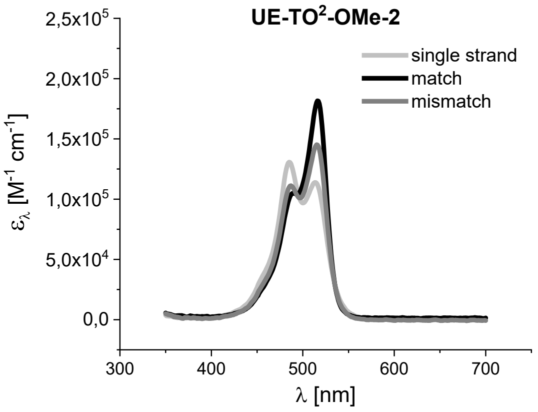

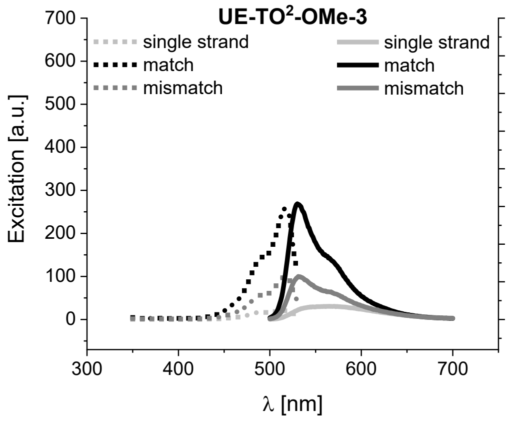

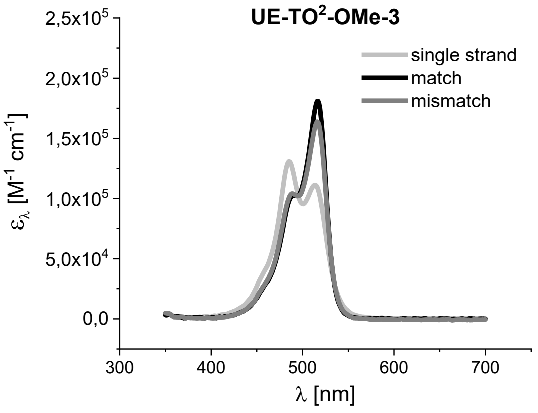

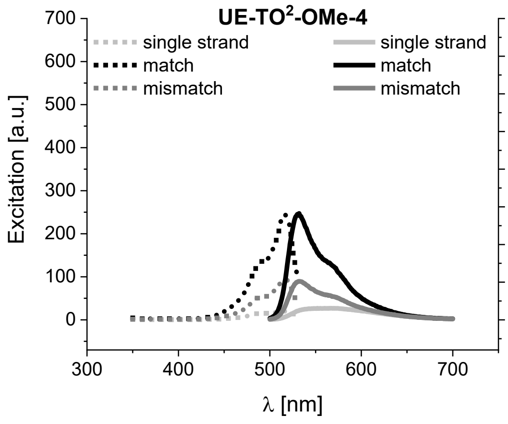

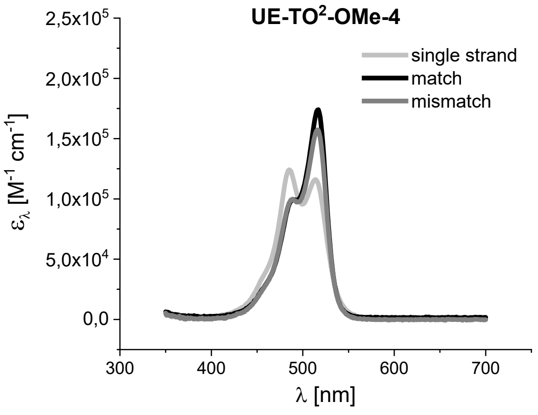

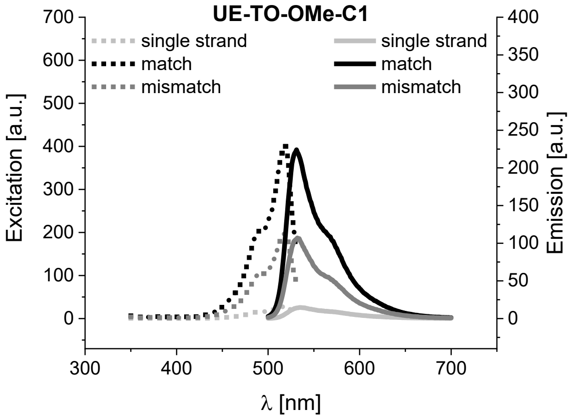
UE-TO-OMe-C1
single strand
match
mismatch
single strand
match
mismatch
Excitation [a.u.]
Emission [a.u.]
λ [nm]

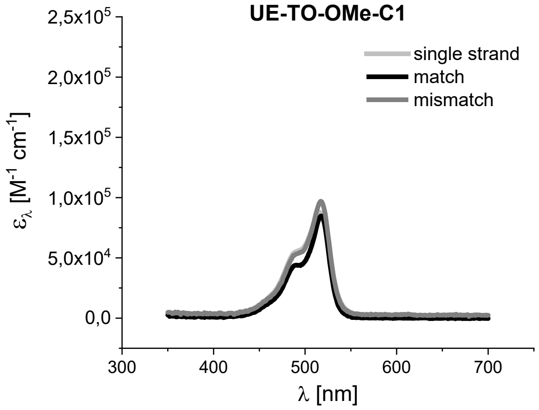
UE-TO-OMe-C1
single strand
match
mismatch
ε_λ [M^{-1} cm^{-1}]
λ [nm]

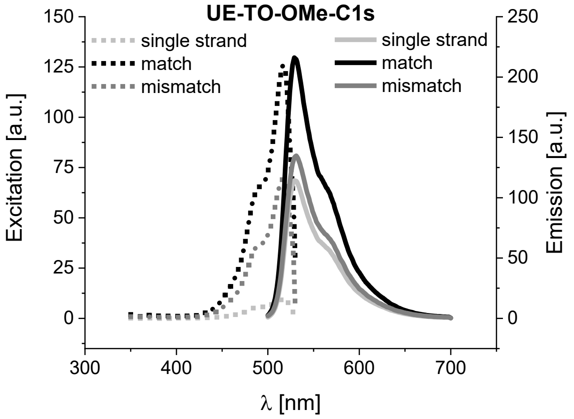
UE-TO-OMe-C1s
single strand
match
mismatch
single strand
match
mismatch
Excitation [a.u.]
Emission [a.u.]
λ [nm]

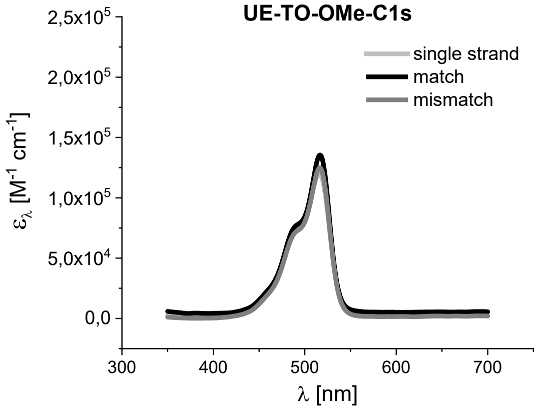
UE-TO-OMe-C1s
single strand
match
mismatch
ε_λ [M^{-1} cm^{-1}]
λ [nm]

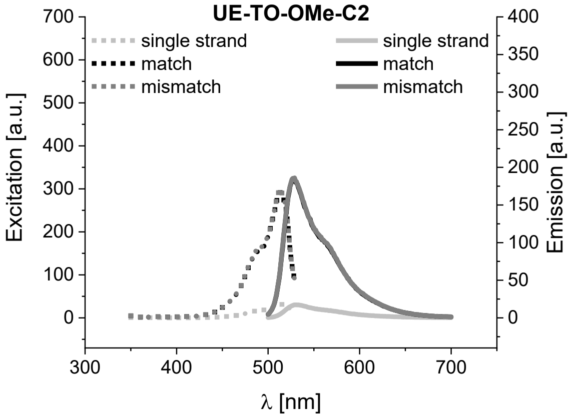
UE-TO-OMe-C2
single strand
match
mismatch
single strand
match
mismatch
Excitation [a.u.]
Emission [a.u.]
λ [nm]

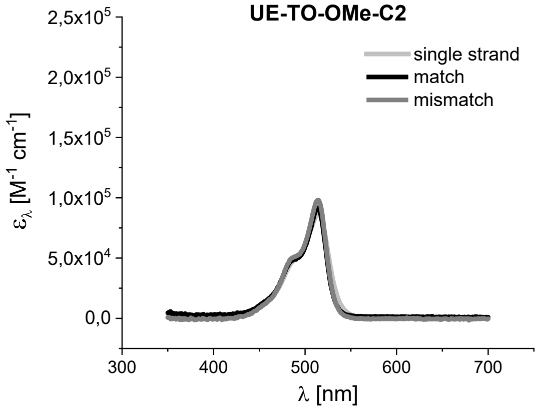
UE-TO-OMe-C2
single strand
match
mismatch
ε_λ [M^{-1} cm^{-1}]
λ [nm]

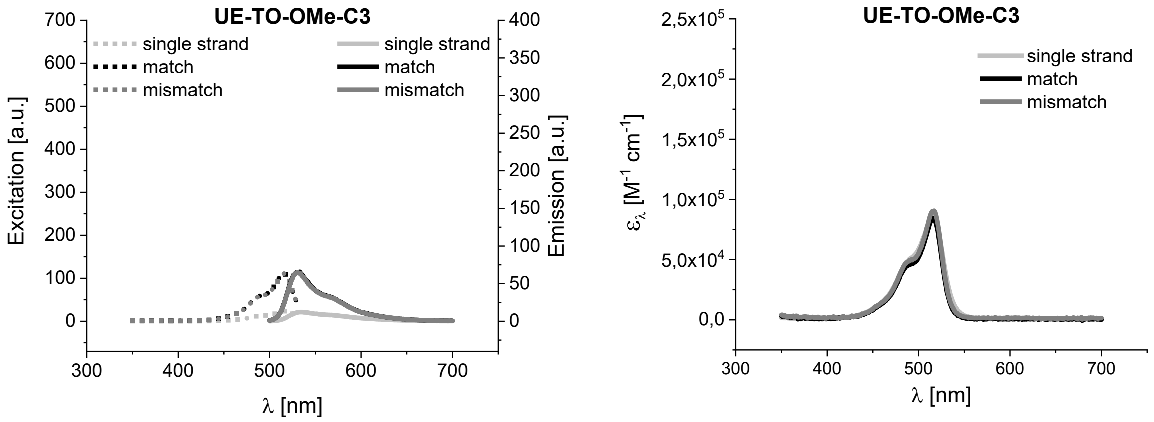

Figure S3 Fluorescence and absorption spectra UE-TO^2-OMe and UE-TO-OMe FIT probes. Conditions: 0.5 µM probe was measured at 37 °C before and after addition of 5 eq. RNA target (match / mismatch: 5’-CUGAAGGACUCACCCUGC**Y**UCAGUUUAUUU UGA-3', UE: Y = C; ED: Y = U). λ_{ex} = 485 nm, λ_{em} = 535 nm.

ED-QB^2-OMe and ED-QB-OMe FIT probes

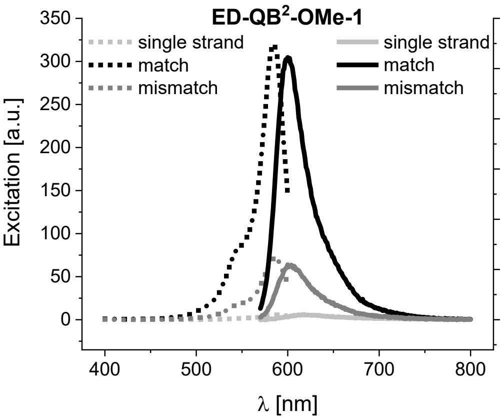

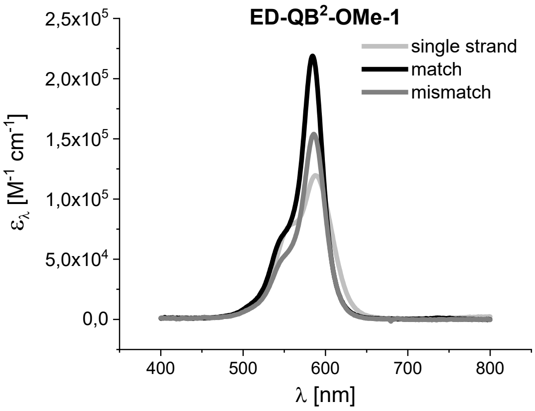

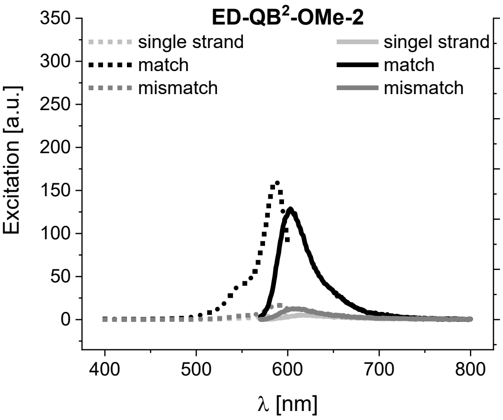

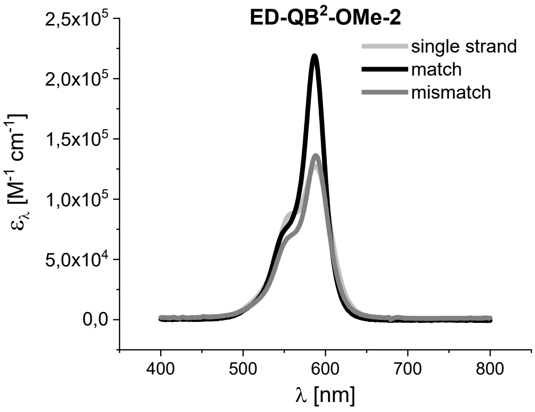

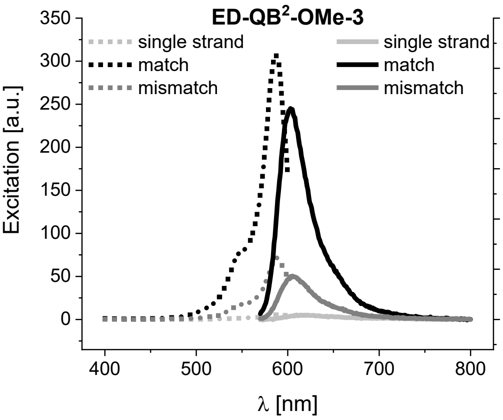

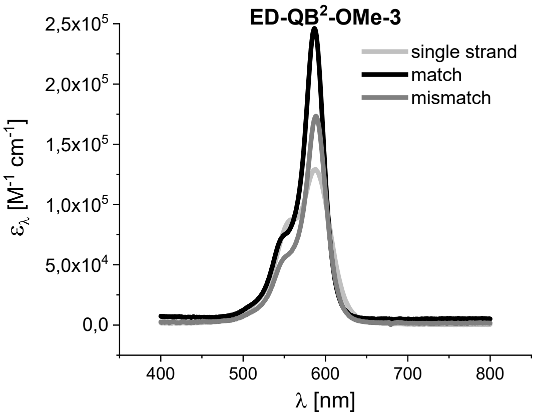

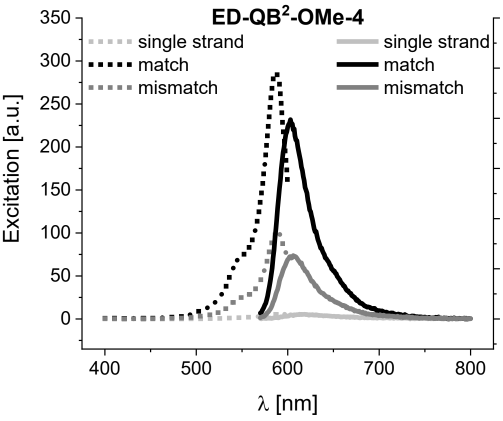

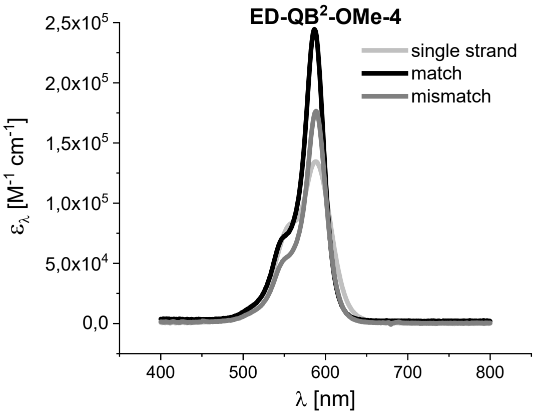

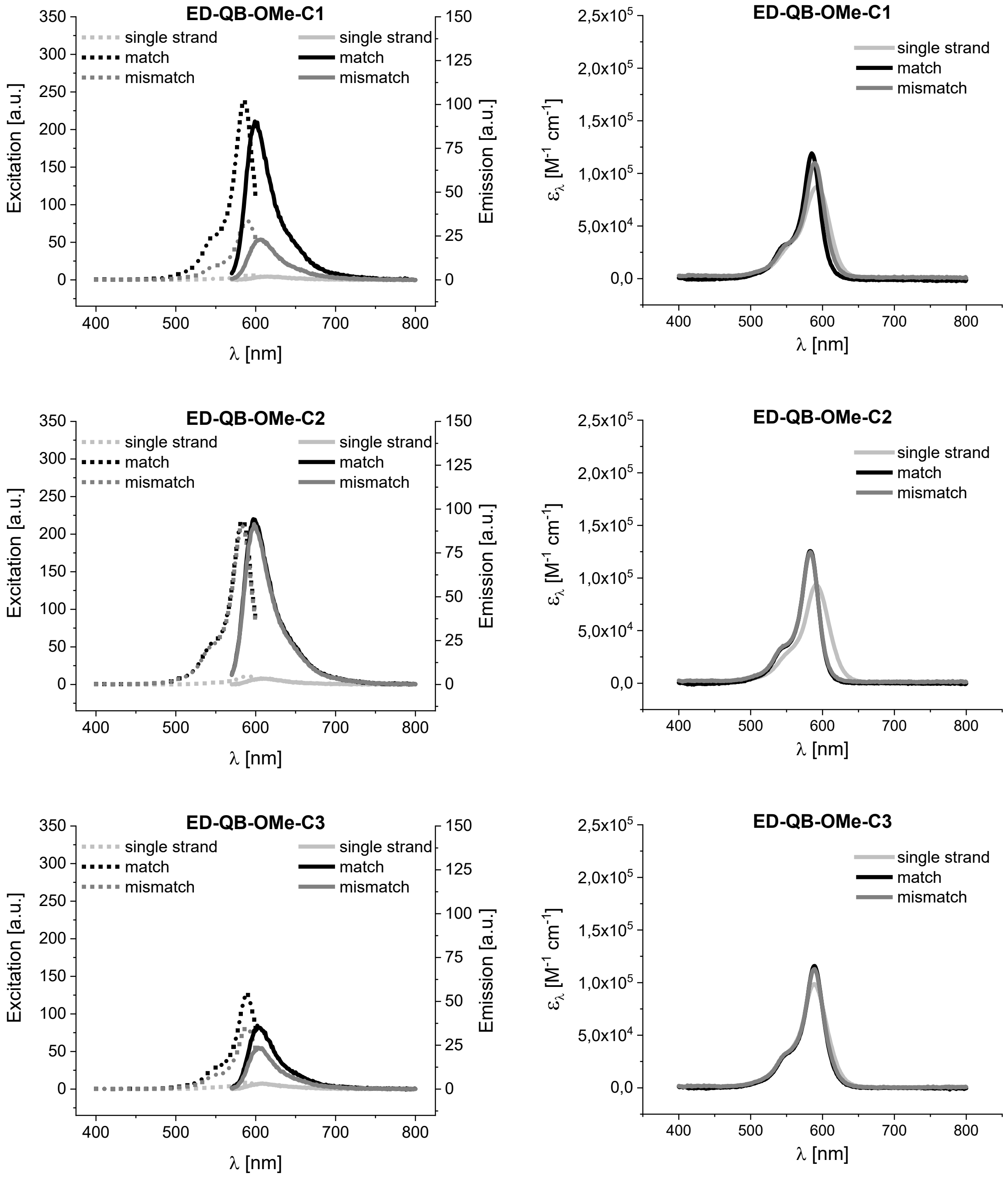

Figure S4 Fluorescence and absorption spectra ED-QB^2-OMe and ED-QB-OMe FIT probes. Conditions: 0.5 µM probe was measured at 37 °C before and after addition of 5 eq. RNA target (match / mismatch: 5'-CUGAAGGACUCACCCUGC**Y**UCAGUUUAUUU UGA-3', UE: Y = C; ED: Y = U). λ_{ex} = 560 nm, λ_{em} = 605 nm.

ED-TO^2-OMe and ED-TO-OMe FIT probes

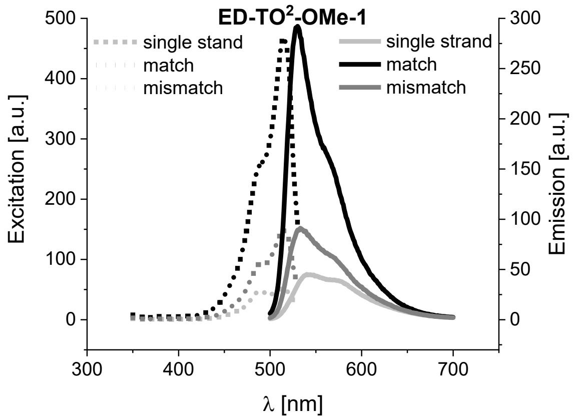

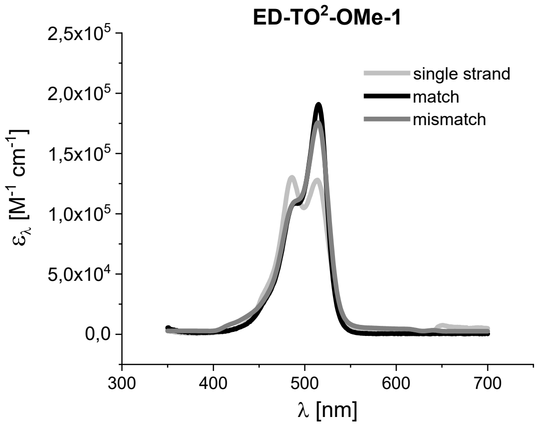

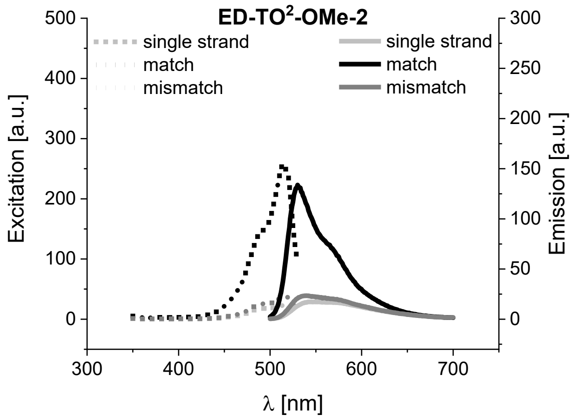

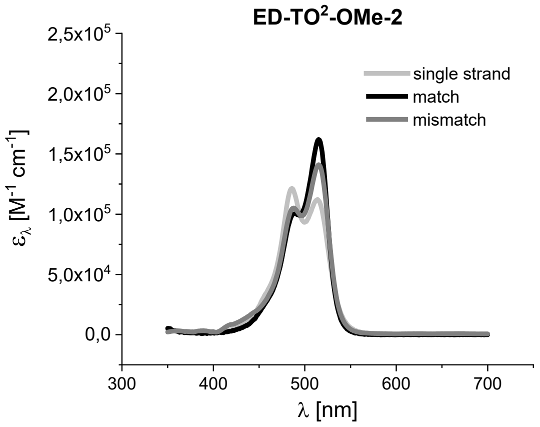

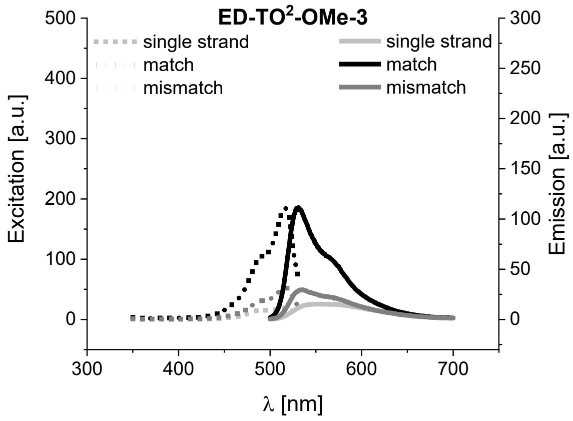

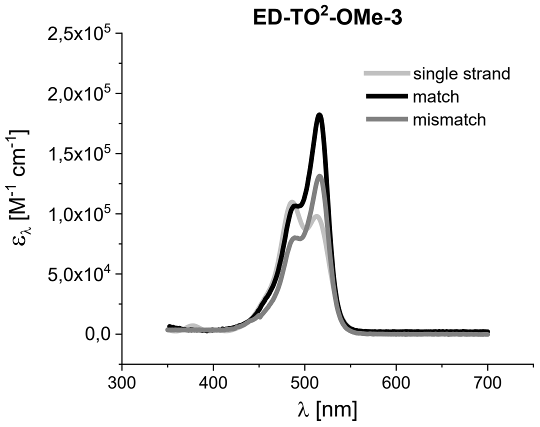

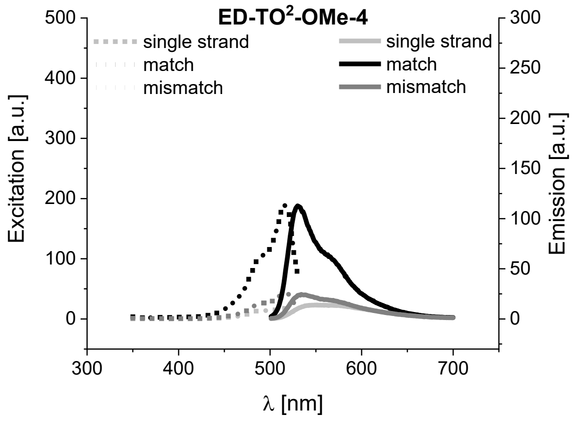

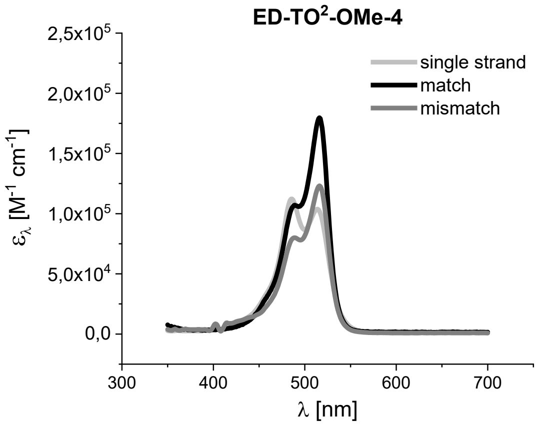

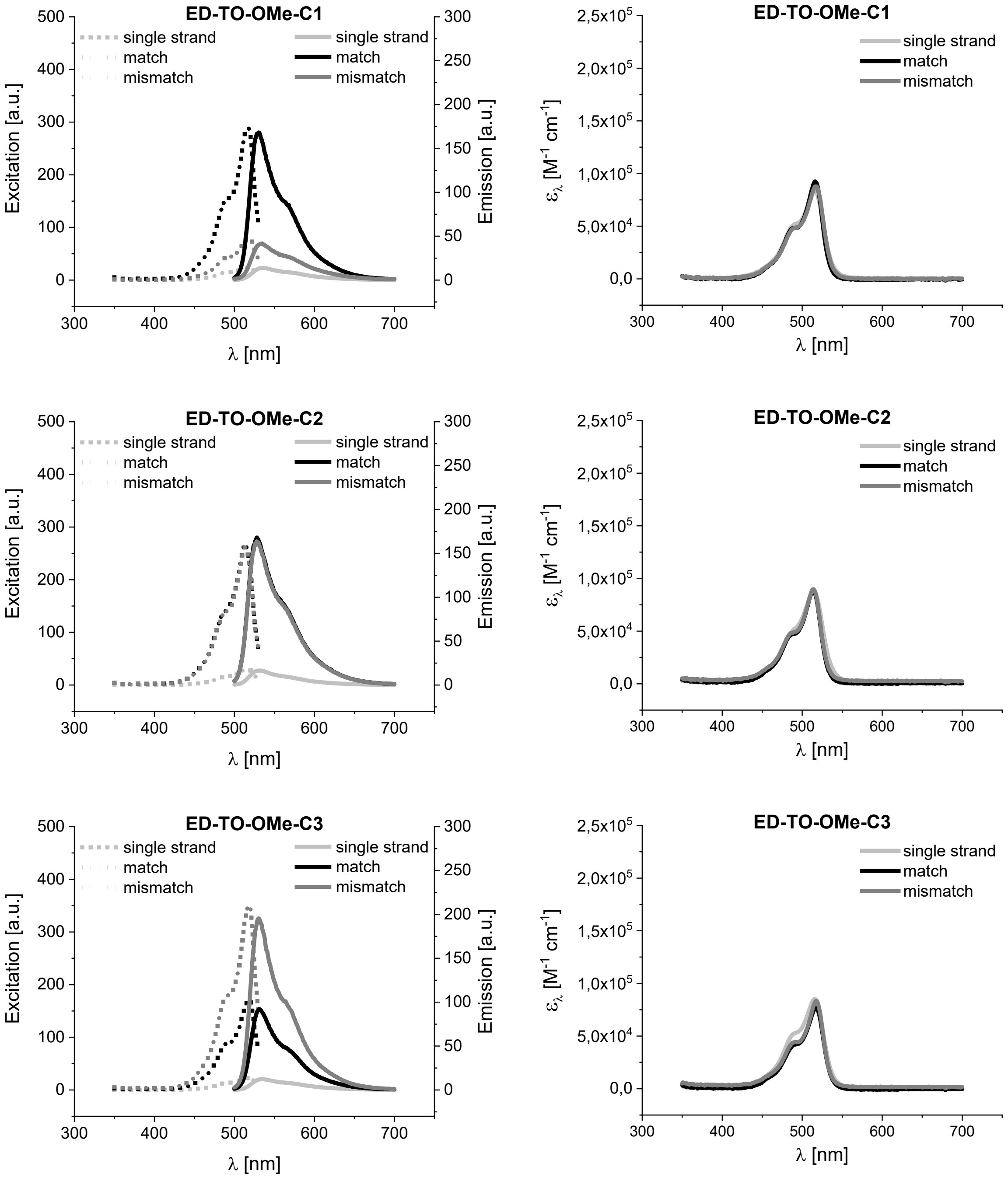

Figure S5 Fluorescence and absorption spectra ED-TO^2-OMe and ED-TO-OMe FIT probes. Conditions: 0.5 µM probe was measured at 37 °C before and after addition of 5 eq. RNA target (match / mismatch: 5'-CUGAAGGACUCACCCUGC**Y**UCAGUUUAUUU UGA-3', UE: Y = C; ED: Y = U). λ_{ex} = 485 nm, λ_{em} = 535 nm.

Normalised Emission of Selected FIT Probes

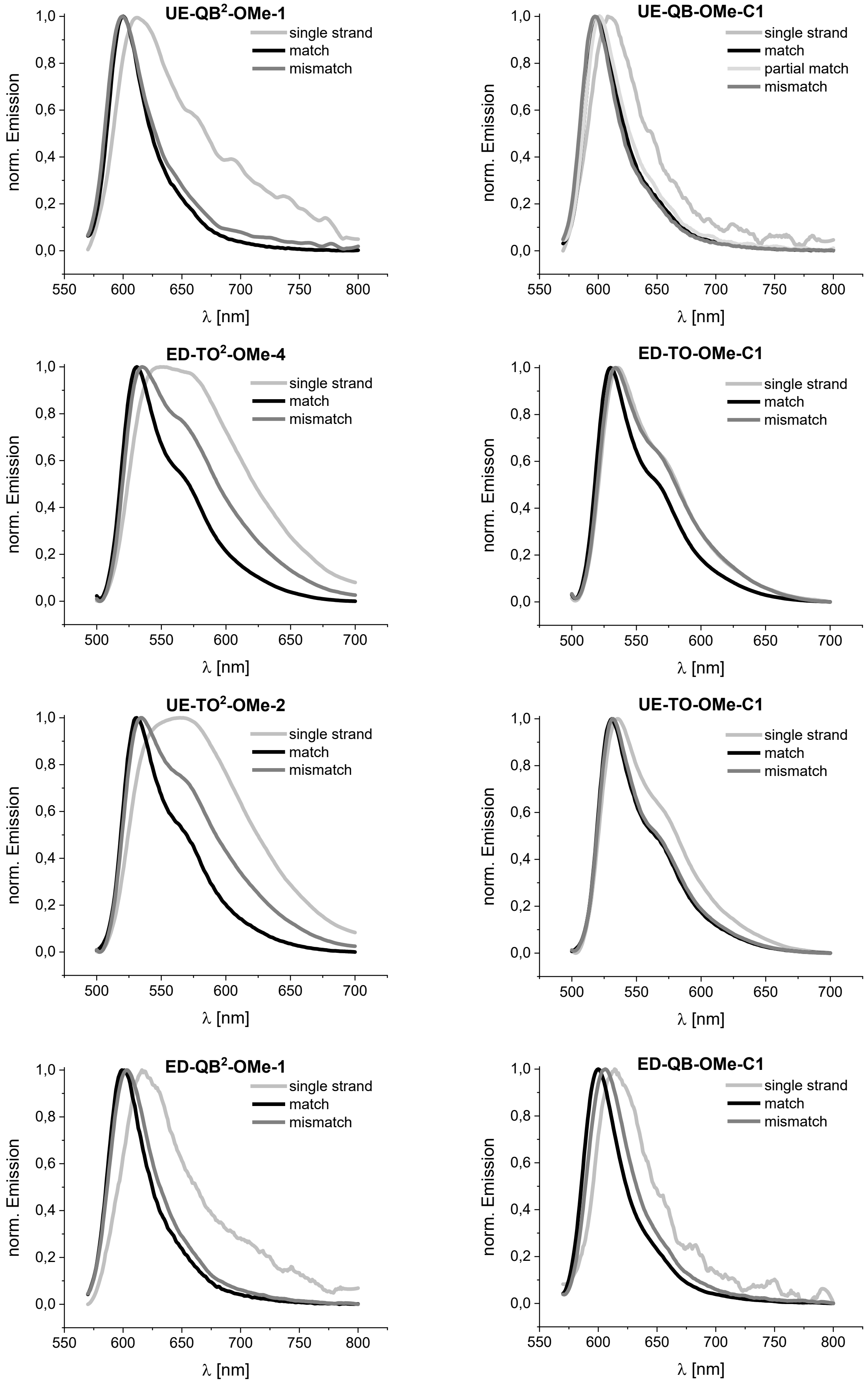

Figure S6 Normalized emission of selected FIT^2 probes and corresponding mono dye FIT probes.

5. Melting Curves

UE-QB^2 and UE-QB FIT probes

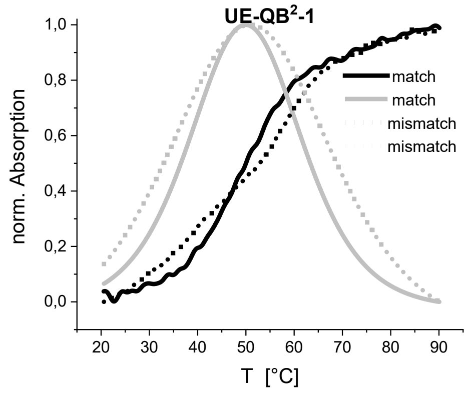

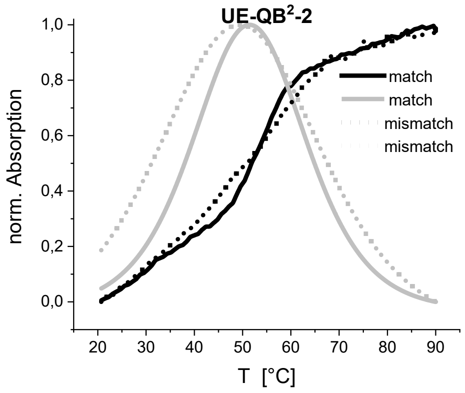

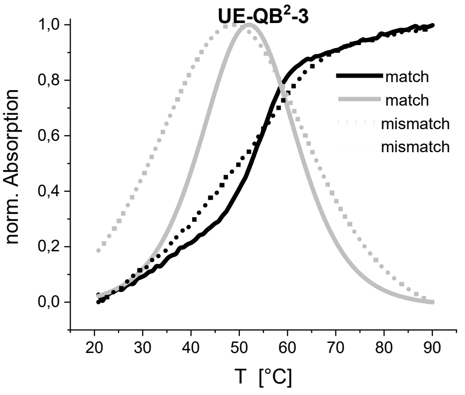

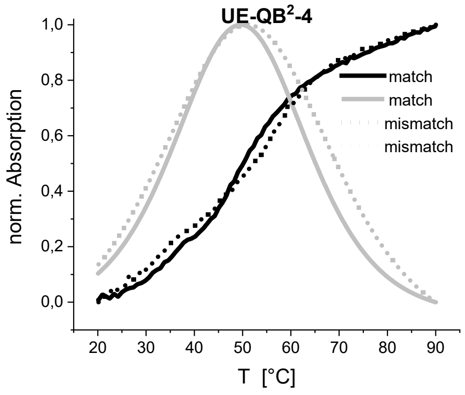

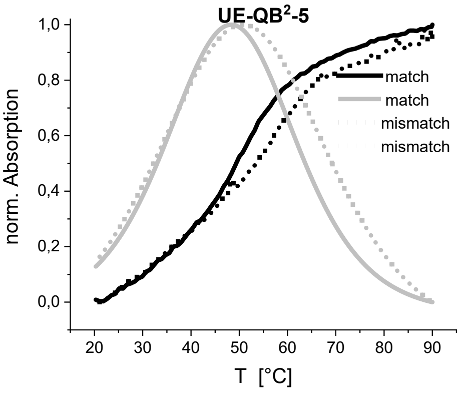

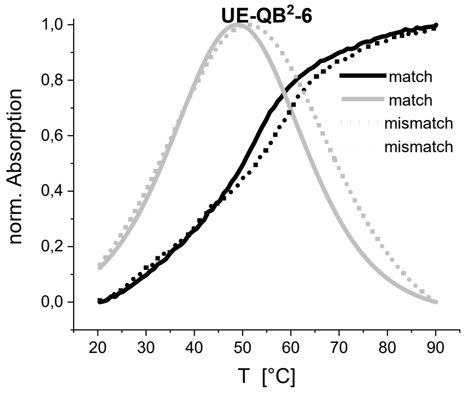

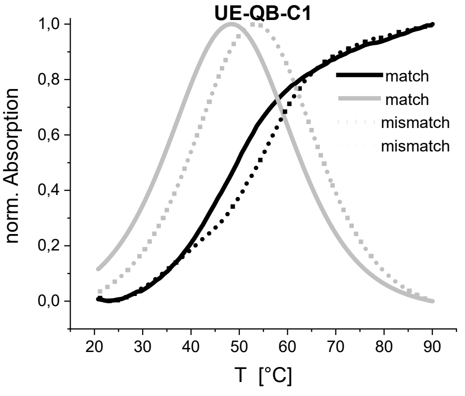

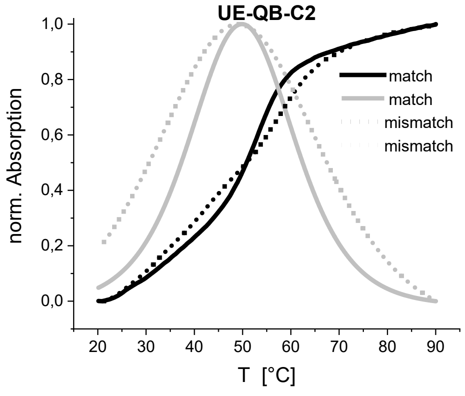

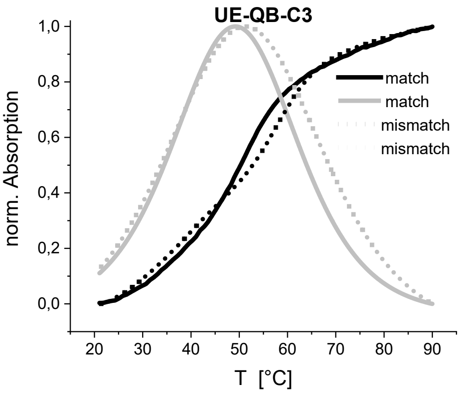

Figure S7 Melting curves UE-QB^2 and UE-QB FIT probes. Grey curves show the 1st derivative of the sigmoidal fit.

UE-QB^2-OMe and Controls

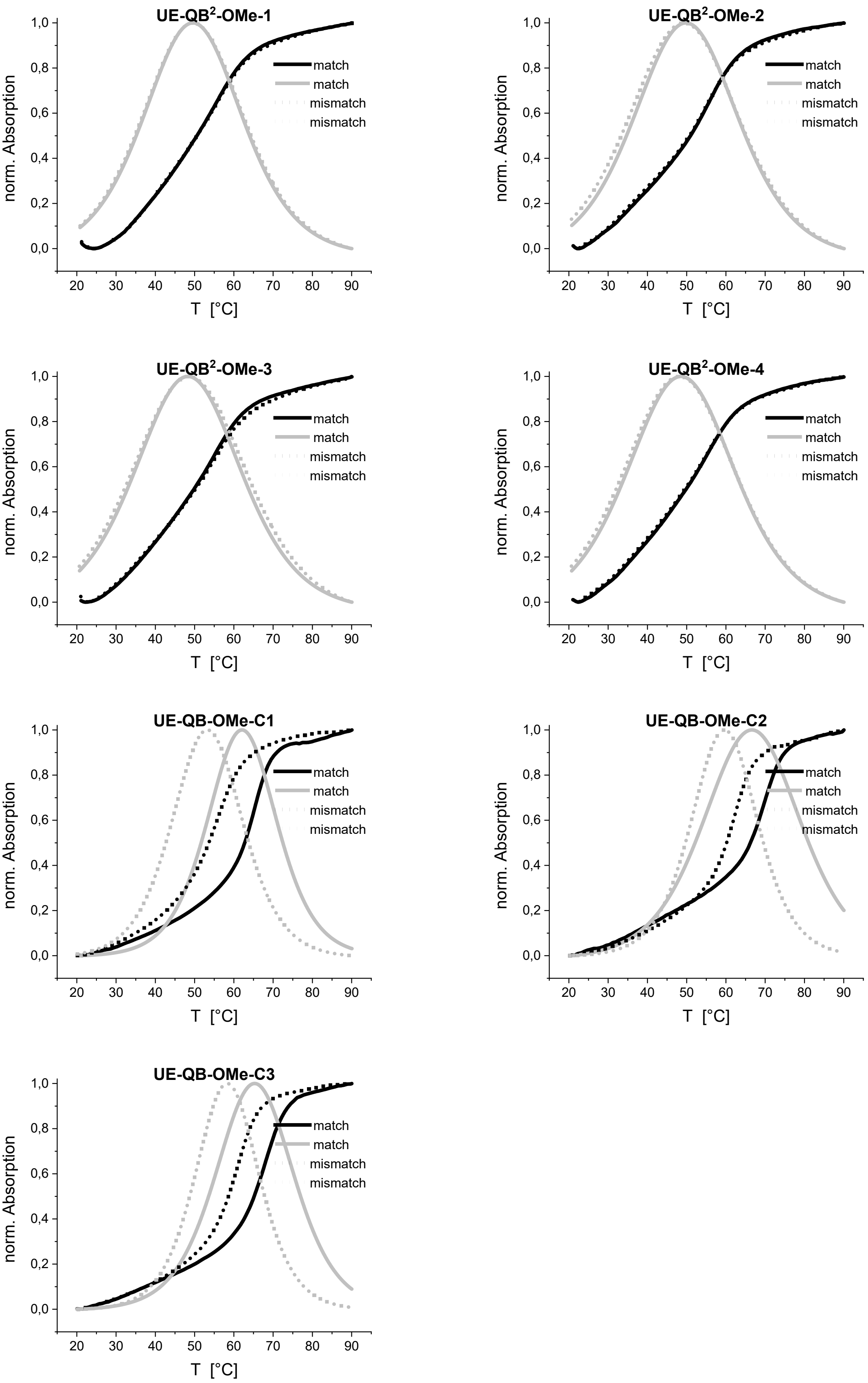

Figure S8 Melting curves UE-QB^2-OMe and UE-QB-OMe FIT probes. Grey curves show the 1st derivative of the sigmoidal fit.

UE-TO^2-OMe and UE-TO-OMe FIT probes

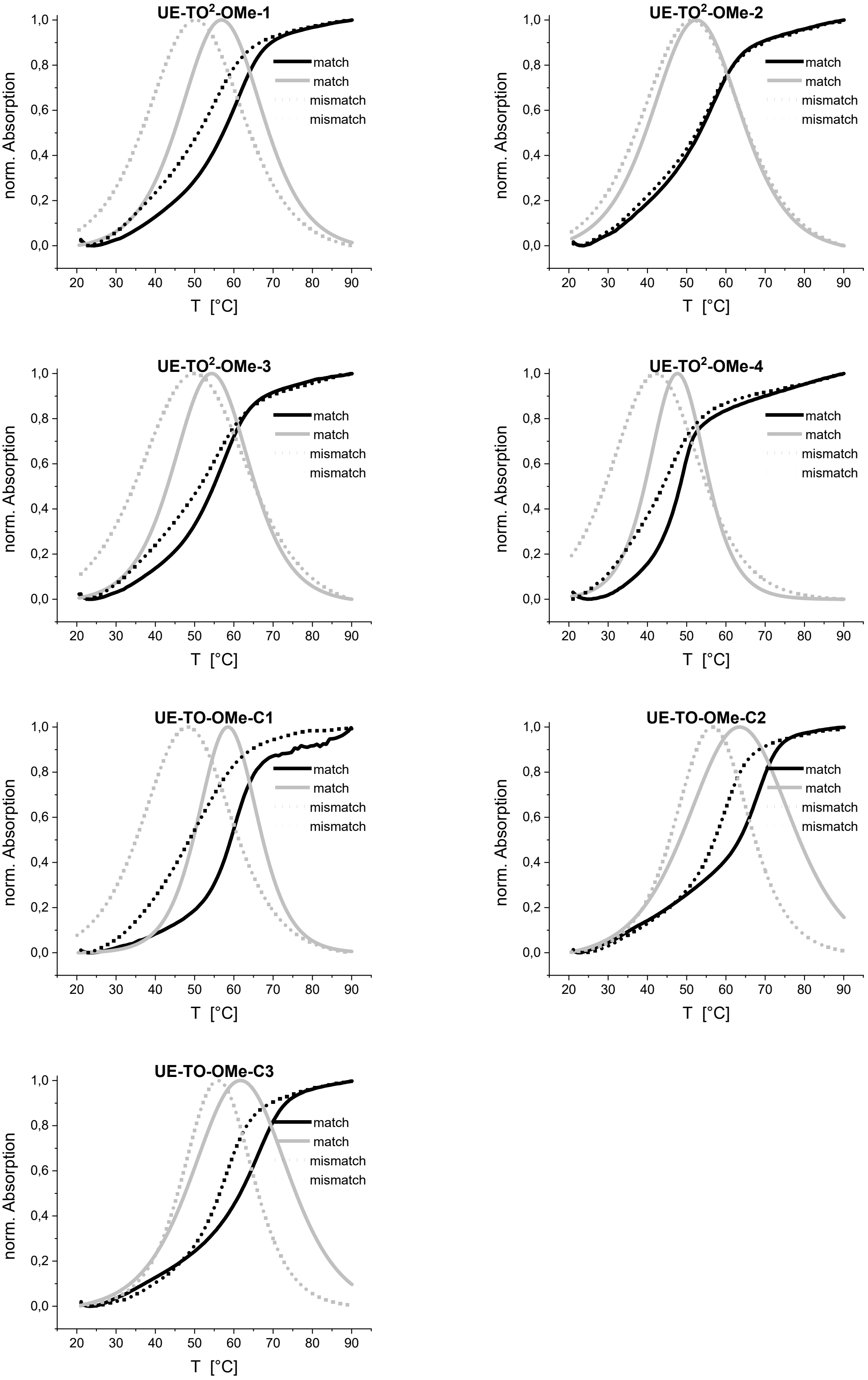

Figure S9 Melting curves UE-TO^2-OMe and controls. Grey curves show the 1st derivative of the sigmoidal fit.

ED-QB^2-OMe and ED-QB-OMe FIT probes

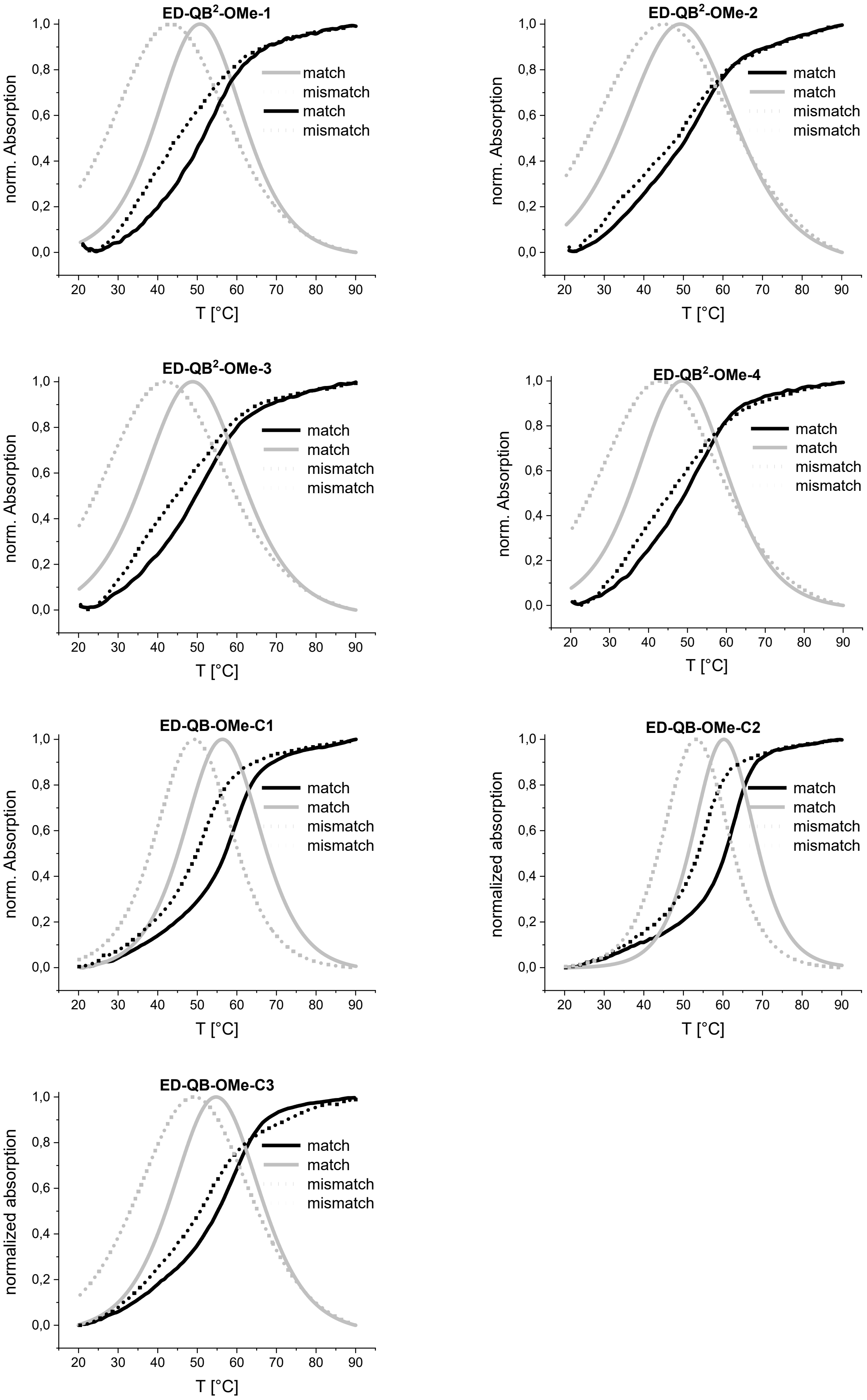

Figure S10 Melting curves of ED-QB^2-OMe and ED-QB-OMe FIT probes. Grey curves show the 1st derivative of the sigmoidal fit.

ED-TO^2-OMe and ED-TO-OMe FIT probes

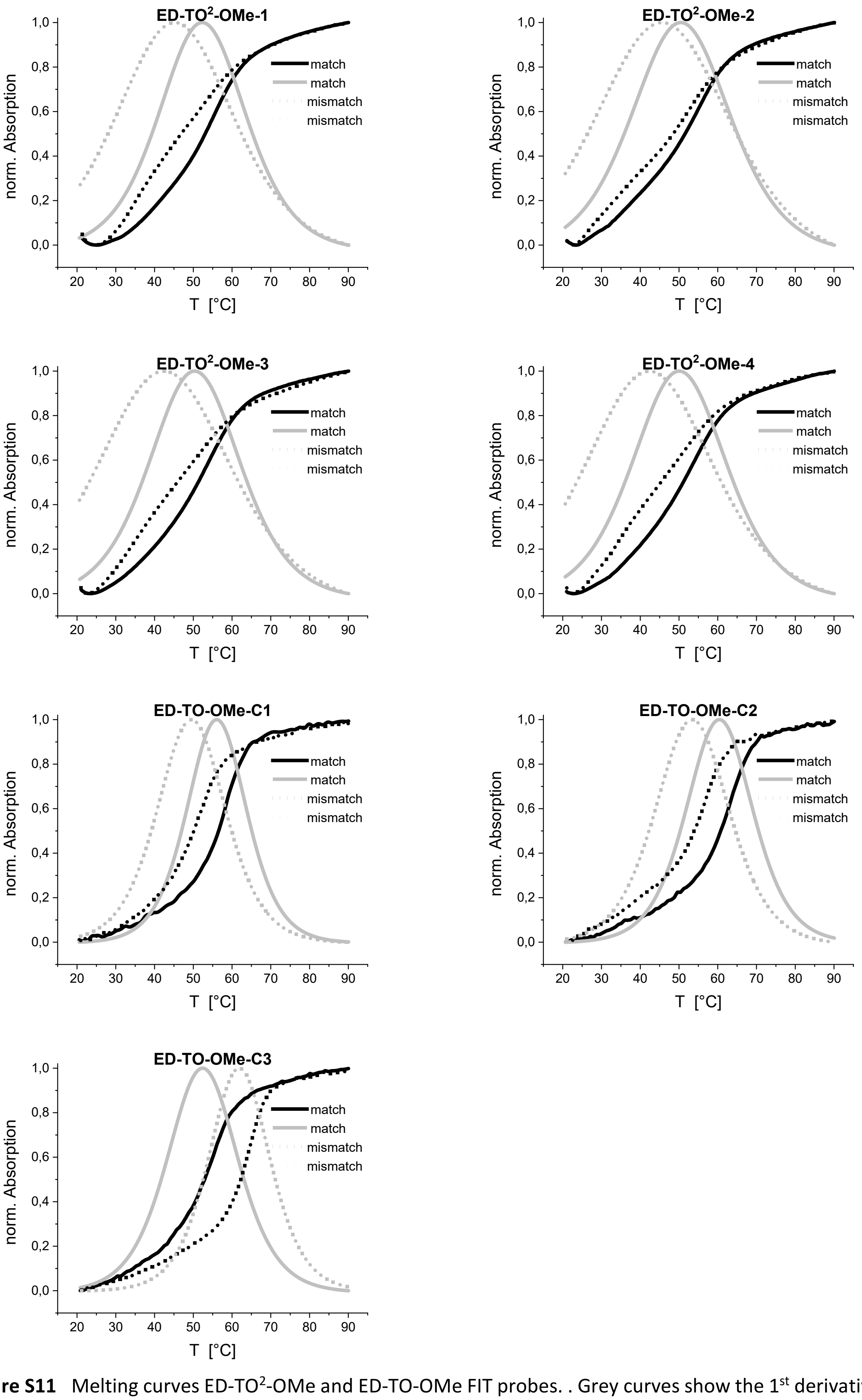

Figure S11 Melting curves ED-TO^2-OMe and ED-TO-OMe FIT probes. . Grey curves show the 1st derivative of the sigmoidal fit.

6. Competitive Hybridization in Cell Lysate

Preparation of Cell Lysate

HEK 293 cells were grown in DMEM supplemented with 10% fetal calf serum, 4 mM Glutamine and 1% 1 % Penicillin/Streptavidin mixture at 37 °C in a humidified 5 % CO_2 atmosphere and sub-cultivated twice a week. 2x10^8 HEK 293 cells were pelleted by centrifugation (500 x g, 5 min), washed with PBS buffer and lysed by 15 min incubation in 4 ml lysis buffer (10 mM Na_2HPO_4, 100 mM NaCl, 0.5 % Triton X-100, pH 7.0, 1 U/µl RNAsin Plus RNAse inhibitor [*Promega,* Madison, WI, USA], cOmplete Mini protease inhibitor cocktail tablets [*Sigma-Aldrich,* St. Louis, MO, USA] 1 tbl/10 ml) inhibitors on ice for 20 min with sonification every 5 min. Cell debris and nuclei were removed by centrifugation (14 000 x g, 10 min; 4 °C) and the supernatant was aliquoted and stored at −80 °C.

Competitive Hybridization in Cell Lysate

A black 140 µl high performance quartz glas cuvette (*Hellma*, Müllheim, Germany) was filled with PBS buffer (10 mM Na_2HPO_4, 100 mM NaCl, pH 7.0) or 100 % HEK293 cell lysate and a blank measurement was recorded. 500 nM of UE-QB, ED-TO (or UE-TO and ED-QB) probe and edited or unedited RNA target were added sequentially. After addition of each oligonucleotide, the concentration was verified by measuring the absorption at 260 nm. Then, TO (λ_{ex} = 485 nm. λ_{em} = 500–700 nm, readout at 535 nm) and QB- (λ_{ex} = 560 nm, λ_{em} = 570–800 nm, readout at 605 nm) fluorescence was measured consecutively at 37 °C.

Table S7 Fluorescence signal of probe pairs before (F_0) and after (F) hybridization with RNA target.

probe pair	Target[a]	QB fluorescence[b]				TO fluorescence[c]			
		Buffer		Lysate		Buffer		Lysate	
		F_0	F	F_0	F	F_0	F	F_0	F
ED-QB-OMe-C1 + UE-TO-OMe-C1s	ED	0.68 ± 0.35	7.49 ± 2.29	2.23 ± 0.43	7.89 ± 1.41	1.77 ± 1.11	3.37 ± 1.28	2.65 ± 0.84	3.68 ± 1.21
	UE	1.39 ± 0.19	1.93 ± 0.66	1.74 ± 0.14	2.99 ± 0.83	3.89 ± 0.47	28.1 ± 8.31	2.56 ± 0.28	18.2 ± 3.41
ED-QB^2-OMe-1 + UE-TO^2-OMe-2	ED	1.07 ± 0.28	36.5 ± 0.72	2.61 ± 0.34	20.8 ± 2.94	6.70 ± 0.19	6.90 ± 0.09	7.96 ± 0.58	12.8 ± 1.29
	UE	1.07 ± 0.28	5.10 ± 0.68	2.61 ± 0.34	5.00 ± 0.23	6.70 ± 0.19	51.3 ± 1.33	7.96 ± 0.58	66.9 ± 3.35
UE-QB-OMe-C1 + ED-TO-OMe-C1	ED	0,72 ± 0.15	14.1 ± 0.42	5.07 ± 0.20	18.8 ± 1.64	4.74 ± 0.12	41.0 ± 0.73	11.0 ± 0.91	34.8 ± 1.28
	UE	0,72 ± 0.15	53.3 ± 0.48	5.07 ± 0.20	46.6 ± 2.21	4.74 ± 0.12	5.60 ± 0.06	11.0 ± 0.91	15.4 ± 0.79
UE-QB^2-OMe1 + ED-TO^2-OMe-4	ED	0.82 ± 0.13	7.40 ± 0.07	1.80 ± 0.55	6.00 ± 0.51	5.94 ± 0.08	31.4 ± 1.66	7.95 ± 0.66	31.8 ± 0.50
	UE	0.82 ± 0.13	33.7 ± 0.26	1.80 ± 0.55	25.6 ± 2.32	5.94 ± 0.08	9.60 ± 0.17	7.95 ± 0.66	11.4 ± 0.12

[a] 5’-CUGAAGGACUCACCCUGC**Y**UCAGUUUAUUU UGA-3', UE: Y = C; ED: Y = U; [b] λ_{ex} = 560 nm, λ_{em} = 605 nm; [c] λ_{ex} = 485 nm, λ_{em} = 535 nm.

Table S8 QB/TO signalling ratio[a] of probe pairs before (ss) and after hybridization with RNA target ED or UE.

probe pair	Target[b]	Buffer	Lysate
ED-QB-OMe-C1 + UE-TO-OMe-C1s	ss	0.4	0.57
	ED	2.22	2.14
	UE	0.07	0.16
ED-QB^2-OMe-1 + UE-TO^2-OMe-2	ss	0.16	0.33
	ED	5.29	1.63
	UE	0.10	0.07
UE-QB-OMe-C1 + ED-TO-OMe-C1	ss	0.15	0.46
	ED	0.34	0.54
	UE	9.52	3.03
UE-QB^2-OMe1 + ED-TO^2-OMe-4	ss	0.14	0.23
	ED	0.24	0.19
	UE	3.51	2.25

[a] calculated from values listed in Table S7;

[b] 5’-CUGAAGGACUCACCCUGC**Y**UCAGUUUAUUU UGA-3', UE: Y = C; ED: Y = U;

7. Partial Hybridization

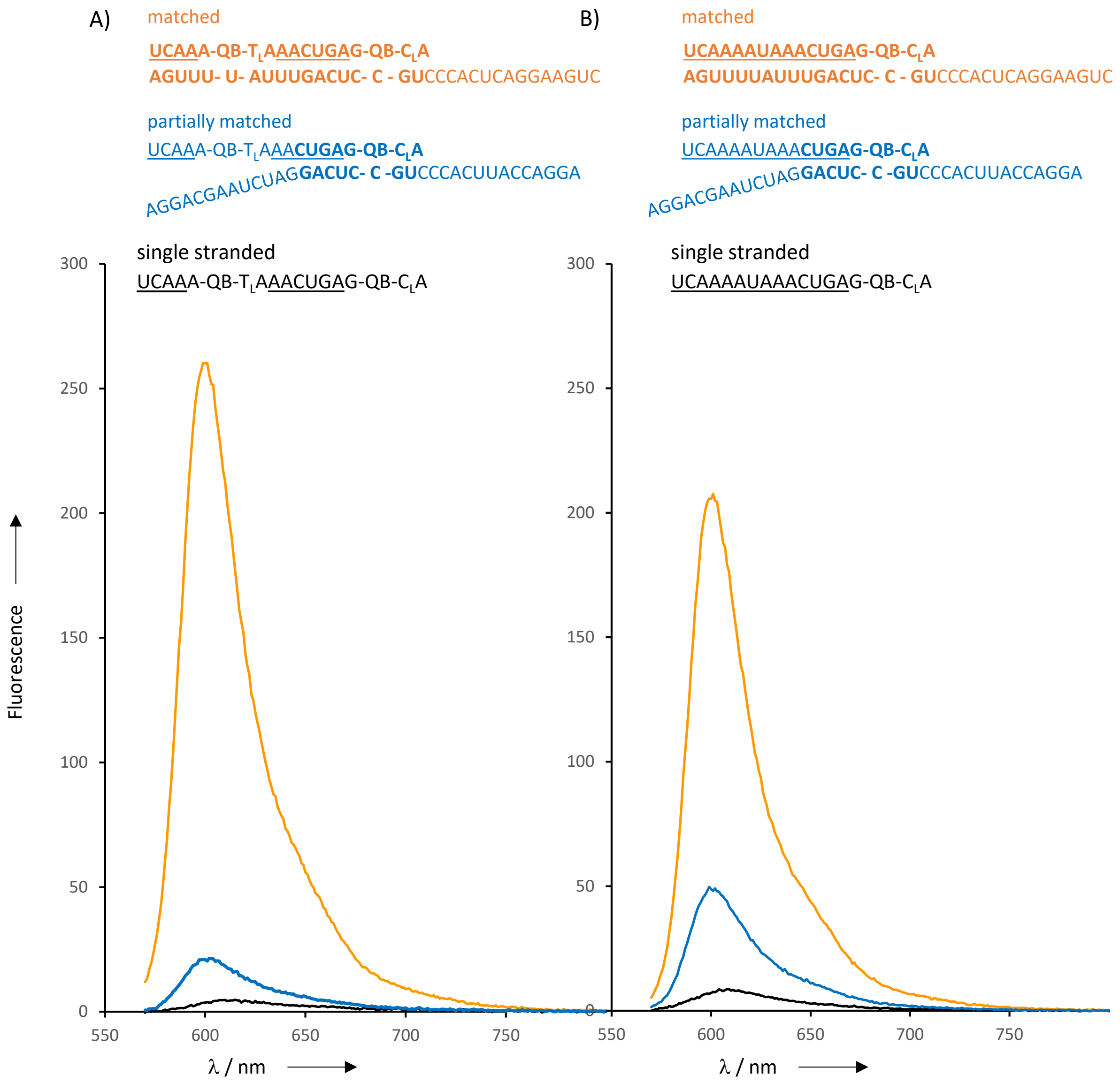

Figure S12 Fluorescence spectra of A) **UE-QB^2-OMe-1** and B) **UE-QB-OMe-C1** in single stranded form, after matched hybridization and after partial hybridization. Compared to the FIT RNA probe UE-QB-OMe-C1 the FIT^2 RNA probe **UE-QB^2-OMe-1** shows higher target specificity i.e. fluorescence upon matched hybridization is higher and fluorescence upon partial hybridization is lower. Underlined = 2'OMe-RNA; subscript L = LNA; bold letters = nucleotides involved in base pairings. Conditions: 0.5 µM probe was measured **at 25 °C** before and after addition of 5 eq. RNA target. λ_{ex} = 560 nm, λ_{em} = 605 nm.

8. MALDI-TOF MS Analysis

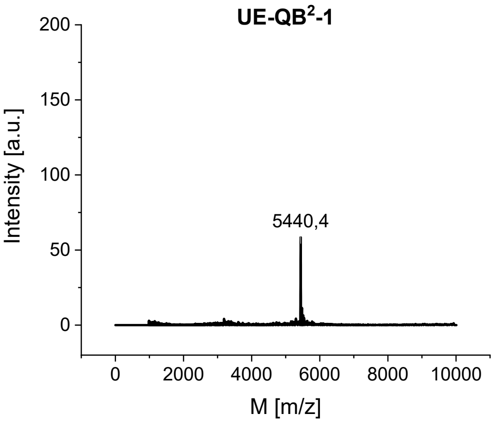

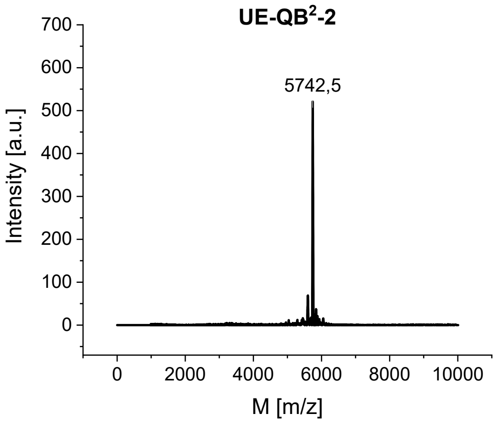

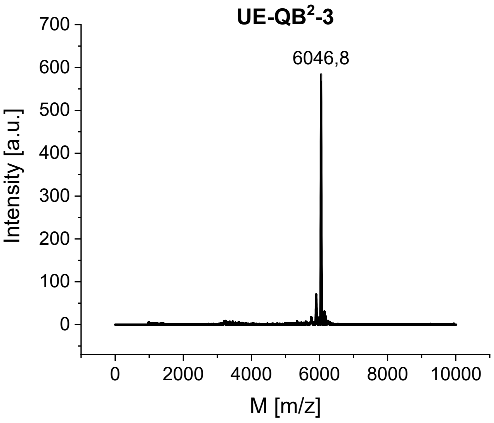

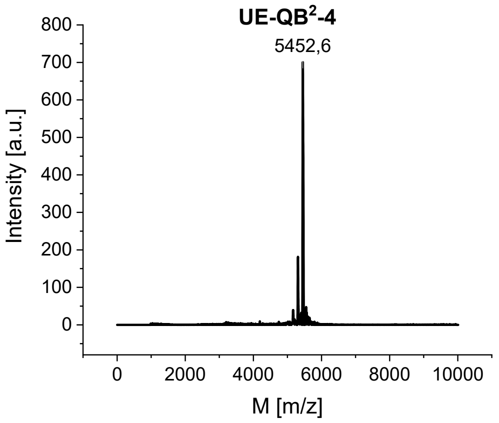

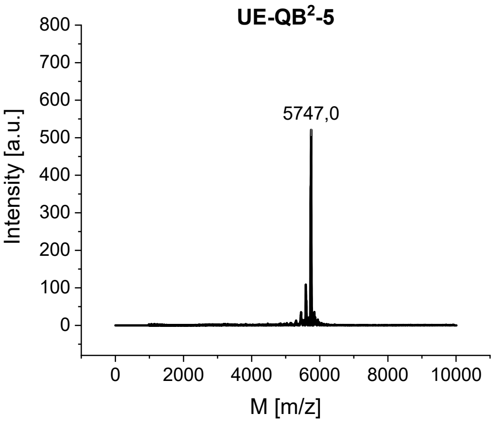

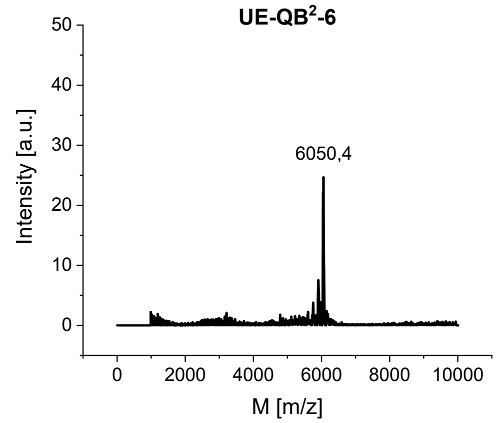

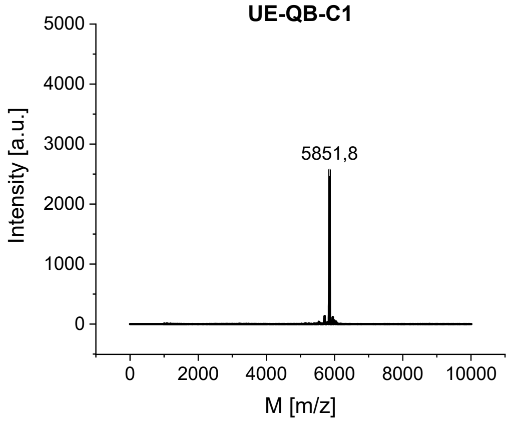
UE-QB-C1
5851,8
Intensity [a.u.]
M [m/z]

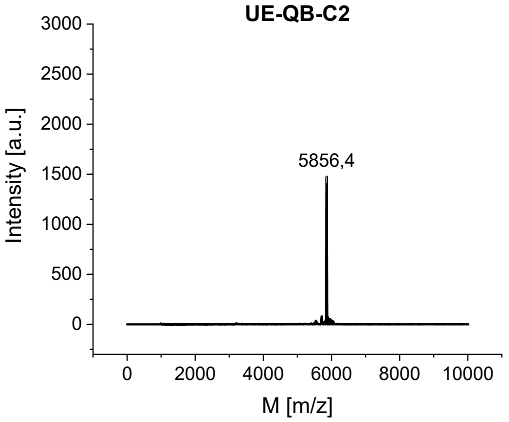
UE-QB-C2
5856,4
Intensity [a.u.]
M [m/z]

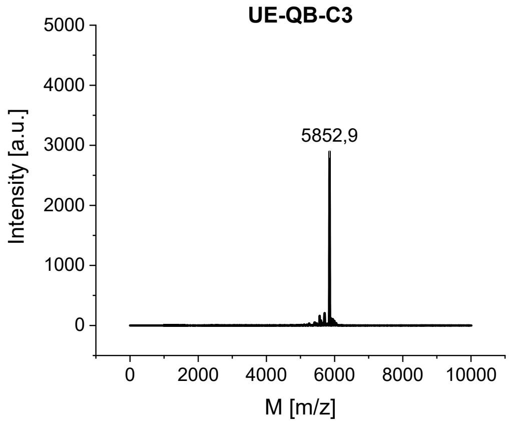
UE-QB-C3
5852,9
Intensity [a.u.]
M [m/z]

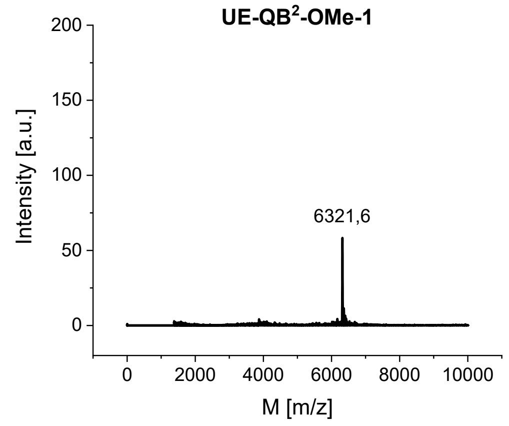
UE-QB²-OMe-1
6321,6
Intensity [a.u.]
M [m/z]

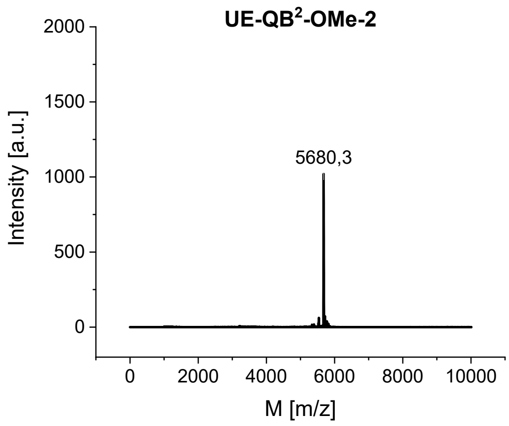
UE-QB²-OMe-2
5680,3
Intensity [a.u.]
M [m/z]

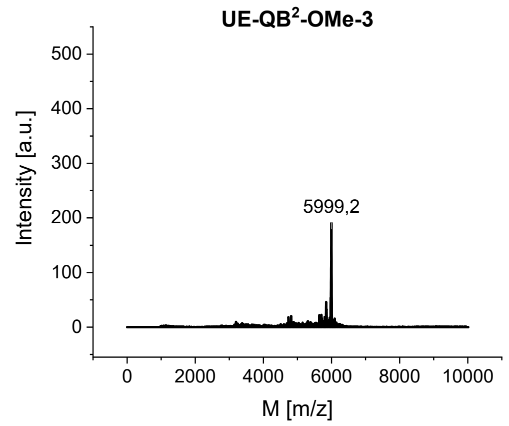
UE-QB²-OMe-3
5999,2
Intensity [a.u.]
M [m/z]

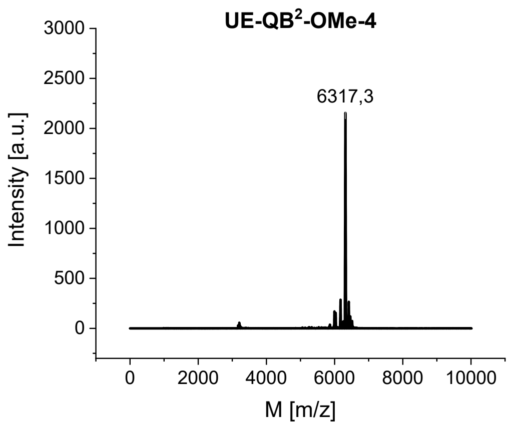
UE-QB²-OMe-4
6317,3
Intensity [a.u.]
M [m/z]

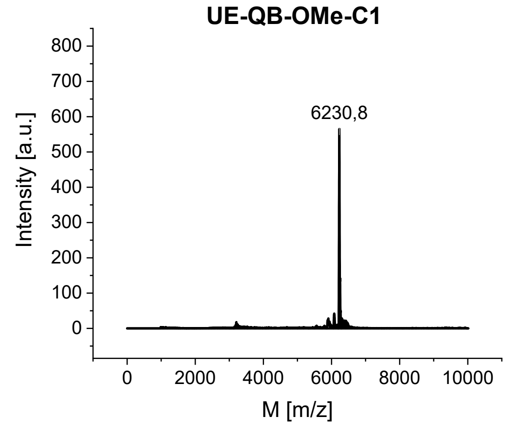
UE-QB-OMe-C1
6230,8
Intensity [a.u.]
M [m/z]

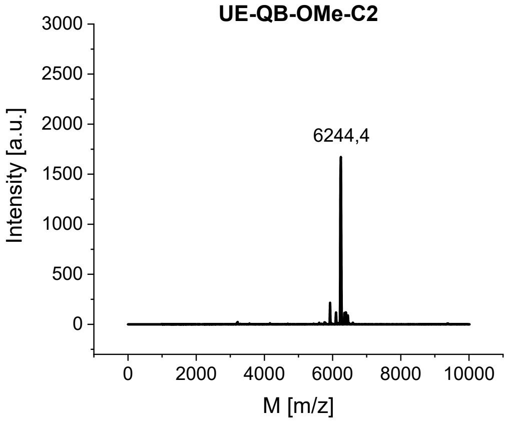
UE-QB-OMe-C2
6244,4
Intensity [a.u.]
M [m/z]

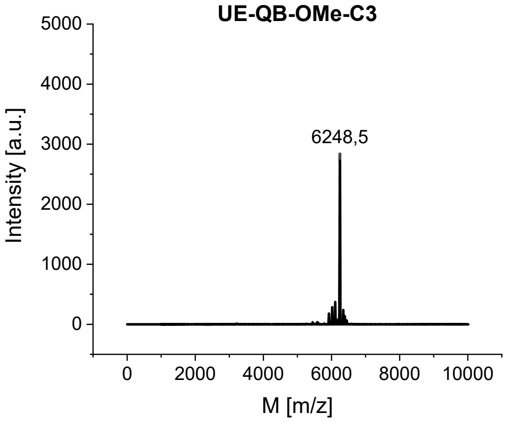
UE-QB-OMe-C3
6248,5
Intensity [a.u.]
M [m/z]

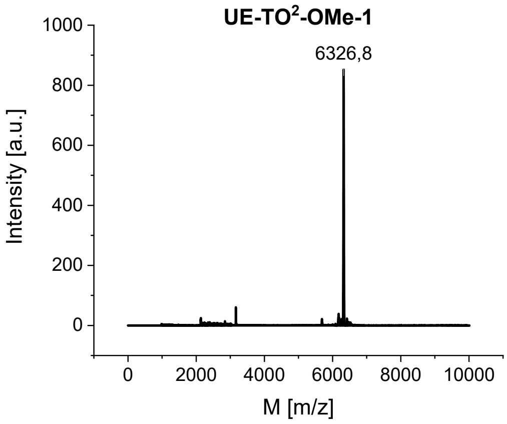
UE-TO^2-OMe-1
6326,8
Intensity [a.u.]
M [m/z]

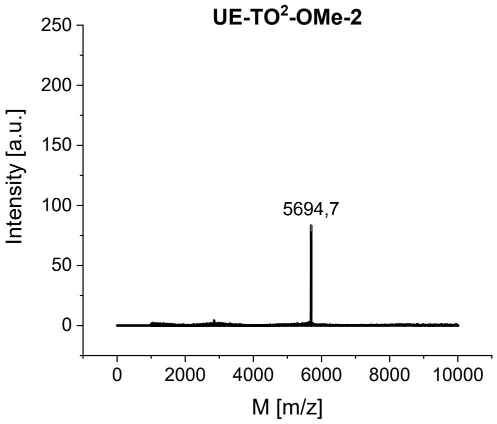
UE-TO^2-OMe-2
5694,7
Intensity [a.u.]
M [m/z]

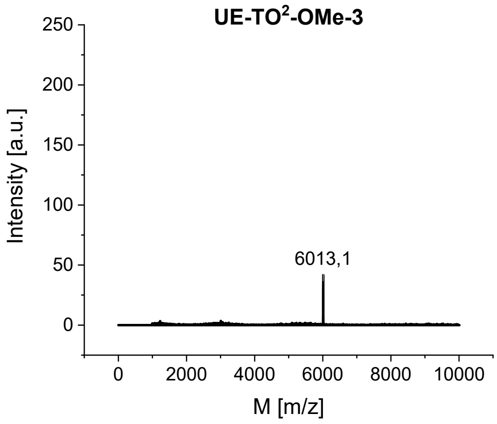
UE-TO^2-OMe-3
6013,1
Intensity [a.u.]
M [m/z]

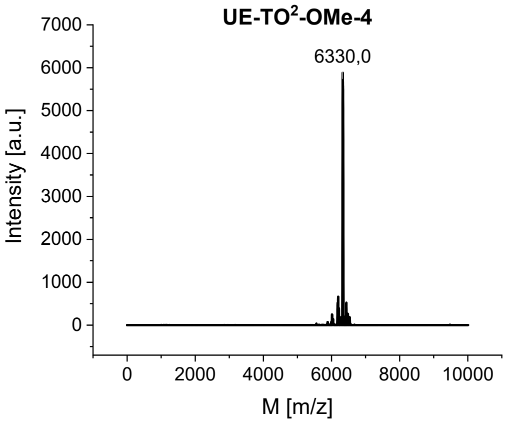
UE-TO^2-OMe-4
6330,0
Intensity [a.u.]
M [m/z]

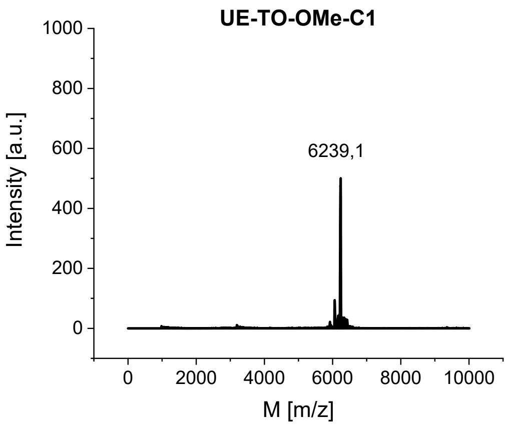
UE-TO-OMe-C1
6239,1
Intensity [a.u.]
M [m/z]

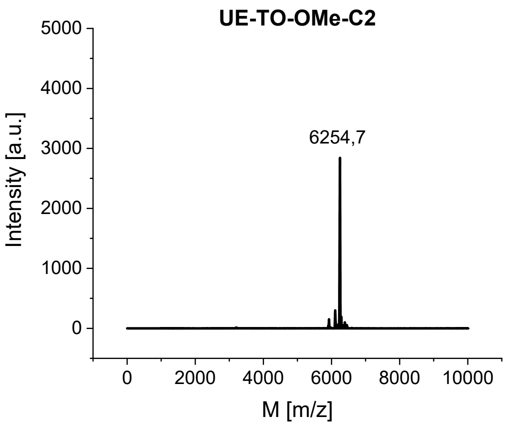
UE-TO-OMe-C2
6254,7
Intensity [a.u.]
M [m/z]

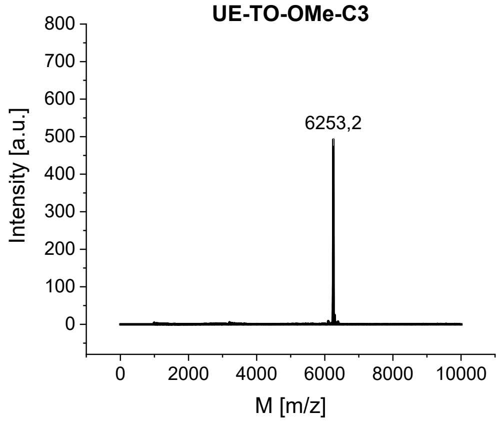
UE-TO-OMe-C3
6253,2
Intensity [a.u.]
M [m/z]
0
100
200
300
400
500
600
700
800
0
2000
4000
6000
8000
10000

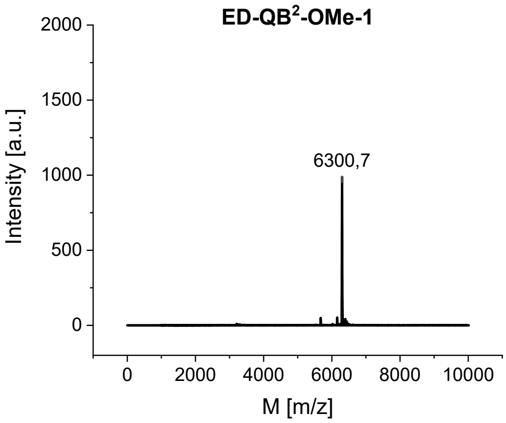
ED-QB^2-OMe-1
6300,7
Intensity [a.u.]
M [m/z]
0
500
1000
1500
2000
0
2000
4000
6000
8000
10000

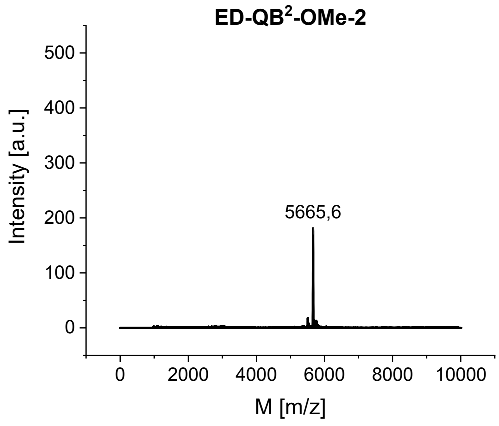
ED-QB^2-OMe-2
5665,6
Intensity [a.u.]
M [m/z]
0
100
200
300
400
500
0
2000
4000
6000
8000
10000

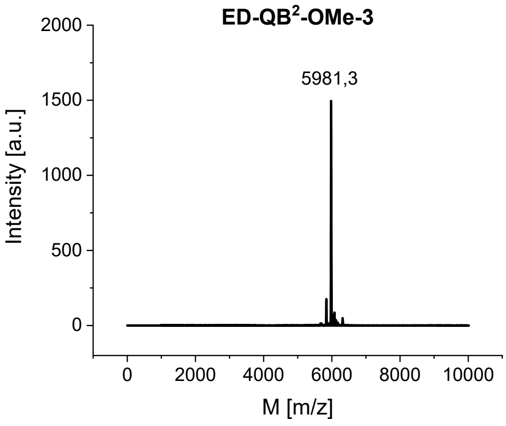
ED-QB^2-OMe-3
5981,3
Intensity [a.u.]
M [m/z]
0
500
1000
1500
2000
0
2000
4000
6000
8000
10000

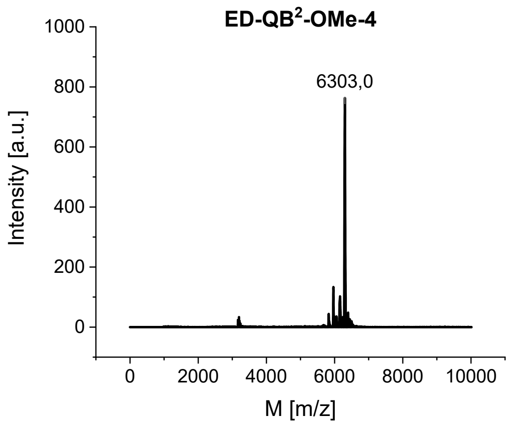
ED-QB^2-OMe-4
6303,0
Intensity [a.u.]
M [m/z]
0
200
400
600
800
1000
0
2000
4000
6000
8000
10000

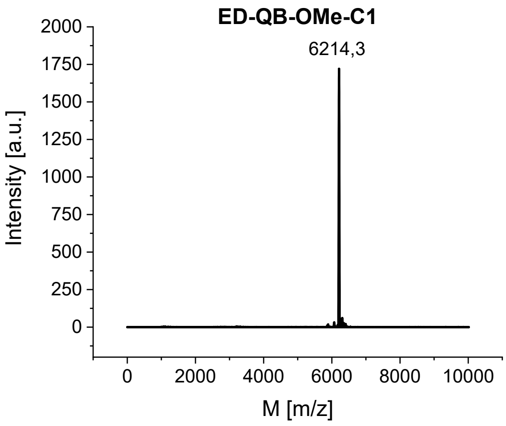
ED-QB-OMe-C1
6214,3
Intensity [a.u.]
M [m/z]
0
250
500
750
1000
1250
1500
1750
2000
0
2000
4000
6000
8000
10000

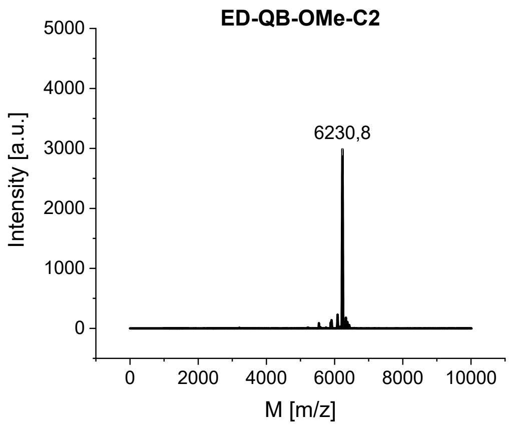
ED-QB-OMe-C2
6230,8
Intensity [a.u.]
M [m/z]
0
1000
2000
3000
4000
5000
0
2000
4000
6000
8000
10000

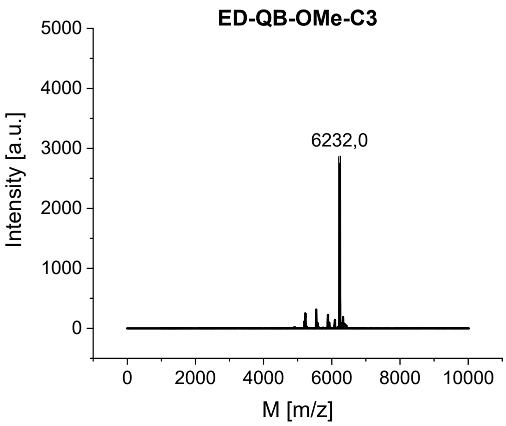
ED-QB-OMe-C3
6232,0
Intensity [a.u.]
M [m/z]
0
1000
2000
3000
4000
5000
0
2000
4000
6000
8000
10000

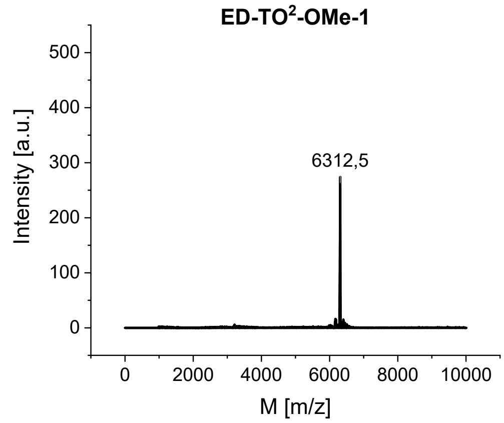
ED-TO²-OMe-1
6312,5
Intensity [a.u.]
0 100 200 300 400 500
M [m/z]
0 2000 4000 6000 8000 10000

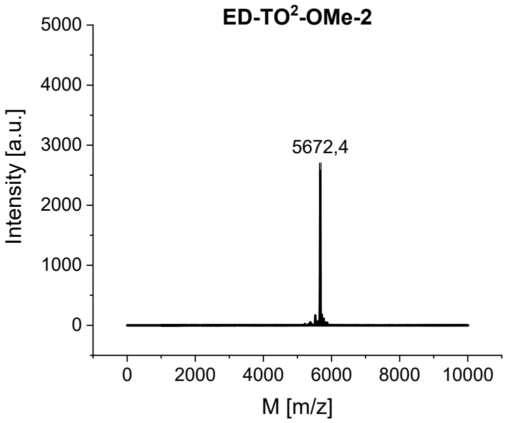
ED-TO²-OMe-2
5672,4
Intensity [a.u.]
0 1000 2000 3000 4000 5000
M [m/z]
0 2000 4000 6000 8000 10000

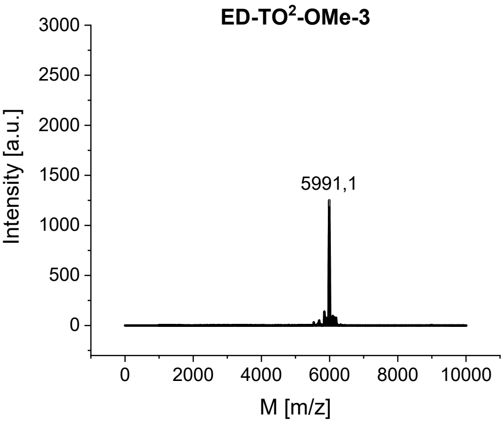
ED-TO²-OMe-3
5991,1
Intensity [a.u.]
0 500 1000 1500 2000 2500 3000
M [m/z]
0 2000 4000 6000 8000 10000

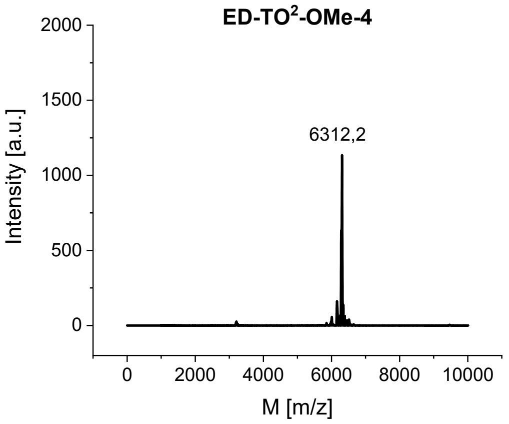
ED-TO²-OMe-4
6312,2
Intensity [a.u.]
0 500 1000 1500 2000
M [m/z]
0 2000 4000 6000 8000 10000

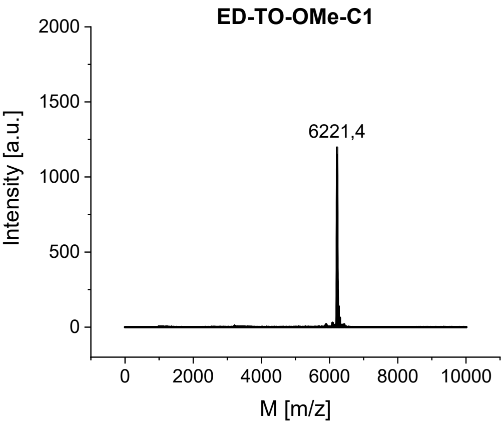
ED-TO-OMe-C1
6221,4
Intensity [a.u.]
0 500 1000 1500 2000
M [m/z]
0 2000 4000 6000 8000 10000

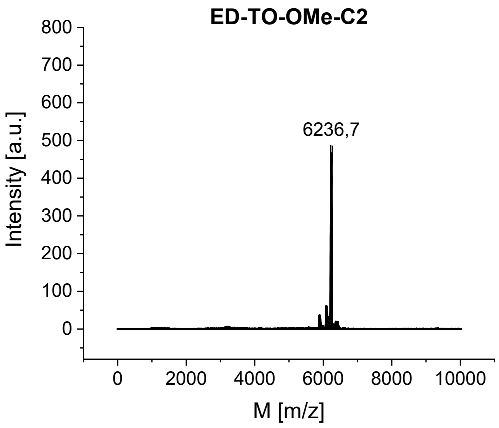
ED-TO-OMe-C2
6236,7
Intensity [a.u.]
0 100 200 300 400 500 600 700 800
M [m/z]
0 2000 4000 6000 8000 10000

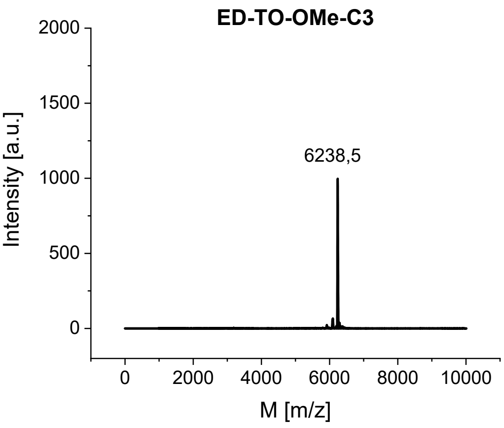
ED-TO-OMe-C3
6238,5
Intensity [a.u.]
0 500 1000 1500 2000
M [m/z]
0 2000 4000 6000 8000 10000

9. HPLC and UPLC Analysis

UE-QB^2-1	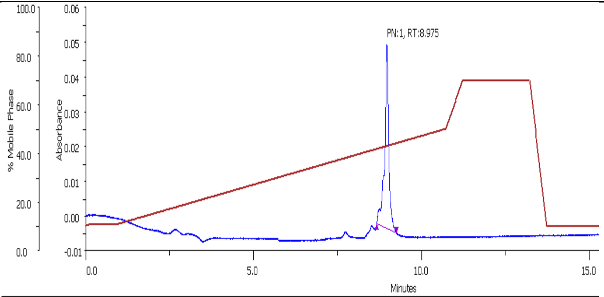
UE-QB^2-2	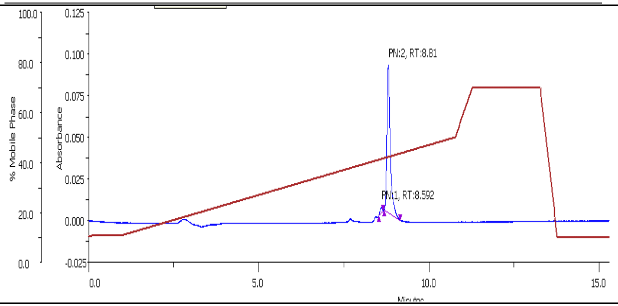
UE-QB^2-3	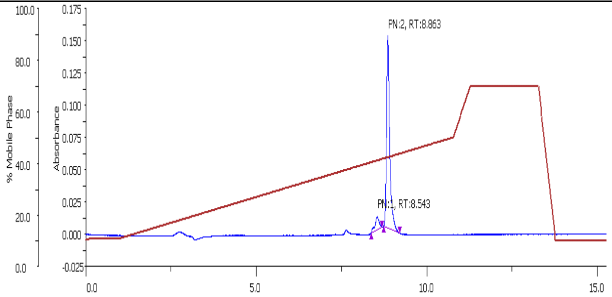
UE-QB^2-4	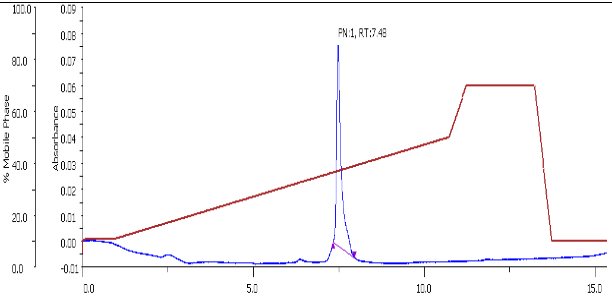
UE-QB^2-5	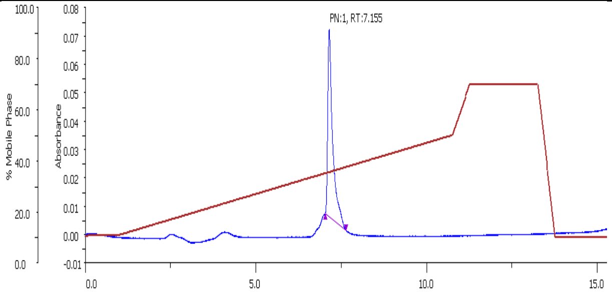
UE-QB^2-6	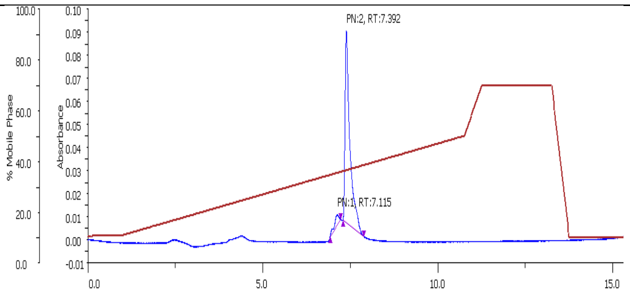

UE-QB-C1	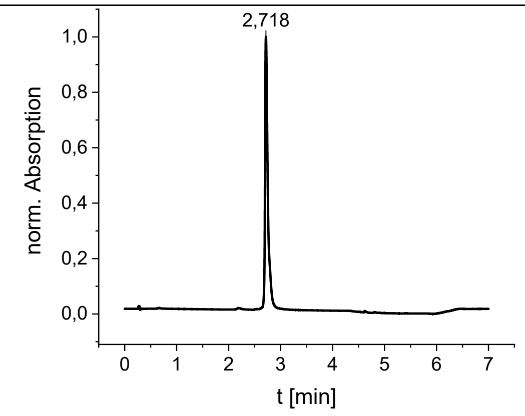
UE-QB-C2	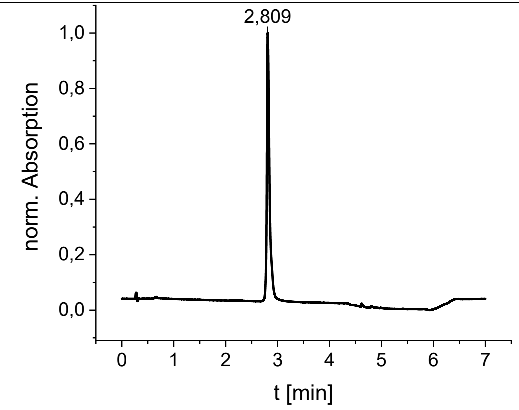
UE-QB-C3	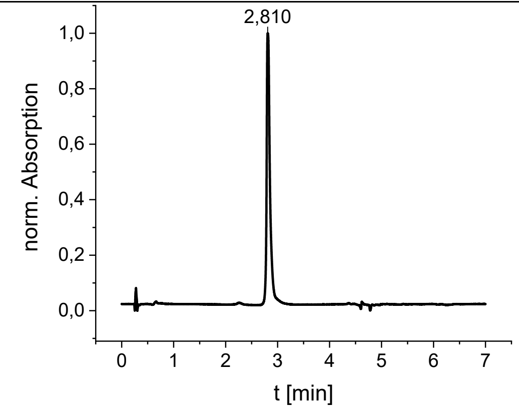
UE-QB^2-OMe-1	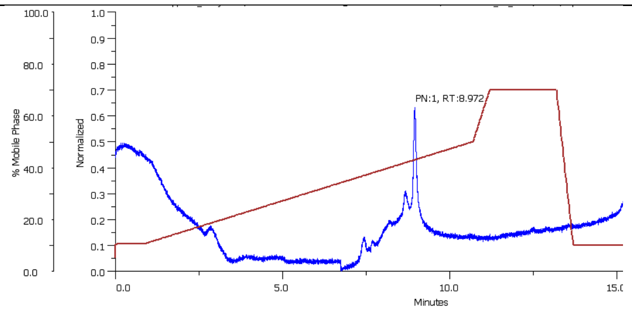 low sample amount
UE-QB^2-OMe-2	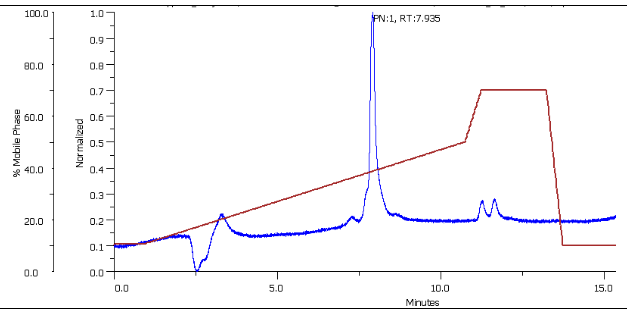

<table>
<tr><td>UE-QB^2-OMe-3</td><td>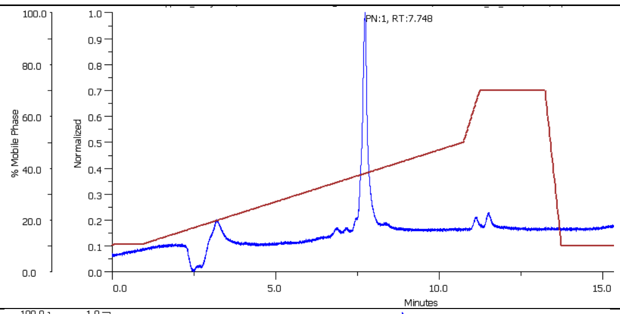
</td></tr>
<tr><td>UE-QB^2-OMe-4</td><td>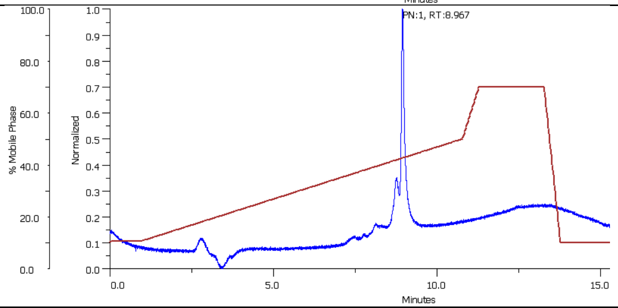
</td></tr>
<tr><td>UE-QB-OMe-C1</td><td>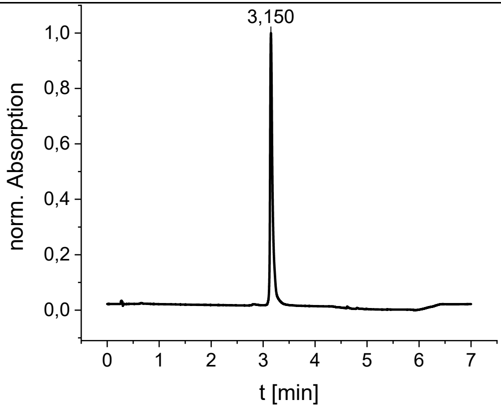
</td></tr>
<tr><td>UE-QB-OMe-C2</td><td>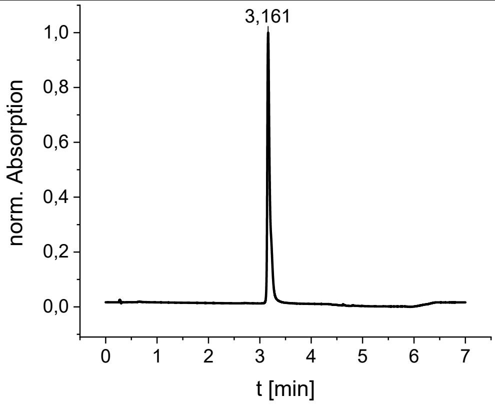
</td></tr>
<tr><td>UE-QB-OMe-C3</td><td>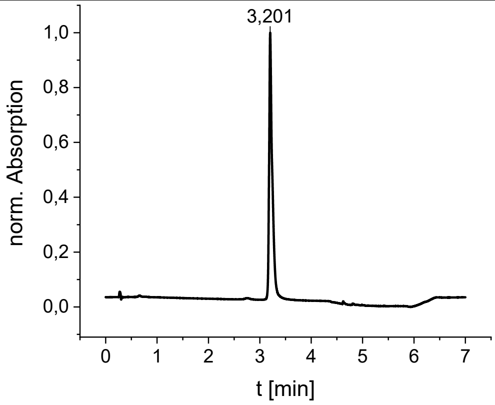
</td></tr>
<tr><td></td><td></td></tr>
</table>

ED-TO^2-OMe-1	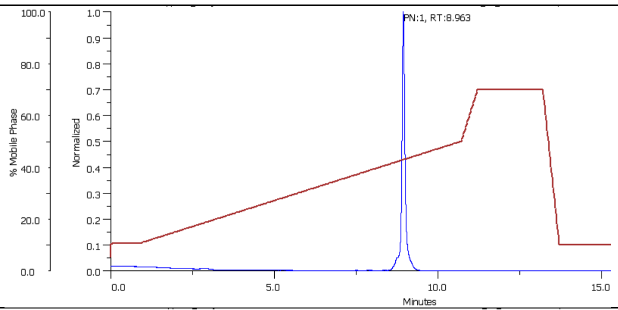
ED-TO^2-OMe-2	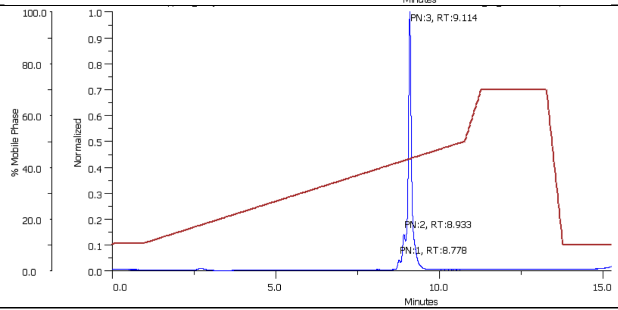
ED-TO^2-OMe-3	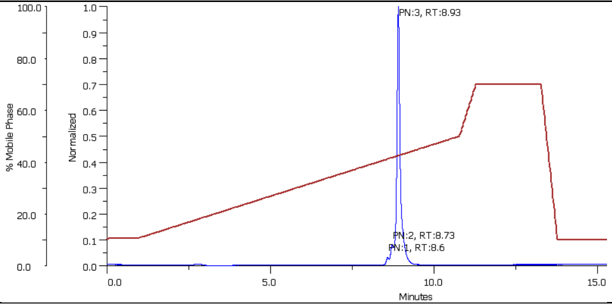
ED-TO^2-OMe-4	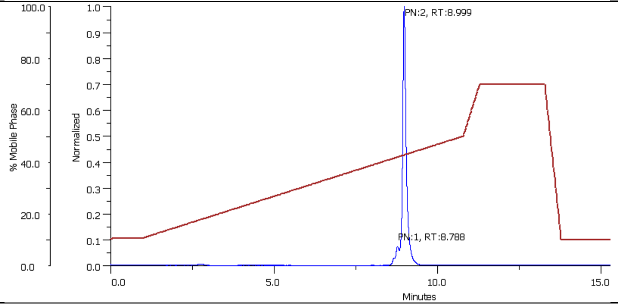
ED-TO-OMe-C1	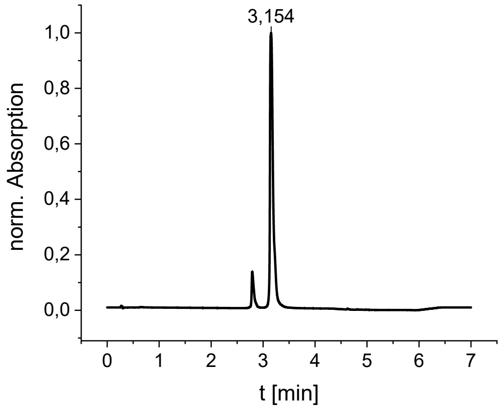

<table>
<tr><td>ED-TO-OMe-C2</td><td>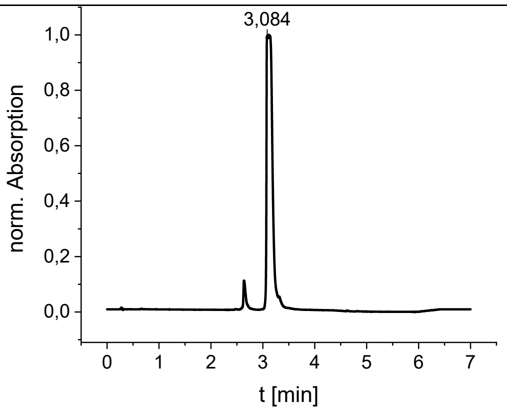
</td></tr>
<tr><td>ED-TO-OMe-C3</td><td>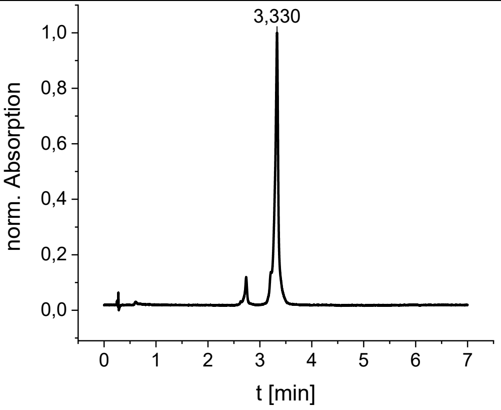
</td></tr>
<tr><td></td><td></td></tr>
<tr><td>UE-TO^2-OMe-1</td><td>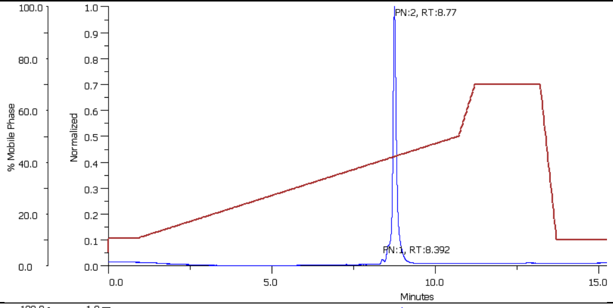
</td></tr>
<tr><td>UE-TO^2-OMe-2</td><td>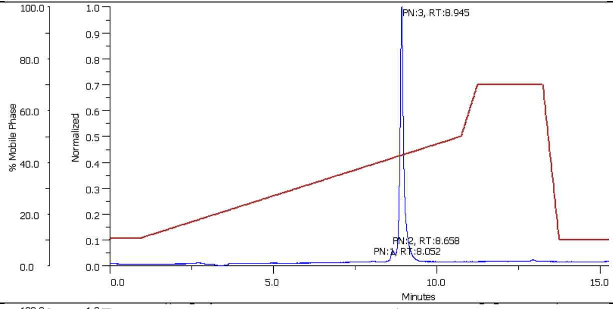
</td></tr>
<tr><td>UE-TO^2-OMe-3</td><td>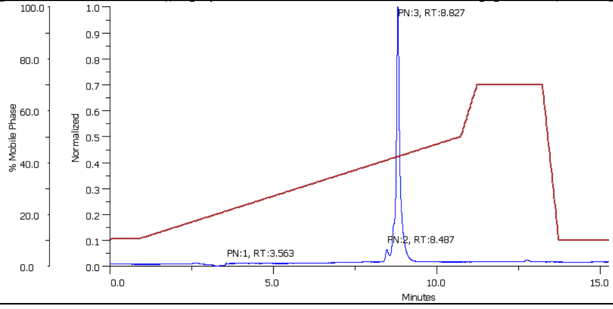
</td></tr>
</table>

UE-TO^2-OMe-4	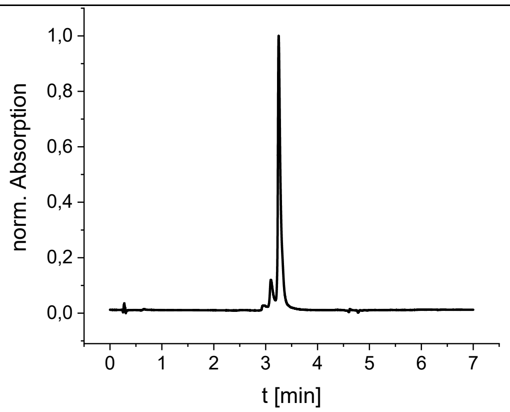
UE-TO-OMe-C1	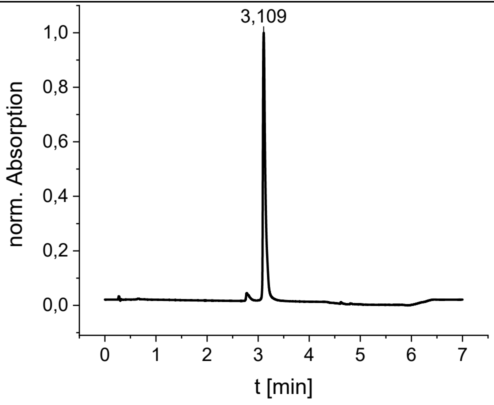
UE-TO-OMe-C1s	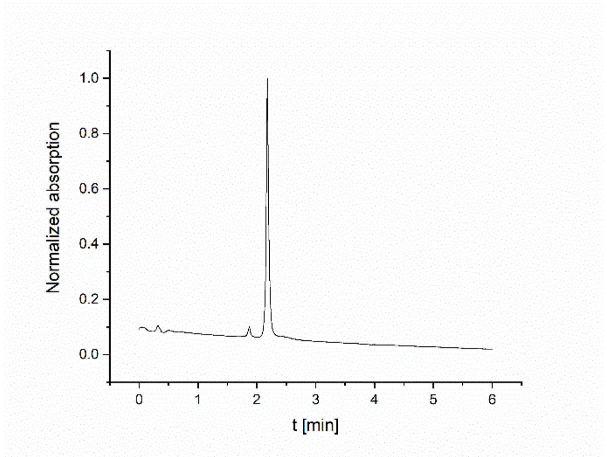
UE-TO-OMe-C2	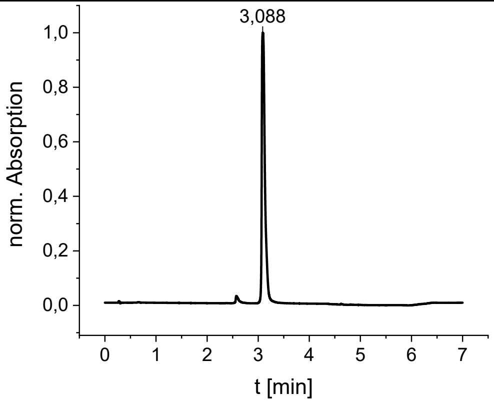

<table>
<tr><td>UE-TO-OMe-C3</td><td>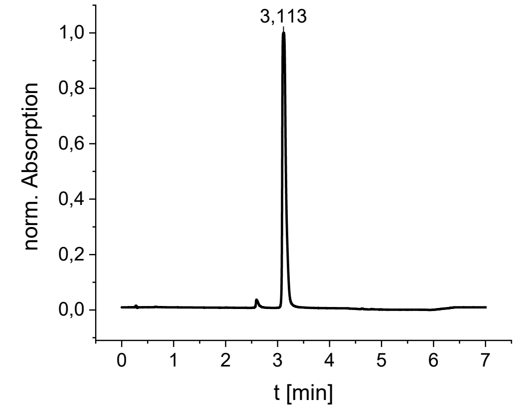
</td></tr>
<tr><td></td><td></td></tr>
<tr><td>ED-QB^2-OMe-1</td><td>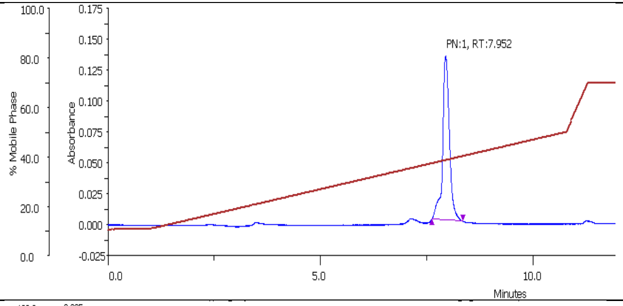
</td></tr>
<tr><td>ED-QB^2-OMe-2</td><td>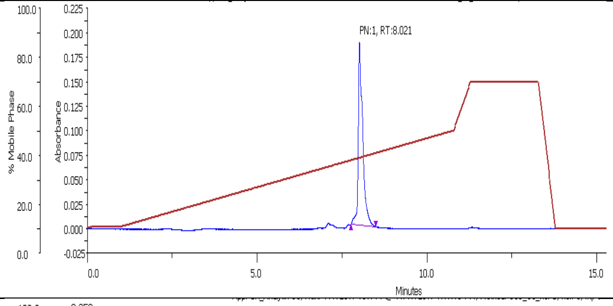
</td></tr>
<tr><td>ED-QB^2-OMe-3</td><td>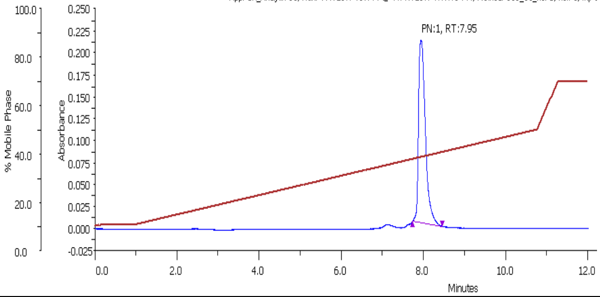
</td></tr>
<tr><td>ED-QB^2-OMe-4</td><td>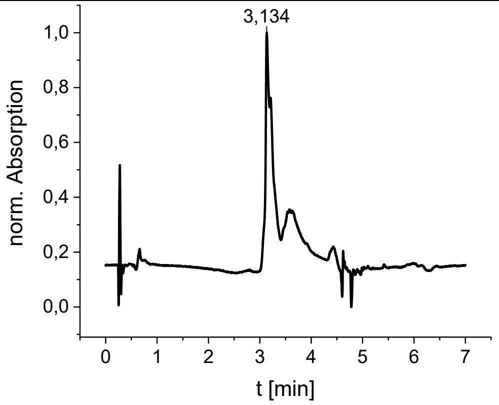

sample amount too low</td></tr>
</table>

ED-QB-OMe-C1	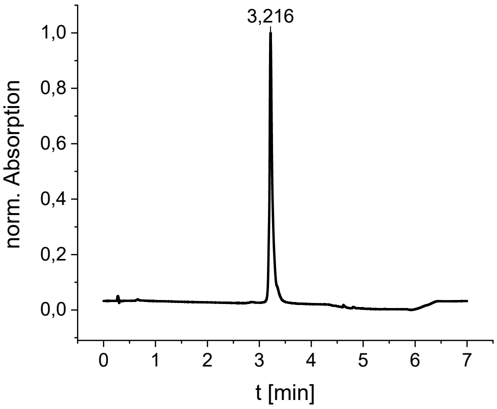
ED-QB-OMe-C2	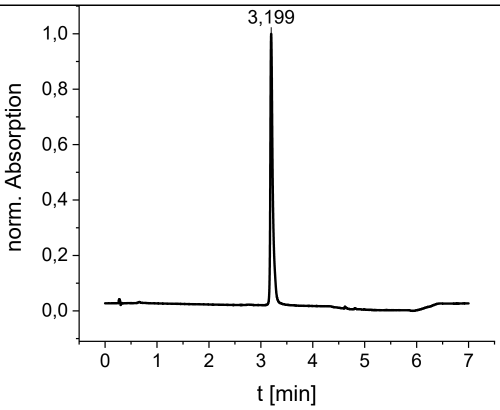
ED-QB-OMe-C3	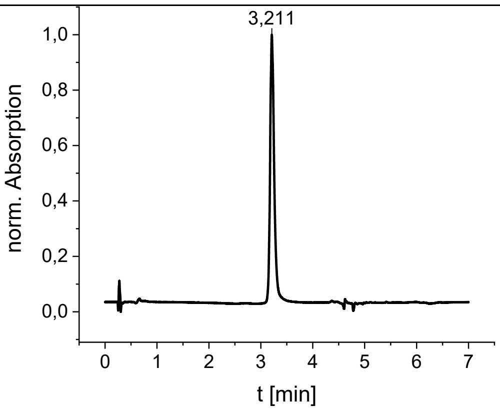

10. References

[1] F. Hövelmann, I. Gaspar, J. Chamiolo, M. Kasper, J. Steffen, A. Ephrussi; Chemical science. 2016, 7, 128-35.
